# Classification of the Biogenicity of Complex Organic Mixtures for the Detection of Extraterrestrial Life

**DOI:** 10.3390/life11030234

**Published:** 2021-03-12

**Authors:** Nicholas Guttenberg, Huan Chen, Tomohiro Mochizuki, H. James Cleaves

**Affiliations:** 1 Earth-Life Science Institute, Tokyo Institute of Technology, Ookayama, Tokyo 152-8550, Japan; ngutten@gmail.com (N.G.); tomo.mochiviridae@elsi.jp (T.M.); 2 Cross Labs, Cross Compass Ltd., 2-9-11-9F Shinkawa, Chuo-ku, Tokyo 104-0033, Japan; 3 GoodAI, Na Petynce 213/23b, 169 00 Prague, Czech Republic; 4 National High Magnetic Field Laboratory, Florida State University, 1800 East Paul Dirac Drive, Tallahassee, FL 32310-4005, USA; huan.chen@magnet.fsu.edu; 5 Institute for Advanced Study, 1 Einstein Drive, Princeton, NJ 08540, USA; 6 Blue Marble Space Institute of Science, Seattle, WA 98104, USA

**Keywords:** astrobiology, life detection, biosignatures, machine learning, mass spectrometry, complexity, prebiotic chemistry

## Abstract

Searching for life in the Universe depends on unambiguously distinguishing biological features from background signals, which could take the form of chemical, morphological, or spectral signatures. The discovery and direct measurement of organic compounds unambiguously indicative of extraterrestrial (ET) life is a major goal of Solar System exploration. Biology processes matter and energy differently from abiological systems, and materials produced by biological systems may become enriched in planetary environments where biology is operative. However, ET biology might be composed of different components than terrestrial life. As ET sample return is difficult, in situ methods for identifying biology will be useful. Mass spectrometry (MS) is a potentially versatile life detection technique, which will be used to analyze numerous Solar System environments in the near future. We show here that simple algorithmic analysis of MS data from abiotic synthesis (natural and synthetic), microbial cells, and thermally processed biological materials (lab-grown organisms and petroleum) easily identifies relational organic compound distributions that distinguish pristine and aged biological and abiological materials, which likely can be attributed to the types of compounds these processes produce, as well as how they are formed and decompose. To our knowledge this is the first comprehensive demonstration of the utility of this analytical technique for the detection of biology. This method is independent of the detection of particular masses or molecular species samples may contain. This suggests a general method to agnostically detect evidence of biology using MS given a sufficiently strong signal in which the majority of the material in a sample has either a biological or abiological origin. Such metrics are also likely to be useful for studies of possible emergent living phenomena, and paleobiological samples.

## 1. Introduction

As humankind explores the cosmos, a principal goal is the detection of life with an independent origin from terrestrial biology. Searching for life in the Universe thus depends on unambiguously distinguishing biological features from abiological background signals, which could take the form of chemical, morphological, or spectral features [1,2,3].

Terrestrial biology is a disequilibrium phenomenon that uses a relatively small set of organic “building block” compounds to channel energy and matter in informationally directed ways to efficiently reproduce itself. These biological organics are themselves the products of the systems they enable. Terrestrial biology observably processes matter and energy differently from abiological systems, and materials produced by biological systems may thus become enriched in planetary environments where biology is operative [3]. 

When organisms die, they release their components in uncontrolled ways into the environment where they undergo decomposition according to kinetically controlled processes [4]. These fresh and aged biological organics thus become potential markers of biological activity.

Many models for the origins of life posit that the environment must have provided organic compounds of sufficient complexity to allow replicating systems to bootstrap themselves into self-propagating systems. How this was accomplished is an open question, but abiological processes are also known to be able to make complex organic compounds [5]. Such abiological organic compounds, which often include compounds and compound classes present in contemporary terrestrial biology, can thus potentially confound searches for ET biology.

MS is a powerful technique for measuring organic compounds in complex mixtures, including those obtained during Solar System exploration for life detection [6,7,8,9,10,11,12], and will undoubtedly play a major role in future space missions to analyze returned samples using high sensitivity and precision instruments on Earth, and in situ analyses, which will likely use lower resolution instrumentation due to the power and weight limitations of spacecraft-based instruments [8,13,14,15]. Such metrics are also likely to be useful for studies of possible emergent living phenomena [16,17], and paleobiological samples [3]. Regardless of the quality of the data, there are currently no completely accepted defining criteria that distinguish biological from abiological organics, and it is possible ET and terrestrial biochemistries could be markedly different [18,19,20]. Thus it is of interest to create measurement criteria that can distinguish the possible origins of detected organics. 

Though a consensus definition of life remains elusive [21,22], and the specific chemical processes that distinguish life from non-life remain unknown, it is likely that life will always present as a phenomenon producing chemical equilibrium faster than the environment, as well as internal chemical disequilibria the environment cannot produce. As organisms grow and produce accumulated biomass under appropriate conditions, they convert environmental chemicals into both their own components and waste, and channel material flux through a small number, relative to what is possible, of discrete (e.g., that bear the hallmarks of natural selection for efficiency [23] and functionality [24]) pathways that assist in their reproduction).

Life in the Universe may commonly be instantiated in organic compounds, which are enormously structurally diverse [25]. This diversity may enable living systems by providing a way to generate almost infinitely tunable structure/property mapping [26]. Given that there are now multiple examples of fundamental artificial “rewirings” of biochemistry [27,28,29], simple searches for terrestrial biomarkers may miss ET biosignatures, and agnostic criteria for detecting evidence of biology may be required to search for life in the Solar System [30].

Searching for life in the Solar System depends on measurements of discrete properties of chemical compounds, though it might also depend on the detection of aggregate properties of the detected cohort [31]. It is sometimes asserted that a hallmark of life is its chemical complexity [32], and there are various ways complexity can be assessed [33]. The individual components of living systems may be comparatively simple in the context of chemical space [34]. Evolutionary optimization may be easily confounded with the way abiological synthesis operates, e.g., simple compounds are made abundantly for kinetic reasons rather than because of metabolic streamlining. For example, glycine is among the most abundant α-amino acids measured in carbonaceous meteorites, and it is also among the most abundant coded protein amino acids [34]. Thus, biology uses the more structurally complex amino acids more sparingly amid an ever-growing possibility space.

Although organisms are largely composed of organic compounds that biology itself constructs, it is also well known that there are a variety of widely distributed abiological processes that also produce organics [35,36,37], and that there is some overlap with regard to the types of species biological and abiological species create [35,38]. This apparent overlap may be partially the result of human investigational bias, as there is a general motivation to report the presence or synthesis of compounds relevant to biology in abiological systems, and it has been shown that often the majority of compounds found in abiologically-derived samples are not likely ones used in terrestrial biological systems [39,40].

The field of “relational biology” examines biology from the standpoint of the “organization of relations” of the components of biological systems at multiple levels [41]. This area of study is based on the notion that complex systems such as biology have complex entailment relations. These entailment relations manifest in the overproduction, relative to less entailed systems, of the materials that the systems that create them use to create themselves, thus “metabolites” and their products should be anomalously abundant in samples containing self-propagating systems such as biology. Whether these materials are persistent over long periods in the environment is another question whose answer depends on surveying chemical kinetics, but it would seem premature to suppose that only presently known types of organic materials can produce living phenomena or that the ones that enable known biology are the most persistent.

**Figure 1:**
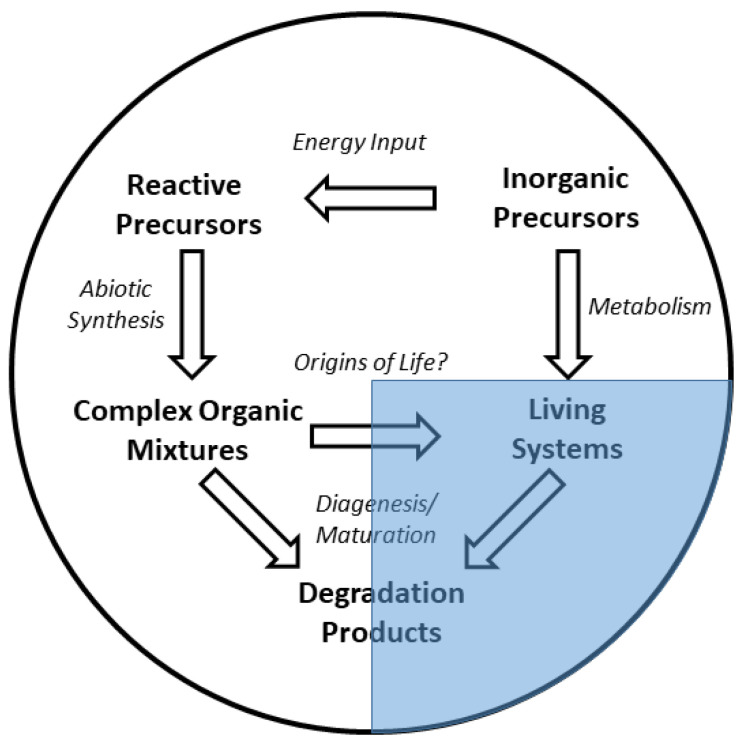
The relationships between abiotic and biological systems with respect to organic compound detection in the environment. According to this schema, biological systems should be classifiable by measurements made in the shaded area.

This added dimension of organic maturation warrants consideration. As organisms die and decompose in the environment, or abiologically derived organics age, their components degrade and inter-react to form new species, often with a general trend of volatile and heteroatom loss, with extreme endpoint products such as graphite, kerogen-like materials, or remineralized carbon (e.g., CO_2_) [4]. A summary of these relationships is shown in Figure 1.

As stated in Cleland [22] “...an operational definition of life that is not founded upon an empirically well-grounded general theory of life is unlikely to tell us much about the nature of life and, even worse, is likely to badly mislead us when it comes to recognizing truly alien forms of life for what they represent.” In other words, living systems composed of partially or wholly different building blocks or metabolisms relative to terrestrial life might be missed entirely by focused terrestrial life compound-based searches. Life may be an example of Wittgenstein’s “family resemblances,” [42]: classes of things may be mutually related by features which are not common to all of the classes.

MS is, in many ways, an ideal technique for detecting extant or extinct organic chemical biosignatures and resolving these issues [8], as biology is complex in the sense of containing many mutually dependent components that can be distinguished based on molecular mass. This idea has a deep history in astrobiology developed in the context of the problem of ET life detection [6,7]. The “standing wave” of molecules that propagate living systems may provide a distinctive fingerprint distinguishable not only from that achievable from stochastic abiological processes, but also from that obtainable from chemical processes that are neither the result of autocatalysis nor natural selection.

We explore here the use of ultrahigh-resolution mass spectrometry to measure a diverse set of biological and abiological complex organic compound suites. These include fresh biological lab-grown micro-organisms (archaebacteria and eubacteria) and laboratory abiotic chemistry simulants (HCN polymer, Miller-Urey experiment products, formose products, caramels, Maillard reaction products and pyruvic acid “polymer,” among others), as well as naturally aged abiological and biological samples (e.g., carbonaceous meteorites and petroleum) and laboratory-aged abiological and biological samples), and the application of machine learning tools to classify their biogenicity as measured by their similarity to the relational mass distributions evidenced by biological materials. We find that even relatively low-resolution parsing of these datasets is able to distinguish biological from abiological samples, which offers a promising avenue for the development of methods for life detection for future Solar System exploration.

## 2. Materials and Methods

### 2.1. General

All glassware and ceramics were ashed at 500 °C to remove organic contaminants. Water was from a Millipore MilliQ system and of maximum conductivity of 18 MΩ. All other solvents were High Pressure Liquid Chromatography (HPLC) grade. Glucose (Reagent Plus Grade, >99.5% purity), glycine (Reagent Plus Grade, >99.5% purity), pyruvic acid, paraformaldehyde (Reagent Grade, ≥94%), NH_4_Cl (ACS Reagent Grade, >99.5% purity), and glycolonitrile (70% solution in water) were purchased from Sigma Aldrich (St. Louis, MO, USA).

### 2.2. Laboratory Abiological Simulants

Various laboratory organic simulants have been proposed as suitable analogues for ET organics or important feedstocks for the origins of life (e.g., [43,44,45,46,47,48]). To broadly survey the molecular products of a wide variety of diversity-generating abiological reactions reaction systems, the following were prepared and analyzed according to methods described along with a brief justification for their inclusion in this study (most samples were prepared newly, though archived laboratory samples were used in the cases of samples 1 and 6, sample 9 was a commercial sample which had aged on our laboratory shelf unopened):

1. Miller-Urey (hereafter referred to as MU) reaction samples prepared as described in [49]. Such reactions have been observed to produce similar amino acid and hydroxy acid distributions as those observed in carbonaceous meteorites [43].
2. The reaction of aqueous NH_3_/HCHO/CH_3_CHO with or without glycolaldehyde (HOCH_2_CHO) as described in [47], which has been shown to produce a variety of amino acids observed in carbonaceous meteorites.
3. NH_3_/HCHO simulants prepared as described in [46], which have been suggested to produce good analogues for the macromolecular organic matter observed in carbonaceous meteorites [46].
4. Maillard reactions of glucose and glycine prepared by the methods of [50], heated either in aqueous solution or the dry state. As modern terrestrial organisms are predominantly composed of protein by dry weight and also contain significant amounts of sugar-containing materials [51], the reactions of these two compounds are perhaps good, though somewhat simplistic, general analogues for thermal alteration of terrestrial biological material.
5. Maillard reactions of glucose and NH_4_OH heated in aqueous solution at 85 °C. As modern terrestrial organisms are predominantly composed of protein by dry weight and also contain significant amounts of sugar-containing materials [51], this model reaction examines the contribution of ammonia species to complex formose reaction products.
6. An NH_4_CN polymerization (hereafter referred to as HCN) prepared according to [49]. HCN chemistry has been implicated in the synthesis of nitrogen heterocycles detected in carbonaceous chondrites [38].
7. Caramelized glucose, which may simulate the thermal alteration of formose-derived sugars, as well as glucose heated in water at 85 °C or 150 °C.
8. Formose reactions prepared from the reaction for aqueous glycolaldehyde and formaldehyde, which have been suggested to produce good analogues for the macromolecular organic matter observed in carbonaceous meteorites [46].
9. A commercial 70% (~13.2 M) aqueous glycolonitrile solution which had been left sealed from the manufacturer on the laboratory shelf at room temperature (~25 °C) for approximately 20 years. This reaction explores the long term outcome of combined HCN/HCHO chemistry.
10. Aqueous or neat pyruvic acid (5.7 M) subjected to heating at 150 °C. Pyruvic acid has been implicated as an intermediate in the abiotic synthesis of tricarboxylic acid cycle components [52].

### 2.3. Petroleum Samples

Bell Creek (Bell Creek, MT, USA) and Exxon 44647 (Shell La Mar, Maracaibo Basin, Venezuela) petroleum samples were dissolved in toluene and diluted with equal parts by volume of methanol spiked with 0.25% (by volume) tetramethylammonium hydroxide (TMAH) to ensure efficient deprotonation [53] for negative-ion Electrospray Ionization Fourier Transform Ion Cyclotron Resonance Mass Spectrometry (ESI-FT-ICR-MS) prior to the FT-ICR mass spectral analysis.

### 2.4. Meteorite Samples

The organic content of meteorites and other ET materials varies as a function of formational and post-formational histories [36,54]. Thus several carbonaceous chondrite (CC) meteorite samples were also measured. Samples of the Murchison (a CM2 CC), Orgueil (a CI1 CC), and Allende (a CV3 CC) meteorites were crushed using pre-ashed agate mortars and pestles and extracted via sonification in MeOH using the methods of [39].

### 2.5. Biological Samples

As Earth’s pervasive biology is generally accepted to be the major source of its non-living organic reservoirs [4], and it is generally accepted that these are derived from biological and abiological alteration of these materials, which has been validated experimentally [55], thermally altered biological samples were studied. Microbial samples were grown from environmental isolates according to the following methods. Mesophilic bacteria *E. coli* were grown in medium Pi5 consisting of 5 g of artificial sea salt (Instant Ocean, Sarrebourg, France), 1 g Bacto tryptone (BD, Sparks, MD, USA), and one g of Bacto yeast extract (BD, Sparks, MD, USA) per liter at 37 °C. Thermophilic freshwater bacteria *Thermus* sp. were grown in Pi5 medium at 70 °C. Hyperthermophilic freshwater archaea *Pyrobaculum* *aerophilum* YKB31 were grown at 90 °C in Pi5T medium, which consisted of Pi5 supplemented with 1 g Na_2_S_2_O_5_ H_2_O. Thermophilic marine bacteria *Rhodothermus* sp. was grown in medium i1515, which consisted of 15 g of artificial sea salt (Instant Ocean), 15 g of NaCl, one g of Bacto tryptone, one g of Bacto yeast extract per liter at 70 °C. Hyperthermophilic marine archaea, *Aeropyrum* *pernix*, were grown at 90 °C in medium i1515T, which consisted of i1515 supplemented with one g Na_2_S_2_O_5_ H_2_O. All cells were grown in one-liter batches, and the cells were harvested by centrifugation at 8000× *g* for 10 min at 25 °C. To simulate long-term aging in the environment, aliquots of cells were transferred into glass ampoules, dried under vacuum, then sealed using a torch under N_2,_ and aged at various temperatures and times in a muffle furnace.

### 2.6. FT-ICR MS

A custom-built FT-ICR mass spectrometer [56] equipped with a 9.4 Tesla horizontal 220 mm bore diameter superconducting solenoid magnet operated at room temperature, and a modular ICR data station (Predator) [57] which facilitated instrument control, data acquisition, and data analysis, located at the National High Magnetic Field Laboratory in Tallahassee, Florida, was used to analyze the samples. Helium gas introduced into the octopole collisionally cooled ions prior to transfer through radio-frequency (RF)-only quadrupoles (total length 127 cm) equipped with an auxiliary RF waveform [58] into a 7-segment open cylindrical cell [59] with capacitively coupled excitation electrodes based on the Tolmachev configuration [60]. Next, 100–150 individual transients of 5.9 s duration were signal-averaged, apodized with a half-Hanning weight function, and zero-filled once prior to fast Fourier Transformation (FT). Data was collected at maximum memory depth of the data station hardware (16 million samples), apodized with a single-sided Hanning apodization, zero-filled to 16 megasamples (16,777,216 samples or 224). An additional zero fill brought the pre-FT data packet to 32 megasamples. Due to increased complexity at higher *m*/*z*, broadband phase correction [61] was applied to the mass spectra to increase the resolution of isobaric species as previously described [62].

Meteoritic, abiological, and biological materials were extracted into MeOH or water as described previously [39]. The sonicated methanolic or aqueous extracts were briefly centrifuged to remove fines. Mass spectra were calibrated with custom-built software (MIDAS) [57]. 

### 2.7. Ionization

ESI Source: Extracts were analyzed by negative electrospray ionization (ESI). Petroleum samples were dissolved in toluene to yield stock solutions at ~1 mg/mL concentration. The samples were further diluted to 500 μg/mL with equal parts (by volume) of MeOH spiked with 0.25% (by volume) TMAH to ensure efficient deprotonation [53]. Sample solutions were pumped through a microelectrospray source [63] (50 μm i.d. fused silica emitter) at 0.5 μL/min by a syringe pump. The sample and the Pierce^®^ LTQ Velos ESI negative ion calibration solution (Thermo Fisher Scientific, Waltham, MA, USA) were electrosprayed consecutively by use of a dual needle electrospray source [64]. Predator software controlled the duration when each needle was positioned at the entrance of the mass spectrometer. Conditions for negative ion formation were emitter voltage, −2.5 kV; tube lens, −250 V; and heated metal capillary current, 5.0 A.

Negative ESI tends to favor the detection of compounds containing functional groups that can readily lose a proton, such as alcohols, carboxylic acids, cyanides, nitric- and sulfonic-acids. Positive ion ESI data were not collected here. Based on previous data regarding Dissolved Organic Matter (DOM) functional group characteristics, most previously published works on DOM molecular composition have been carried out using negative ion ESI when combined with FT-ICR MS. A previous study on extraterrestrial organic matter in the Murchison meteorite found that positive and negative ESI modes were complementary, and spectra collected in both modes show repetitive patterns [39]. Investigation of speciation by positive-ion ESI, which targets basic functionalities, such as amines, amides, and peptides, could provide additional information and we will expand into this analytical analysis in future work.

### 2.8. Mass Calibration and Data Analysis

ICR frequencies were converted to ion masses based on the quadrupolar trapping potential approximation [65,66]. Each *m*/*z* spectrum was first externally calibrated by the Pierce® LTQ Velos ESI negative ion calibration solution, and internally calibrated based on the “walking” calibration equation [67] for several highly abundant homologous series (mass of –CH_2_– repeating unit 14.01565 Da) confirmed with isotopic fine structure. Mass spectra with *m*/*z* from 150 to 750 were exported to peak lists at a signal-to-noise ratio (S/N) > 2. Elemental compositions assignment and data visualization were facilitated using PetroOrg© software [68].

Representative spectra and Kendrick plots for the measured samples are provided in the SI.

### 2.9. Computational Methods

The raw MS data was simply a peak list including FT-ICR MS-determined mass, peak intensity, and computed Kendrick Mass Defect (KMD) series (computed as the nominal mass minus the Kendrick exact mass based on the mass of CH_2_), separated into odd and even masses. One way to categorize these samples would be to identify each peak or set of peaks with specific compounds, fragments, or repeated motifs (such as KMD or polymer series). However, in the absence of corroborating multi-dimensional spectral analysis and/or comparison with reference spectra, this could be a premature assumption, and the data contain a large number of low abundance peaks that could contain informational structure useful for classification.

To extract this structure, the mass peak lists were converted into 3D histograms with mass, log (relative abundance), and KMD axes. The original mass range of the data from *m*/*z* 150 to 750 was arbitrarily divided into 32 equal bins to produce the mass axes. The cutoff of 750 amu was chosen somewhat arbitrarily, but there is generally peak signal intensity using MS in this *m*/*z* range in these samples. This may be biased by the detector response or the propensity of species to volatilize over this mass range; nevertheless, it is a commonly observed phenomenon.

To compute the abundance axis, peak intensities associated with each of the peaks *a_i_* were then computed: (Equation (1))

(1) $$y_{i}=\frac{\log\left(a_{i}\right)-\langle \log\left(a\right)\rangle +0.5}{9.2}$$

Values of this variable between 0 and 1 were then again mapped into 32 bins to form the abundance axis of the histogram. The constants (0.5 and 9.2) were chosen in order to fit both the highest peaks and the noise floor of the instrument into the histogram, while subtracting the mean log abundance helps calibrate between samples. For the KMD axis, KMD values from −500 to 500 were assorted into five equal-sized bins (e.g., ranging from −500 to −300, −300 to −100, −100 to 100, etc.). After producing the histogram of raw peak counts in each bin *n_ijk_*, to resolve both faint structures corresponding to a small number of peaks and structures corresponding to large numbers of densely clustered peaks, we first normalized over the mass range: (Equation (2))

(2) $$N_{ijk}=\frac{n_{ijk}}{\sum_{i}n_{ijk}+\varepsilon }$$

where ε =10^−8^ is a numerical stabilizing factor in case the denominator is otherwise zero. We then performed a truncated log transform on the histogram values: (Equation (3))

(3) $$X_{ijk}=0.5\log\left(0.1+N_{ijk}\right)+1.25$$

The numerical constants in this expression ensure that all values of X lie in the range ~[0.1, 1.3]. Each sample X can now be treated as a 32 × 32 × 5 ‘image’ for the purpose of visualization or for the application of subsequent statistical methods.

## 3. Results and Discussion

### 3.1. Raw Data Analysis

**Figure 2:**
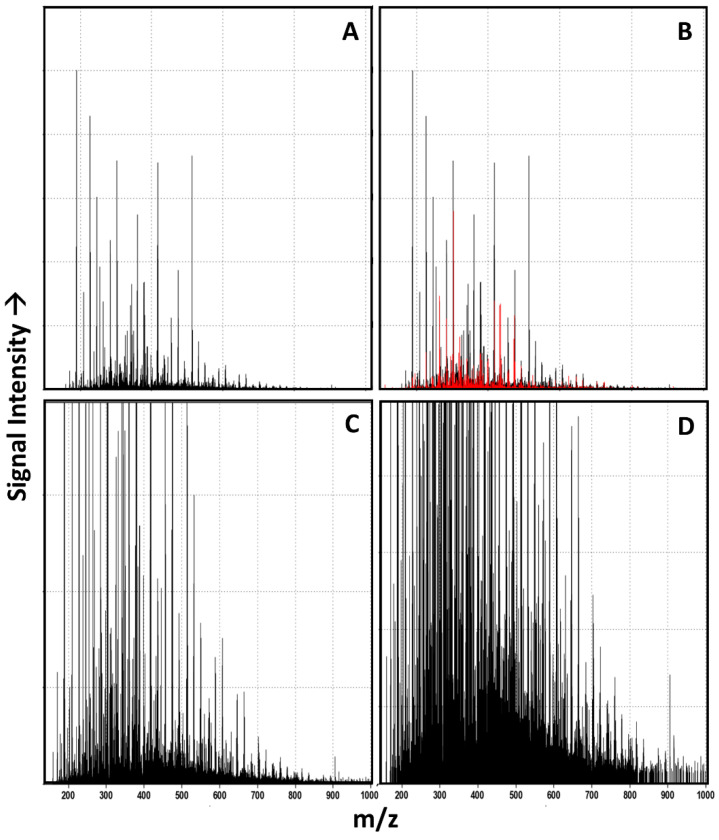
Negative mode FT-ICR MS spectra of the aged aqueous glycolonitrile solution. (**A**). Full scale spectrum (**B**). Full scale spectrum showing unassigned peaks overlain in red. (**C**). and (**D**). 5× and 20× zooms, respectively, showing the density of *m*/*z* signals.

All of these samples are complex in that they contain thousands of unique *m*/*z* peaks resolvable using FT-ICR MS (a representative example is shown in Figure 2).

Although molecular formulas cannot be confidently assigned to ~28% of the 6801 peaks identified in this sample (Figure 2B), various mass ladders are generally evident in these spectra (see Figures S1A–S27B, which point to the operation of the underlying synthetic chemistry, as well as whatever underlying features of the mixtures govern their ability to ionize using the applied analytical methods). It should be noted that the Kendrick plots in the SI are not intensity-thresholded, though peak intensity is a feature evaluated by the algorithms. Thus, what appears to be considerable noise in the spectra or Kendrick plots likely does not contribute markedly to classification. For example, vertically aligned data features apparent in the Kendrick plots are likely instrument noise which, due to low relative intensity, does not contribute significantly to classification.

### 3.2. Visualization of Binned Data

**Figure 3:**
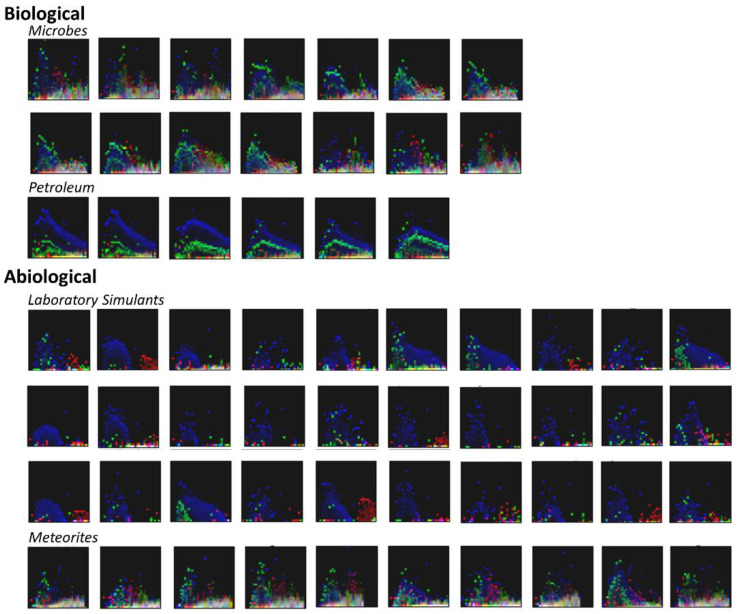
Plots of binned and normalized spectra for the samples studied here. The X-axes show the 32 binned values over the range of *m*/*z* = 150–750, the Y-axes show the 32 binned normalized signal intensities. Colors represent KMD-binned data points, with bins 2, 3, and 4 represented as red, green, and blue, respectively; when the values for the KMD-binned data points are similar the colors blend or appear white. The choice of these three KMD bins for the visualization provided by this figure is arbitrary, there are multiple permutations by which three out of five KMD bins could be presented. This figure presents only a subset of the binned data considered by the algorithms to show how the data is condensed before classification. The individual sample source data for these plots can be found in the SI.

Examining these images directly (visualizing for example, KMD bins 2, 3, and 4 as red, green, and blue, respectively; when the intensity of these are similar, the colors blend or appear white), distinct mass and abundance patterns are visible (see Figure 3). In petroleum samples, there are clear bands of peaks at closely related abundances in particular KMD value bins, followed by an “echo” with the same general structure but at lower abundance with a distinct KMD value. Among the other types of samples, some have a low mass hump of peaks at varying abundances but all with similar KMD values, and a high mass hump of peaks with distinct KMD values. Many of the most distinct patterns occur either primarily at low abundance, or are contained within the distribution of abundances associated with a given mass bin.

### 3.3. Principal Component Analysis

**Figure 4:**
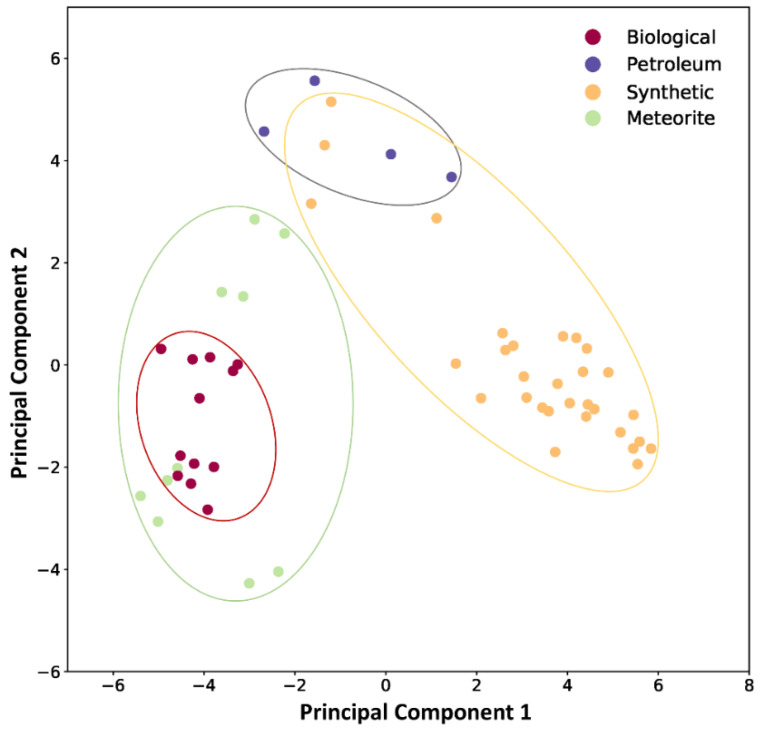
PCA analysis of the analyzed samples. There are two points for each sample due to the separation of identified even and odd mass species. Generally these clusters are relatively close to each other, sometimes overlapping so can be indistinguishable at this level of detail. The apparent spatial distance in this representation does not necessarily coincide with the similarity clustering achieved using the machine learning process, and the ellipses are added merely as guides for the eye.

To identify these features more objectively, principal component analysis (PCA) was performed directly on the 32 × 32 × 5 images associated with the samples. Each image was flattened into a set of 5120 (= 32 × 32 × 5) values, and PCA (using Scikit-learn [69]) was applied. The first two components are visualized in Figure 4 for the studied samples.

Fresh biological samples clearly cluster to the lower left of Figure 4, whereas laboratory simulants cluster on the right-hand side. Laboratory simulants cluster more closely with petroleum samples at this level of analysis, while meteorite samples cluster closely with “fresh” biological samples. Abiological samples (laboratory simulants and meteoritic organics) are more heterogeneous with respect to these two principal components, but still exhibit observable and classifiable similarity. At this level of visualization, this clustering is likely due to the diversity (number of discrete peaks) and heterogeneity (*m*/*z* spacing) of the samples. This can be attributed to the ways reaction types or compound types supported by the chemistries operative in these samples lead to their composition, which is what one might expect to differ between biological (highly entailed) and abiological (less entailed) processes. It should be made clear there are additional levels of principal component analysis which are not visualized in a simple XY plot, thus samples which appear similar at the level of representation presented in Figure 4 are differentiated more deeply by the classifier used here.

### 3.4. Machine Learning Sample Classification

Biologically catalyzed synthetic reactions, e.g., those which occur in biology, steer reactions in highly specific ways compared to abiological synthetic reactions, and once either type of process ceases, abiological reactions degrade the resulting materials in ways that still preserve the biological or abiological hallmarks of their origin.

For example, diversity-generating laboratory simulations of abiological processes (e.g., formose reactions, HCN polymerization products, or Miller-Urey type reactions) often involve iterative application of common reaction mechanisms to common structural motifs (e.g., aldol condensations and ɑ, β elimination reactions in formose chemistry). This kind of chemical process often produces mass “ladders” and other types of repetitive motifs, which are readily visible in both the mass spectra and KMD plots of these reactions (see Figures S1A–S27B).

Likewise, petroleum is highly enriched in lipidic materials, which are themselves highly diverse to begin with in biological material [70], but which may become even more diverse during their abiological degradation. The similarity overlaps observed in Figure 3 suggest there may be temporal degradative development of MS features, which may enable, or conversely confound, the identification of distinctive molecular patterns and features useful as diagnostics of living systems.

The MS data was arbitrarily binned to roughly *m*/*z* = 20 (specifically 18.75) resolution, and individual high-intensity peaks in the original spectra would thus not on their own contribute significantly to the intensity values of the histogram representation shown in Figure 2, so individual compounds can no longer be reconstructed from this representation of the data. To check whether this type of analysis still captures information about the distinction between the different samples, we assigned each sample to one of four categories: Biological, Meteorite, Petroleum, and Synthetic, and then trained a multi-class logistic regression classifier from Scikit learn (https://scikit-learn.org/stable/modules/generated/sklearn.model_selection.KFold.html#sklearn.model_selection.KFold, accessed on 1 April 2017) on the PCA data to determine the degree to which these labels could be predicted from the histogram.

**Figure 5:**
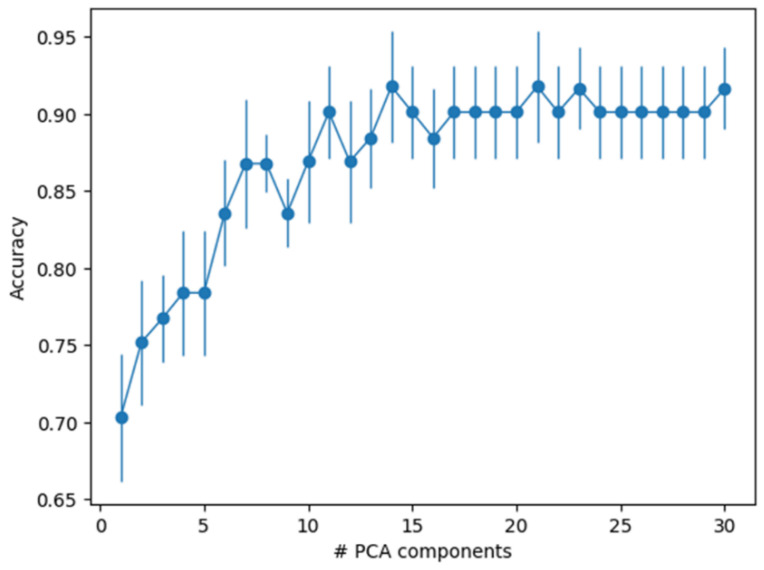
Average test set accuracy and error bars across the five-fold cross-validation as a function of the number of PCA components used. Error bars are a function of the available data, using half the data the error bars should become larger by the square root of 2.

For these data, chance accuracy is 50% as we had significantly more synthetic samples available than natural ones (19 vs. 11). We took the 56 mass spectral data sets and randomly divided them into five “folds,” each consisting of a training set, consisting of 48 samples, and a test set composed of 12 samples. Five folds is a standard-use value, three is generally considered a minimum, and using more than five rarely improves discriminatory efficiency. This was done using Scikit-learn StratifiedKFold, which assigns samples in order to try to preserve the ratios of class labels. We performed N-component PCA on the training set and transformed both training and test data into the PCA component space. Then, we used Scikit-learn LogisticRegression with default hyperparameters (https://scikit-learn.org/stable/modules/grid_search.html, accessed on 1 April 2017) to fit the training data and predict the test data. Figure 5 shows the average test set accuracy and error bars across the 5-fold cross-validation as a function of the number of PCA components used. As can be seen, the classifier can achieve up to 92% test set accuracy based on retaining 15 PCA components using only these reduced (e.g., binned) data, and performs significantly better than chance even using much smaller numbers of PCA components.

**Figure 6:**
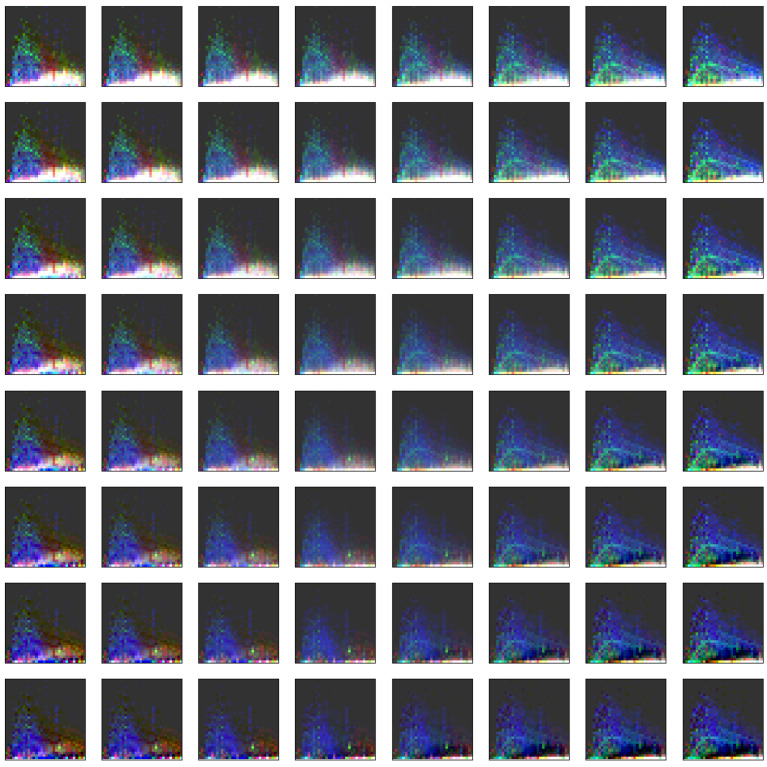
Simulated spectra constructed using the extremes of the first two PCA components determined from the measured spectra (X-axis: principal component 1, Y-axis: principal component 2). The coloring is equivalent to that shown in Figure 3.

The first two PCA components can be used to reconstruct model images similar to those shown in Figure 3 which show the range of extremes the classifier considers. A set of 64 such model spectra are shown in Figure 6.

In this figure, each corner represents an extremum of the combined first and second principal components, and the overall plot represents a “map” of the spectral space interpreted by the classifier. For example, the “petroleum-like” space falls at the far right in this matrix of simulated spectra. This figure points to the notion that a continuous range of spectra can be generated which display some mixture of the features of any given set of samples, and this may be a useful tool for attempting to “fool” new types of classifiers.

### 3.5. Usefulness of Relational Binning Data to Detect Underlying Chemical Patterns

Though FT-ICR-MS is capable of resolving masses to a few ppb over this mass range, this high-resolution data was binned here to ~20 m/z units, which suggests instrument resolution may not be a bottleneck for this classification method. These data clearly show the presence of repetitive structural motifs in the form of the KMD bins, which likely arise in the case of petroleum samples from the preferential preservation of lipids and their modification products, and in the case of the laboratory simulants from the iterative nature of the chemical reactions that produce them (e.g., aldol condensations, cyanide polymerization, Strecker syntheses, etc., see above). 

The data presented in Figure 3, Figure 5 and Figure 6 suggest ultrahigh-resolution MS (such as that provided by FT-ICR-MS) is in principle not required for the classification of the biogenicity of complex organic mixtures. What would be required for this type of analysis is that organics be abundant enough to provide measurably binnable features. It may, however, be difficult for in situ measurements on ET bodies to obtain organics at the abundance necessary for these analytical techniques to be usefully applied. For example recent in situ combustion analysis of Martian samples only revealed organics at ppb levels, which were insufficient to determine their indigeneity [71], while analyses of comets suggest organics such as HCHO or HCN are present at parts per hundred or parts-per-thousand levels, respectively, relative to water [72].

**Figure 7:**
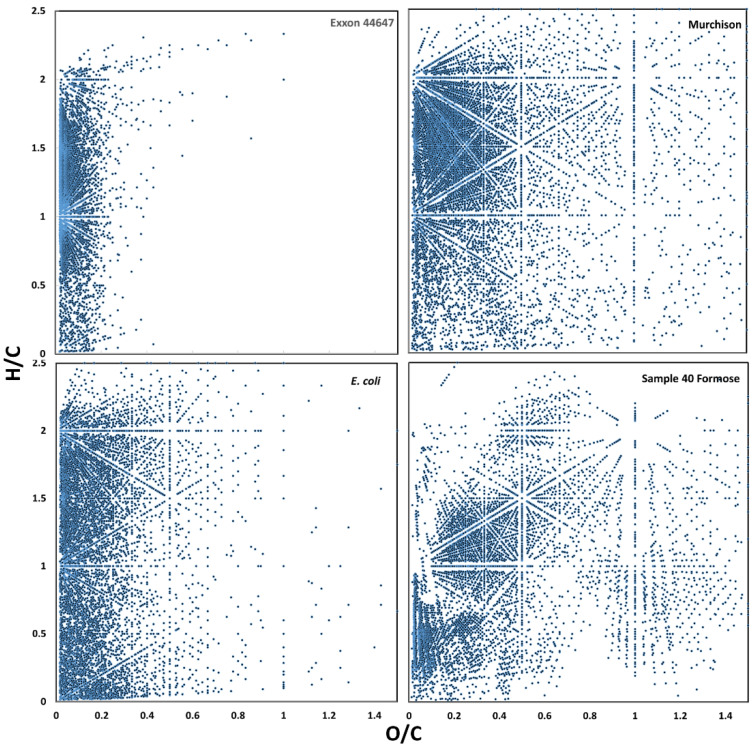
Comparative H/C vs. O/C van Krevelen plots of representative FT-ICR MS spectra showing the general tendency of aged biological and abiological samples to be depleted in heavy heteroatoms.

There are likely numerous chemical signals that contribute to the classifiability of these samples using PCA and machine learning. For example, van Krevelen diagrams of fresh and aged biological and abiological samples presented in Figure 7 show the general tendency of aged organic materials to lose heavy heteroatoms (e.g., N and O) during maturation, which would contribute to their *m*/*z* spacing heterogeneity.

The relationship between microbial chemical composition and petroleum composition is complex: a single microbial strain is not the sole source of petroleum, and it is suggestive but not beyond argument that formose-like reactions are the source of meteoritic organics, nevertheless, it is generally true that aging of heterogeneous organics results in the loss of remineralizable heteroatoms [73].

### 3.6. Possible Molecular Sources of Classification

Importantly, this methodology and these results are not based on preconceived notions of what should distinguish biological from non-biological systems, and this obviates the need for diagnostic unique mass features such as those that might derive from terrestrial biochemical “biomarker” molecules such as amino acids, lipids, or nucleotides. This may be an especially useful feature of this methodology, as the majority of modern terrestrial biomass is mainly composed of “alphabetic” polymers (i.e., nucleic acids and proteins) and specific classes of lipids [51]. A similar alphabetic principle, albeit one using other monomers, may be a common feature of extraterrestrial biology [20], e.g., it may be reasonable to assume that all biology will be based on a closed “metabolism” of organismally internally coded catalyzed reactions, though alien biology may be based on entirely different chemistry.

The majority of the dry weight composition of terrestrial organisms is macromolecular, and the majority of that material is protein and lipid [51]. Though not addressed by the machine learning methods presented above, the question of whether modern terrestrial biomonomer species are especially abundant in samples known to be of biological origin (e.g., lab-grown cells and petroleum), compared to those known not to be, was investigated. Though amino acids have long been considered promising biomarkers in searching for extraterrestrial life [74], and the use of machine learning has been applied to the discrimination of biological from abiological amino acid sets [75], they are not especially abundant in abiological samples, including carbonaceous meteorites [76], and variably well-preserved in ancient terrestrial samples [77]. Rather it is their expected overproduction by biology, chirality, and potential persistence relative to background signals that would make them good biosignatures.

Proteins are known to abiotically decay by a tail-biting mechanism, which produces cyclic dipeptides (diketopiperazines (DKPs) [78]). Over time, abiological degradation of terrestrial proteins should convert the thousands of highly statistically improbable gene-product peptides informationally produced by organisms into a few hundred statistically distributed DKPs. The information extractable from the mass distributions of such species could still be interpretatively meaningful, as even though α-amino acids are common products of abiological synthesis as evidenced by their presence in carbonaceous meteorites [76], many biologically coded amino acids are believed to have been added to the biological repertoire after the onset of coded protein synthesis [34].

**Figure 8:**
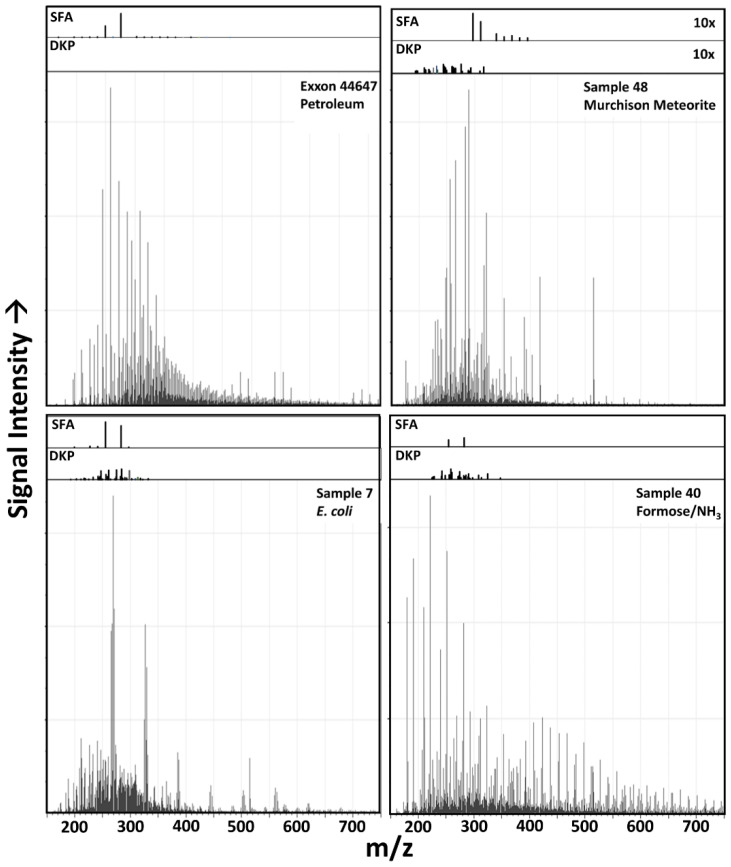
Discrete molecular formulas detected in samples using FT-ICR MS. Insets over the spectra show selected ion intensities of peaks corresponding to Saturated Fatty Acid (SFA) and Diketopiperazine (DKP)-corresponding *m*/*z* values shown to the same intensity as the main spectra. Note that the insets for the Murchison meteorite sample are enlarged 10×.

Potential mass matches for DKPs were searched for among the measured FT-ICR MS spectra (Figure 8). Of the possible 20 coded amino acid-derived DKPs, many are isobaric: the 380 possible unique sequence biogenic dipeptides would give rise to 179 unique mass DKPs (since several coded amino acids are isobaric (e.g., Leu and Ile), cyclization further reduces degeneracy (e.g., cAlaGly is identical to cGlyAla structurally), and three of these DKPs (cGlyGly, cGlyAla, cGlySer) have m/z values below the instrument detection cutoff *m*/*z* value of 150 amu. Masses corresponding to 19 of the thus 176 measurable DKP-attributable *m*/*z* values were detected in the *E. coli* samples, and many of these formulas were also detectable in the Murchison and formose samples, though the generation of the amino acids which enable these detections are often not confirmed by more detailed multidimensional analysis of abiological samples (e.g., [79]). The DKPs which have been reliably detected in CCs are only the simplest structurally possible [80], which are formed from the generally most abundant amino acids, and it is thus unlikely given this data and the known possible mass redundancy that more complex DKPs composed of amino acids which have not been detected in CCs are abundant in these samples. 

The isomeric diversity of coded amino acid DKP formulas is very large, and thus mass matches are likely even when synthetic relationships render structural assignments unlikely. For example, the formula diversity is so great in meteorite samples that 32 DKP mass matches were detected in the Murchison meteorite sample. Likewise, a formose/ammonia reaction gave mass matches for DKPs containing Trp, Tyr, Phe, Lys, Pro, Asn, Gln, Thr, Val, and Leu/Ile though most of these amino acids have not been positively detected in such reactions, even using fairly sensitive techniques [81,82]. Though it is not impossible that these species might be produced in these types of reactions, computational analysis of the isomer space of the coded amino acids suggests stable organic compounds with corresponding formulas to these species may number in the thousands or millions, and thus detected DKP *m*/*z* matches could easily correspond to millions or trillions of non-DKP isomers [83]. This again underscores the difficulty of identifying particular molecular species without the application of multidimensional techniques, which may be impractical for remote analysis. The machine learning classification methods applied here are in contrast *agnostic* as to the identities of the mass peaks detected, and rely solely on relational properties among *m*/*z* values and intensities, and thus may side-step these problems.

While proteins are “alphabetic” and thus relatively “digital” in nature despite the importance of folding processes [84], lipids are relatively more “analog” in their function, and are exceedingly diverse in modern cells [70]. The differential survivability of these two types of compound classes, among others, likely contributes to the ability of aged biological and abiological organic material to be distinguished using the methods explored here. *m*/*z* matches for straight chain alkanoic acids were thus also examined in representative samples of fresh and aged biological and abiological samples (Figure 8).

Relatively abundant C16 and C18 SFAs-corresponding *m*/*z* value peaks are common in many samples, including microbial, petroleum and laboratory simulant samples (Figure 8), while the Murchison meteorite sample shows a more extended distribution of SFA series-consistent m/z peaks, with a declining abundance with increasing *m*/*z* value. Species with these formulas are common biological products, and their anomalous abundance in abiological samples is normally suggestive of terrestrial biological contamination. This kind of contamination is a common problem in laboratory studies but should be obviated by in situ extraterrestrial sample collection and analysis. In any event, the abundance of contaminants at low levels (nor likely at high levels, since the algorithm is relational) does not appear to foil the classifier (see Figure 5), nor does the relative abundance of isobaric species which are likely incorrectly assigned, it is rather the overall relational abundance of peaks which governs classification since these individual assignments are such minor contributors to the overall MS signal. 

### 3.7. Potential Application to Detection of Ancient Terrestrial Biomarkers

While not directly examined here, determining the biogenicity of organic features in ancient terrestrial samples is difficult due to the alteration of diagnostic signals in such samples and the problem of the infiltration of more recent signals. Both of these problems are partially addressed in Figure 7 and Figure 8 of our manuscript. Various methods have been developed to address these problems, including racemization age dating (see for example [85]) and carbon isotopic analysis (see for example [86]). The petroleum samples measured here are not anywhere near as old as the samples disputed as biogenic evidence for the earliest life on Earth. It would be instructive to examine considerably older terrestrial samples using our methods to see older materials are classified. Given enough time and geologic processing, it may be that almost all measurable signals potentially diagnostic as biological, including enantiosignatures, specific compound abundances, isotopic ratios, and relational mass distributions, may converge on compositions which are inherently difficult or impossible to interpret as biogenic. Determining what measurable organic signals can be biosignatures in ancient samples is thus a deep problem. We hope this analysis may provide some new insight to it, but acknowledge that previous work may have already set boundaries for discrimination which are insurmountable.

## 4. Conclusions

The simple methods employed here correctly categorize the biogenicity of various types of complex chemical mixtures using relational mass distribution patterns from directly acquired data. Why they are able to do this is worthy of deeper scrutiny, we are currently performing more detailed analyses to attempt to identify underlying spectral features which contribute to this classifiability. It is also a fair criticism of this work that the analyzed samples are relatively few in number and already somewhat self-similar. This is an easily addressable problem through the measurement of more samples. However, it may be difficult to find more diverse and disparate abiological simulations of prebiotic chemistry: only so many chemistries are likely highly diversity generating, since such processes depend on the products of reactions still being mechanistically and energetically favorable for further reactions to proceed.

There is some redundancy in the means of generation of the types of abiological samples studied here (as they start from common precursors such as aldehydes, ammonia, and HCN, which may be reflective of actual synthetic processes), and petroleum and meteoritic organics are self-similar to some degree in terms of the nature of their geochemical processing, which may be difficult to precisely reproduce in the laboratory [87]. These various nuances of processing (e.g., temperature, pH, mineralogical context) could cause complex organic mixtures to evolve in unique ways, possibly generating mixtures differing in ways that could confound the classification methods presented here. This is an open challenge, and we welcome the presentation of samples which may contradict our conclusions.

Where the maximum amount of discriminating information lies with respect to *m*/*z* in these samples as addressed by this technique is an important question. For example, we somewhat arbitrarily limited our study to a mass window (*m*/*z* 150–750) we could easily measure where signal is abundant. Some sub-section of this mass window could provide the greatest discrimination among classes. Such information could be used to design future spacecraft-based instrumentation so as to be able to extract the greatest amount of information from remotely analyzed samples. We did not conduct such studies here, but this is an attractive problem for future work. We note also that existing organic analyses conducted on organics detected in situ from both Mars [11] and Enceladus [12] may already be of sufficient mass resolution for this type of categorization using these techniques.

Abiological processes can produce extreme chemical heterogeneity, as can biological processes. However, biological systems, which are highly entailed due to their relational generative processes, evidently steer products in unique directions relative to abiological processes. Though the products of both of these types of processes may degrade by abiological environmental processes in both common and unique ways, the signals of these processes may still be easily differentiated when the signals are abundant. Thus, biological entailment may be detectable via the mass distribution of organic compounds. This kind of entailment may also be detectable in laboratory simulations of the origins of life [88].

It is thus promising that simple mass spectrometry of even moderate mass resolution, using instruments which can be miniaturized for spacecraft-based analysis, may be sufficient for the detection of evidence of extraterrestrial biology. Such technological capabilities are likely already available for remote spacecraft-based instrumentation, though they could likely be fine-tuned, and such detection is therefore presently more limited by data-processing methods and the availability of data allowing this kind of classification.

## Data Availability

Raw data files are available for all data presented here upon request from the corresponding author.

## Supplementary Materials

The following are available online at https://www.mdpi.com/2075-1729/11/3/234/s1, Figures S1A–S27B, mass spectra and associated Kendrick plots.
