# Erratum: Guttenberg et al. Classification of the Biogenicity of Complex Organic Mixtures for the Detection of Extraterrestrial Life. *Life* 2021, *11*, 234

**DOI:** 10.3390/life11060461

**Published:** 2021-05-21

**Authors:** Nicholas Guttenberg, Huan Chen, Tomohiro Mochizuki, H. James Cleaves

**Affiliations:** 1 Earth-Life Science Institute, Tokyo Institute of Technology, Ookayama, Tokyo 152-8550, Japan; ngutten@gmail.com (N.G.); tomo.mochiviridae@elsi.jp (T.M.); 2 Cross Labs, Cross Compass Ltd., 2-9-11-9F Shinkawa, Chuo-ku, Tokyo 104-0033, Japan; 3 GoodAI, Na Petynce 213/23b, 169 00 Prague, Czech Republic; 4 National High Magnetic Field Laboratory, Florida State University, 1800 East Paul Dirac Drive, Tallahassee, FL 32310-4005, USA; huan.chen@magnet.fsu.edu; 5 Institute for Advanced Study, 1 Einstein Drive, Princeton, NJ 08540, USA; 6 Blue Marble Space Institute of Science, Seattle, WA 98104, USA

There was an error in the original article [1]. It has come to our attention that there has been previous work presented in print concerning some of the topics addressed in this article. The abstract has thus been modified and additional references citing this work have been added.

A correction has been made to the ***Abstract***:

The sentence “To our knowledge this is the first comprehensive demonstration of the utility of this analytical technique for the detection of biology.” has been deleted.

A correction has been made to ***Introduction*, *Paragraph 7***:

The sentence “Individual signatures are generally not conclusive, but integrated evidence across multiple independent signatures can potentially sharpen detection [4].” has been added.

A correction has been made to ***Introduction*, *Paragraph 8***:

The sentence “However, Mathis et al. [5] have proposed a method based on assembly pathways to identify molecules which, regardless of the apparent complexity of their structure, would be fundamentally complex for a system to produce. This may provide a way to identify molecules produced by a form of life unlike that on Earth, which are yet likely to be the product of a living system.” has been added.

The corresponding references have been newly added:

4. Walker, S.I.; Cronin, L.; Drew, A.; Domagal-Goldman, S.; Fisher, T.; Line, M.; Millsaps, C. Probabilistic frameworks for life detection. In *Planetary Astrobiology (Space Science Series)*; Meadows, V., Des Marais, D.J., Arney, G., Schmidt, B., Eds.; University of Arizona Press: Phoenix, AZ, USA, 2020; pp. 447–505.
5. Mathis, C.; Carrick, E.; Keenan, G.; Cooper, G.; Graham, H.; Bame, J.; Craven, M.; Bell N, Gromski, P.S.; Swart, M.; et al. Identifying molecules as biosignatures with assembly theory and mass spectrometry. *Chemarxiv* **2020**, preprint, doi:10.26434/chemrxiv.13227881.

The authors apologize for any inconvenience caused and state that the scientific conclusions are unaffected. The original article has been updated.
