# Supplementary material for: Classification of the Biogenicity of Complex Organic Mixtures for the Detection of Extraterrestrial Life
Source: Life (Basel). 2021 Mar 12;11(3):234. doi: 10.3390/life11030234 (PMC8001260; doi:10.3390/life11030234)
Supplement: Supplementary file 1 [file life-11-00234-s001.pdf]

Figure S1A. Negative mode ESI FT-ICR-MS spectrum of organics extracted from cultured E. coli cells after heating in the dry state for six days.

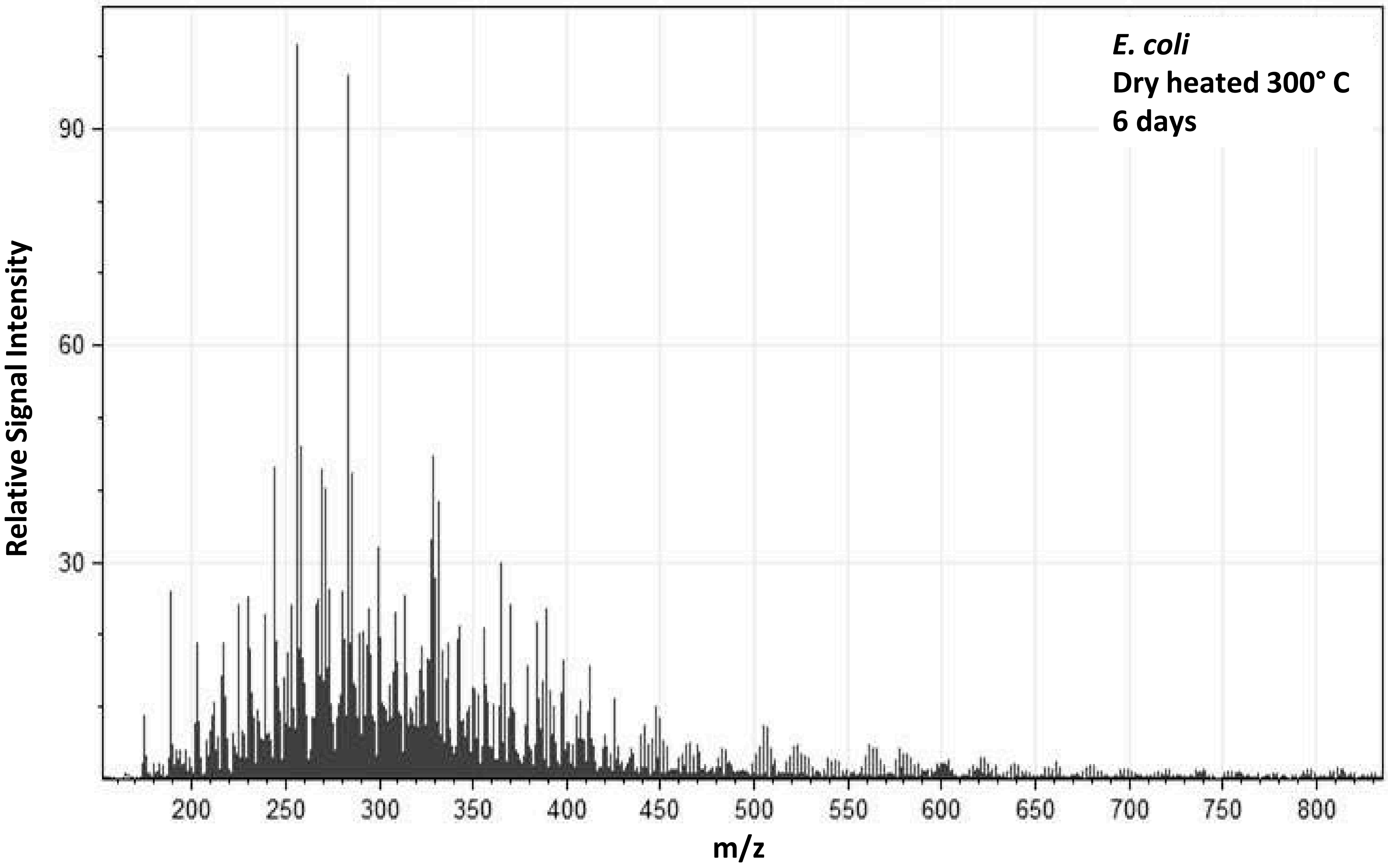

Figure S1B. Odd and even Kendrick Mass Defect plots of the organics extracted from cultured E. coli cells after heating in the dry state for six days.

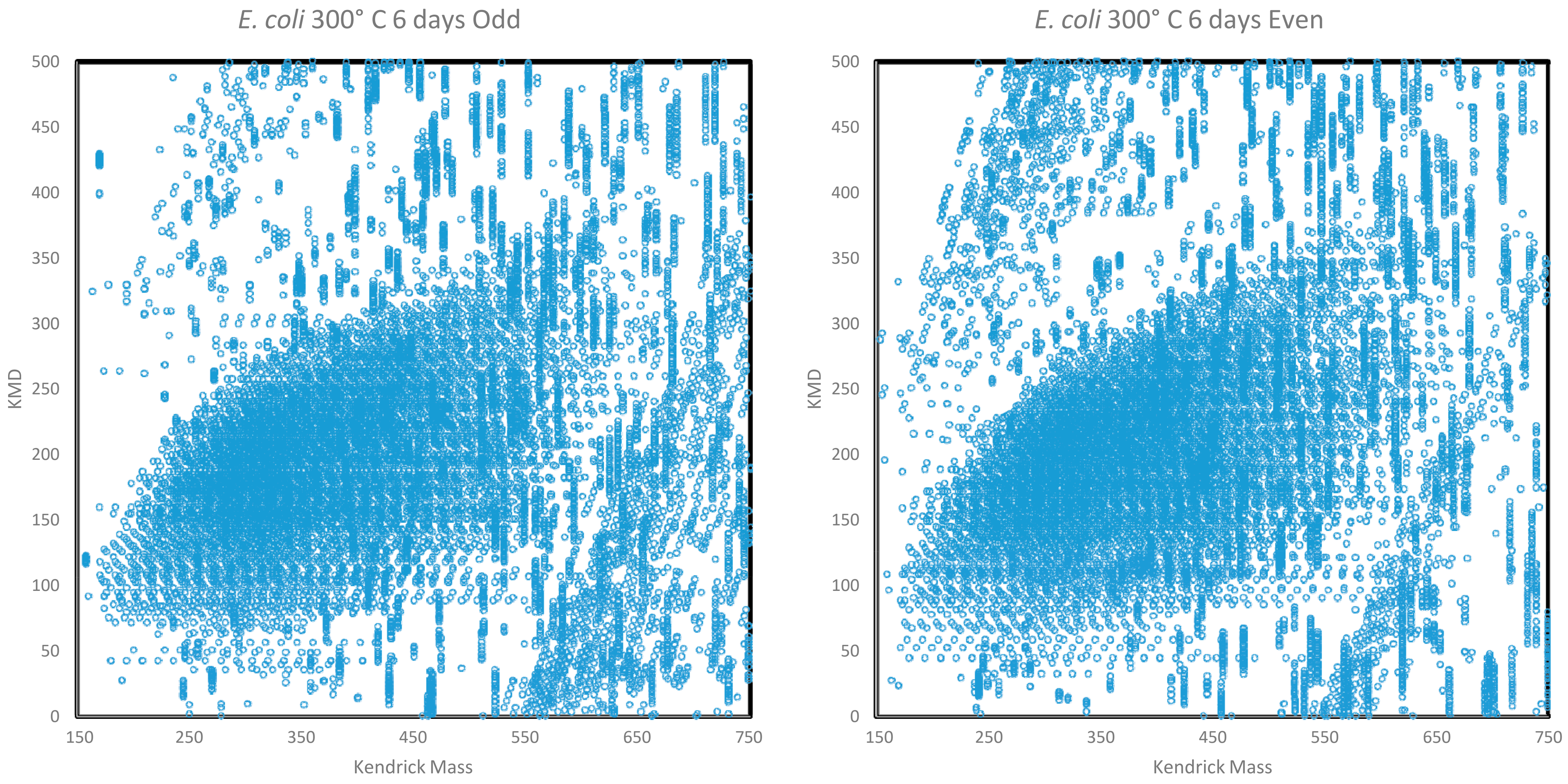

Figure S2A. Negative mode ESI FT-ICR-MS spectrum of organics extracted from cultured E. coli cells after heating in the dry state for four days.

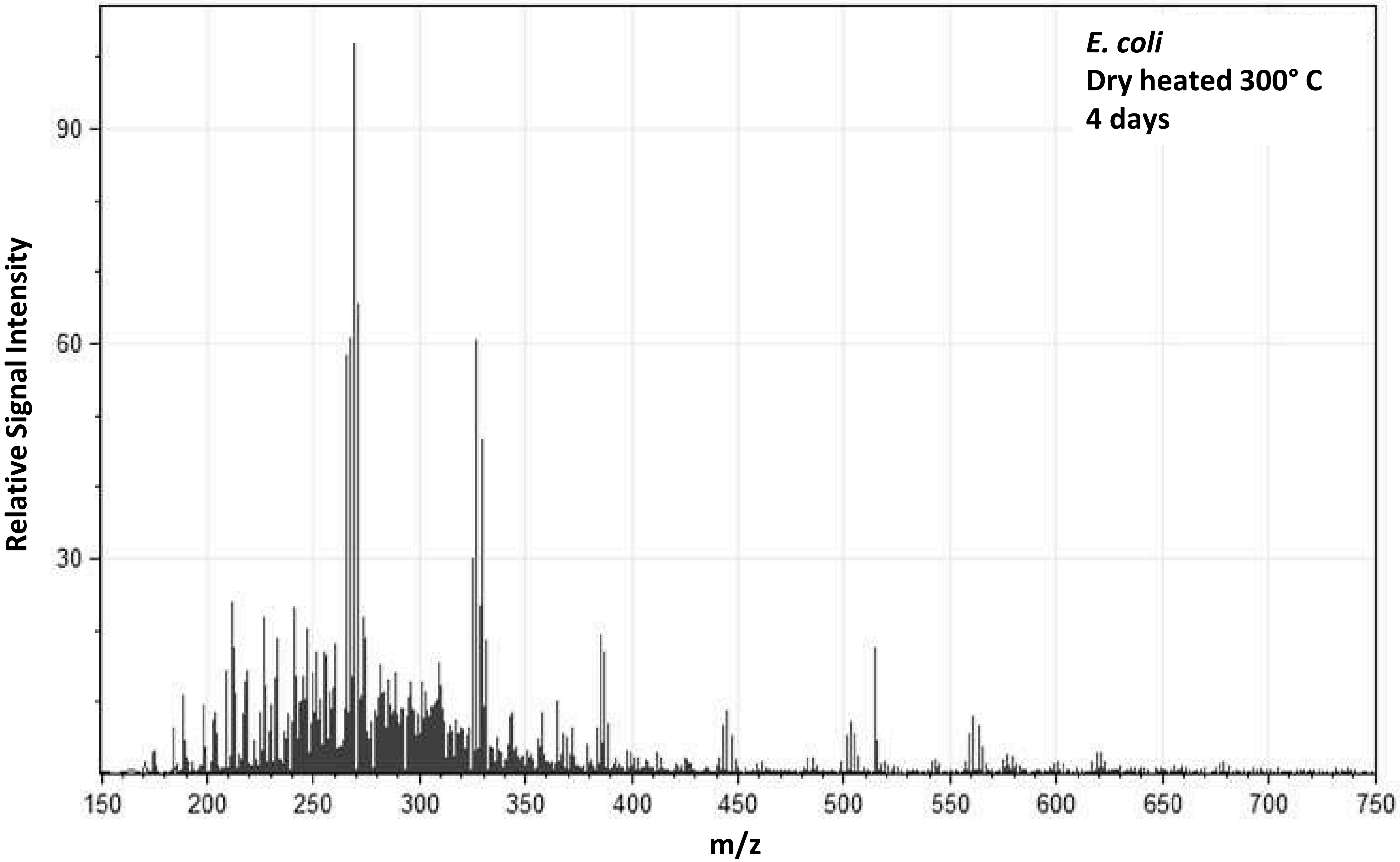

Figure S2B. Odd and even Kendrick Mass Defect plots of the organics extracted from cultured E. coli cells after heating in the dry state for four days.

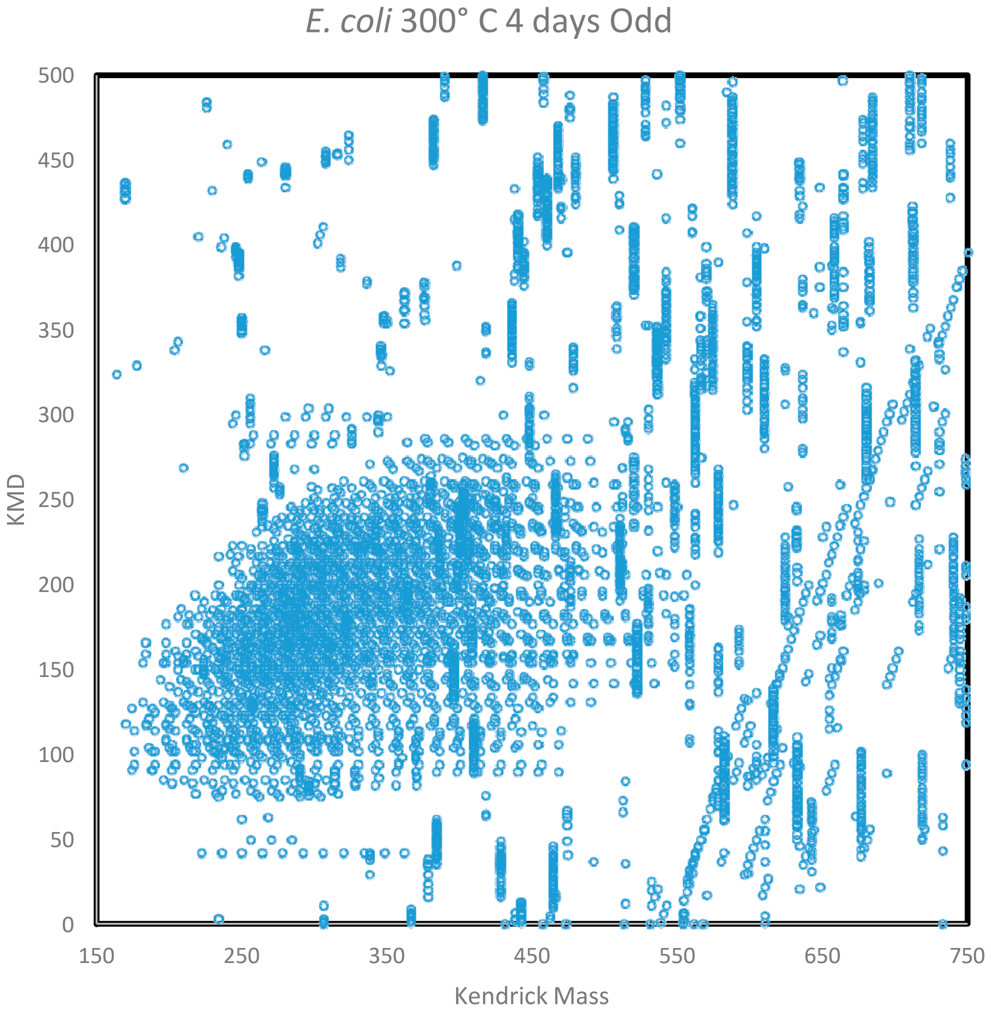

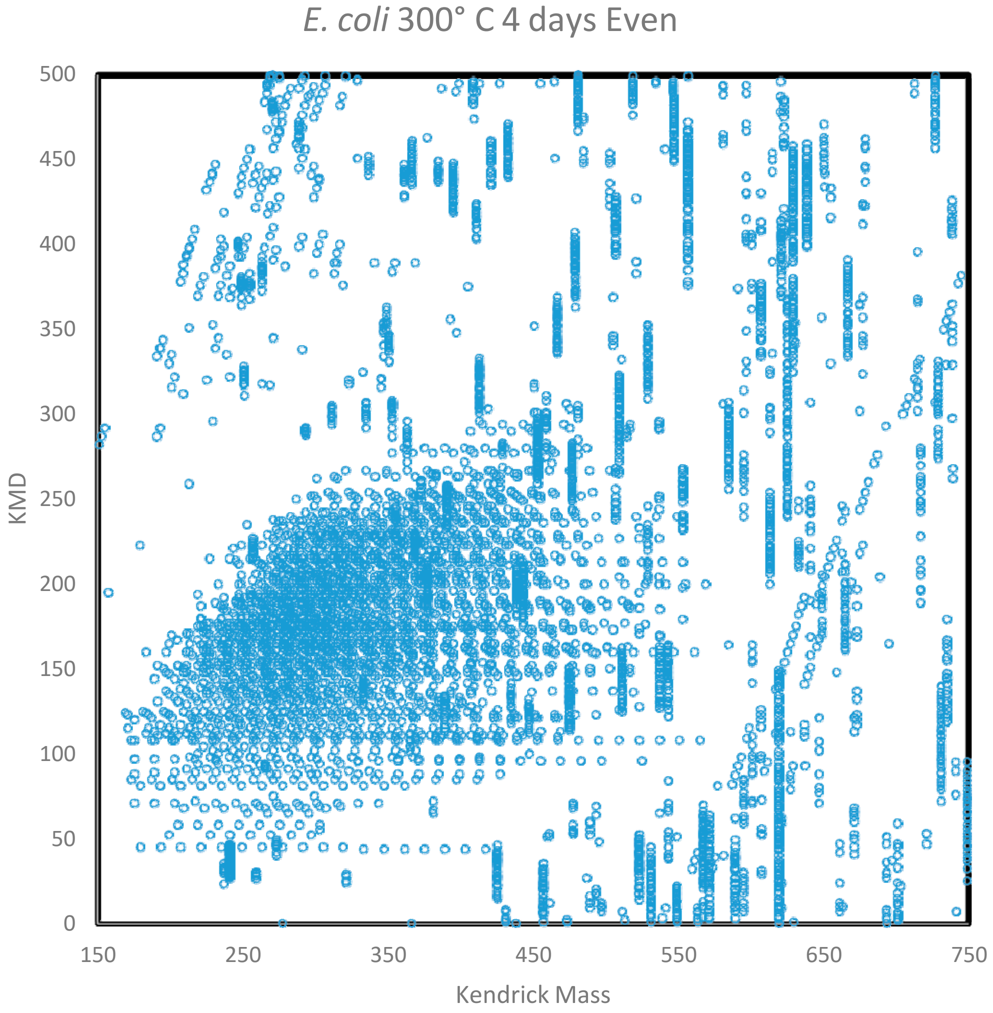

Figure S3A. Negative mode ESI FT-ICR-MS spectrum of organics extracted from the Orgueil meteorite using methanol as described in the methods section.

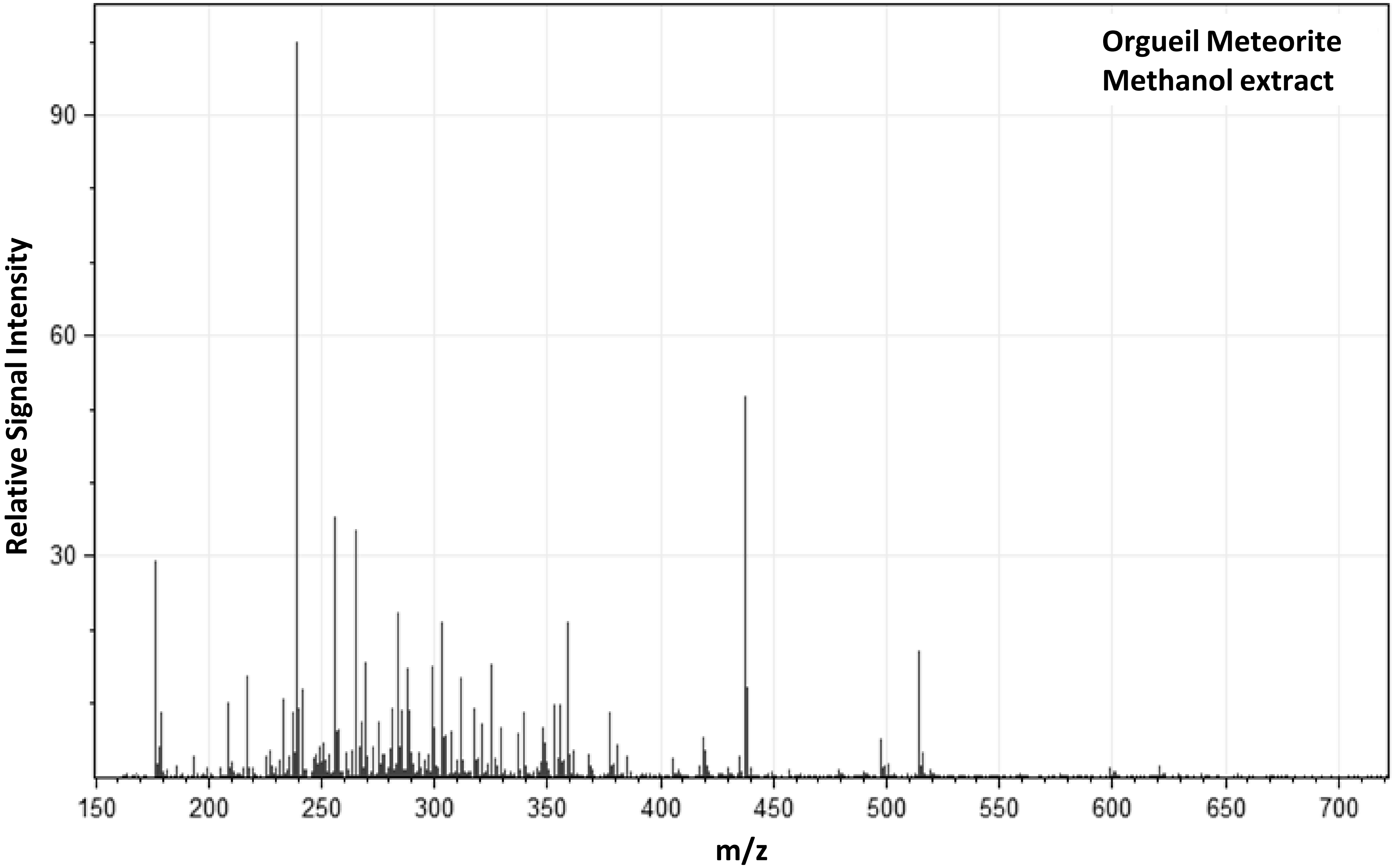

Figure S3A. Odd and even Kendrick Mass Defect plots of the organics extracted from the Orgueil meteorite using methanol as described in the methods section.

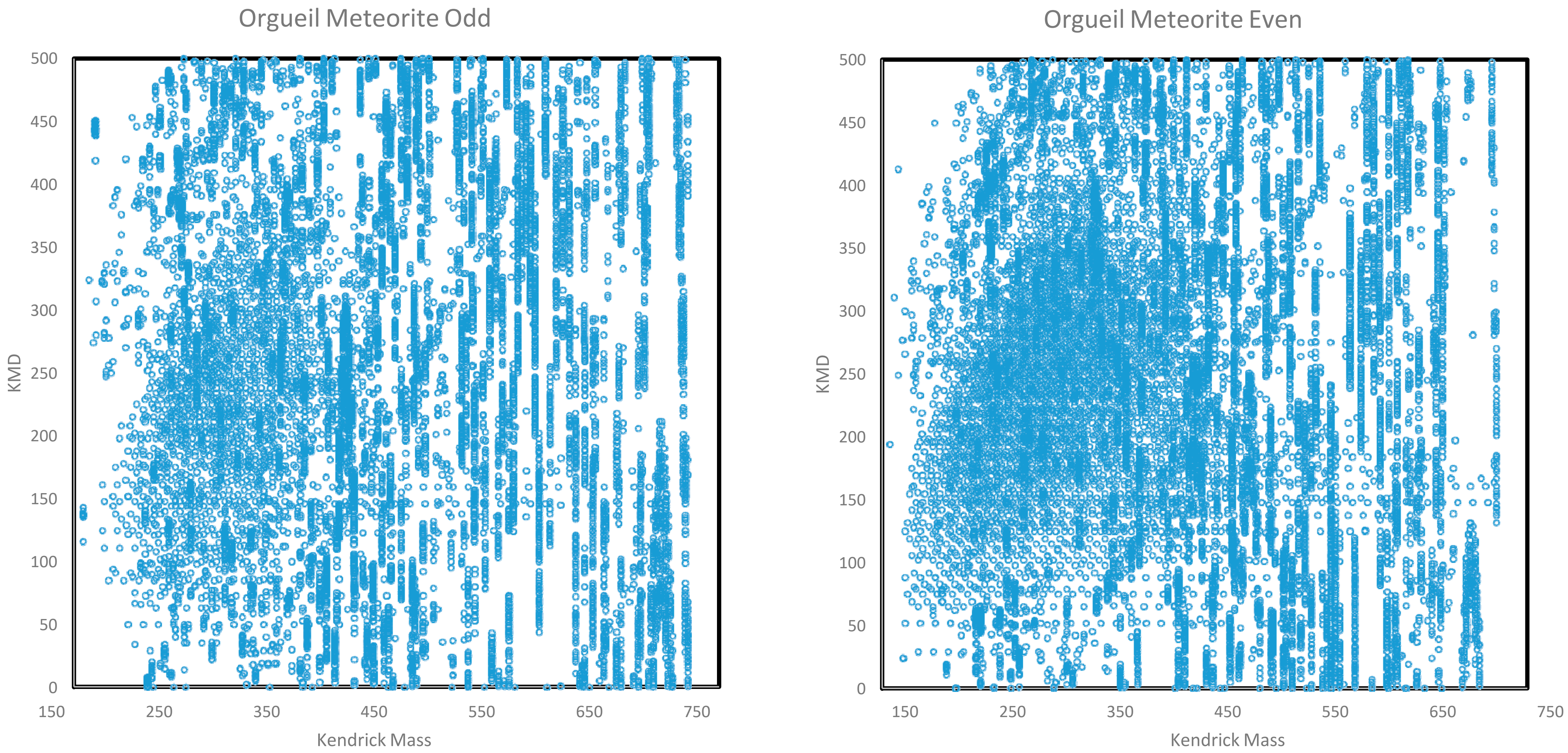

Figure S4A. Negative mode ESI FT-ICR-MS spectrum of organics extracted from the Allende meteorite using methanol as described in the methods section.

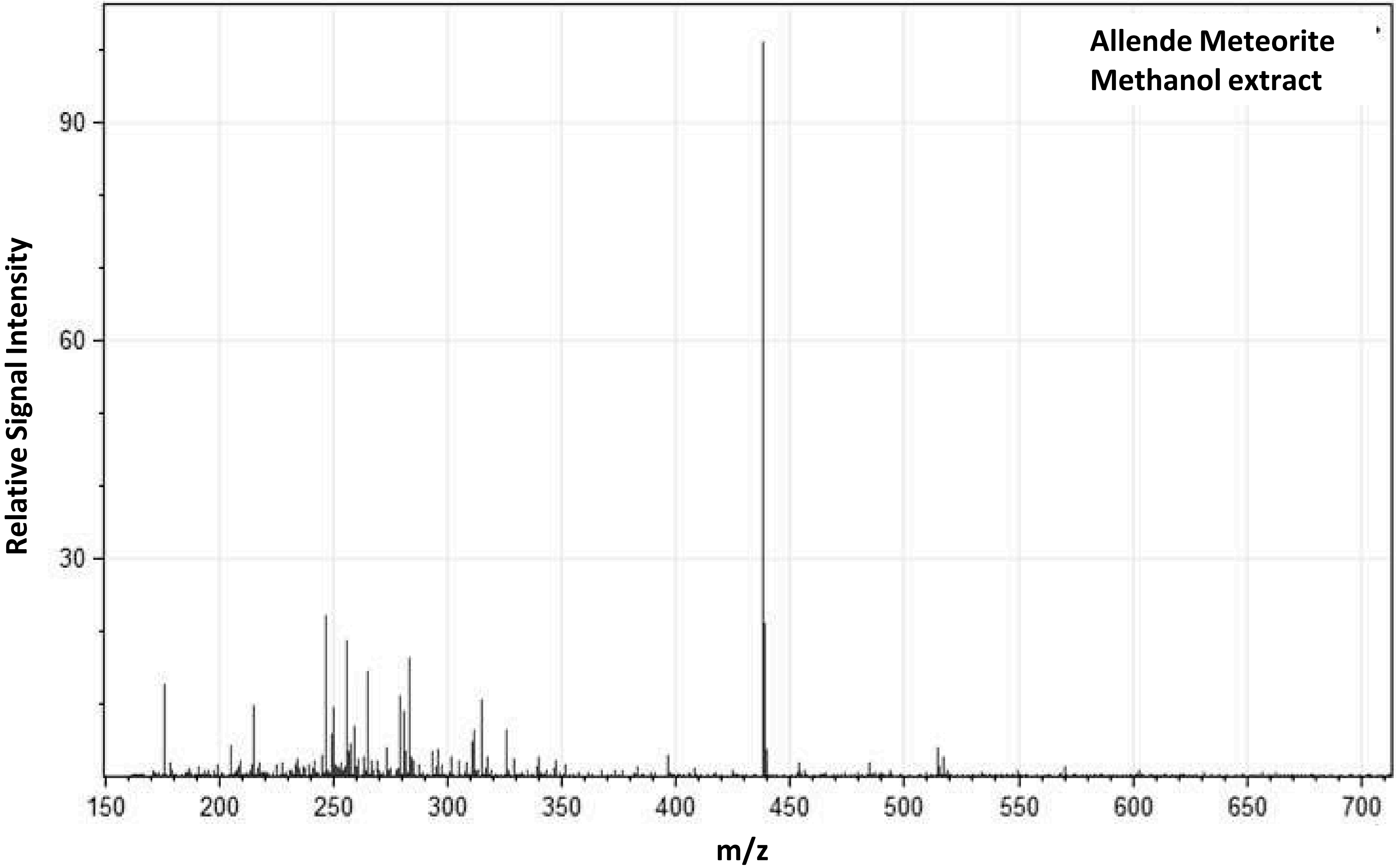

Figure S4B. Odd and even Kendrick Mass Defect plots of the organics extracted from the Allende meteorite using methanol as described in the methods section.

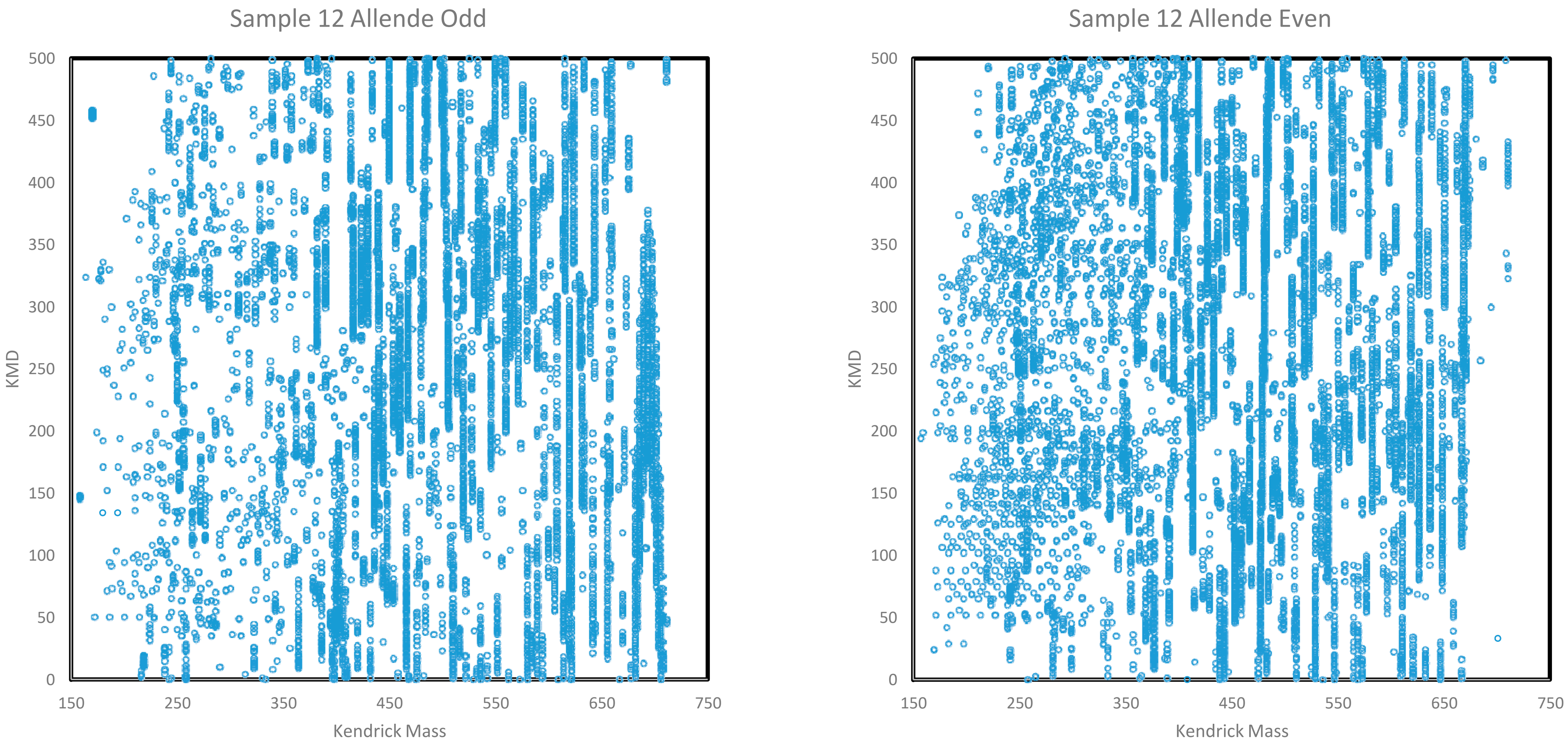

Figure S5A. Negative mode ESI FT-ICR-MS spectrum of organics extracted from the Murchison meteorite using methanol as described in the methods section.

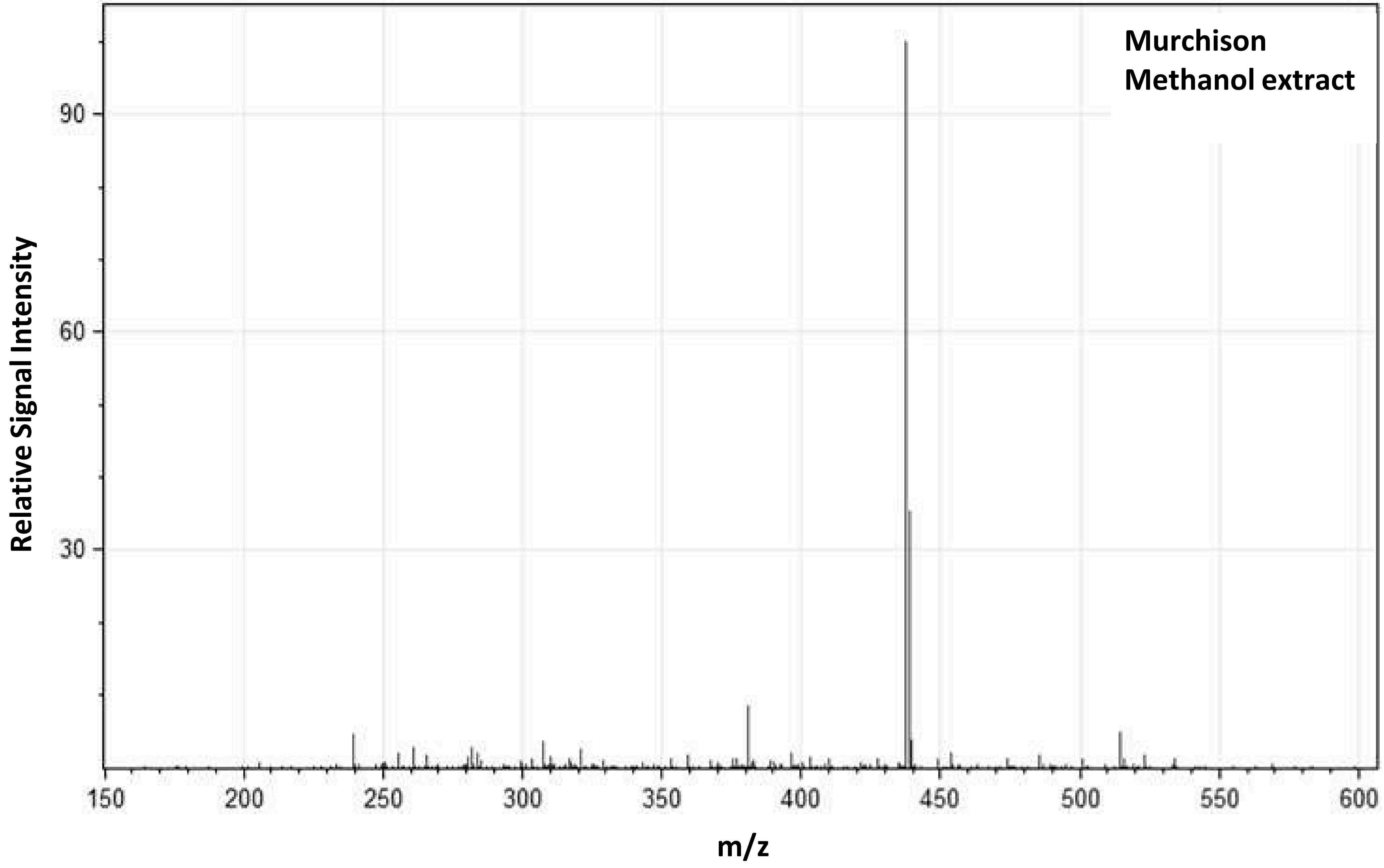

Figure S5B. Odd and even Kendrick Mass Defect plots of the organics extracted from the Murchison meteorite using methanol as described in the methods section.

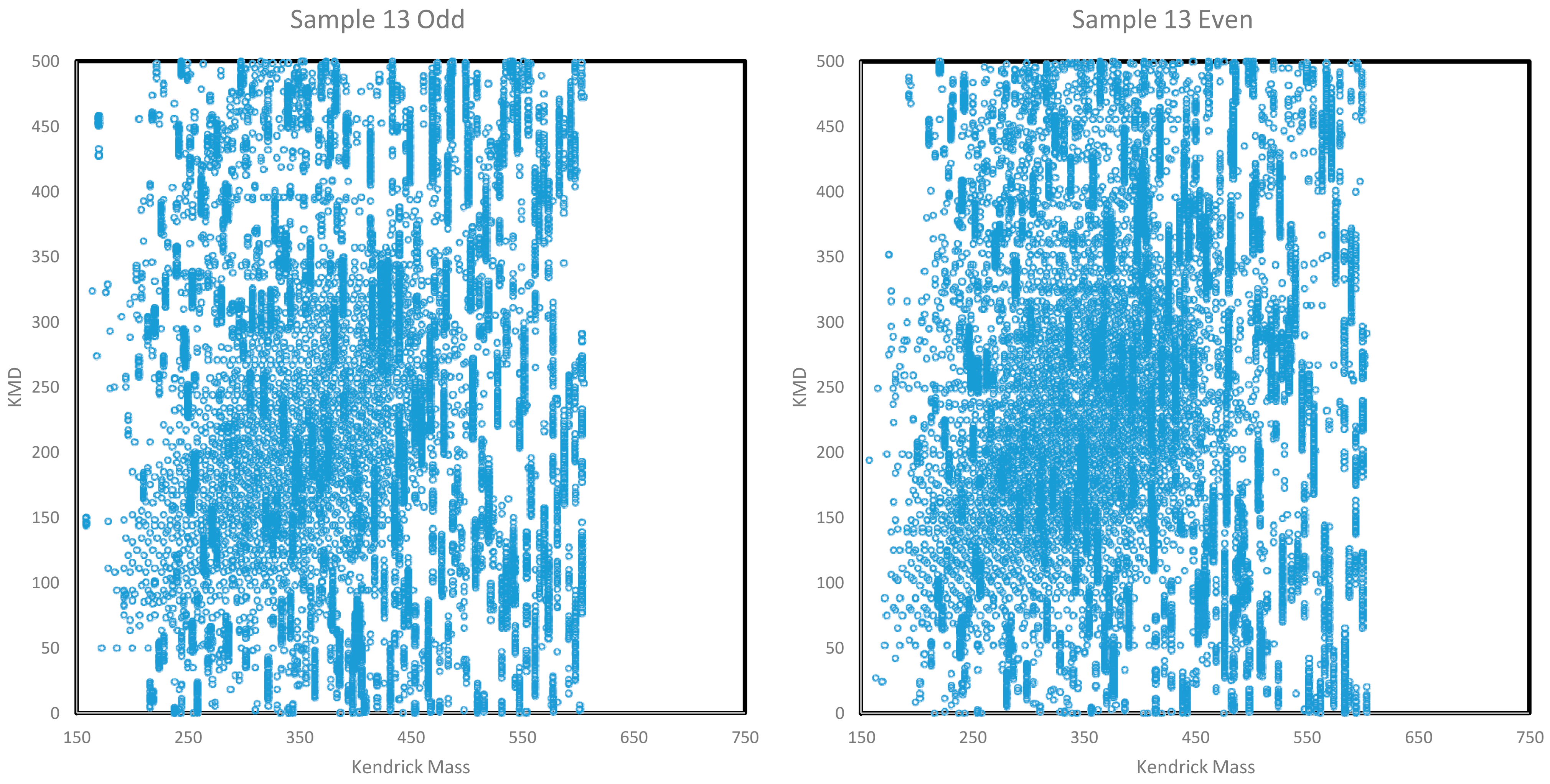

Figure S6A. Negative mode ESI FT-ICR-MS spectrum of organics extracted from cultured a marine hyperthermophilic Archeabacterium *Aeropyrum sp.*

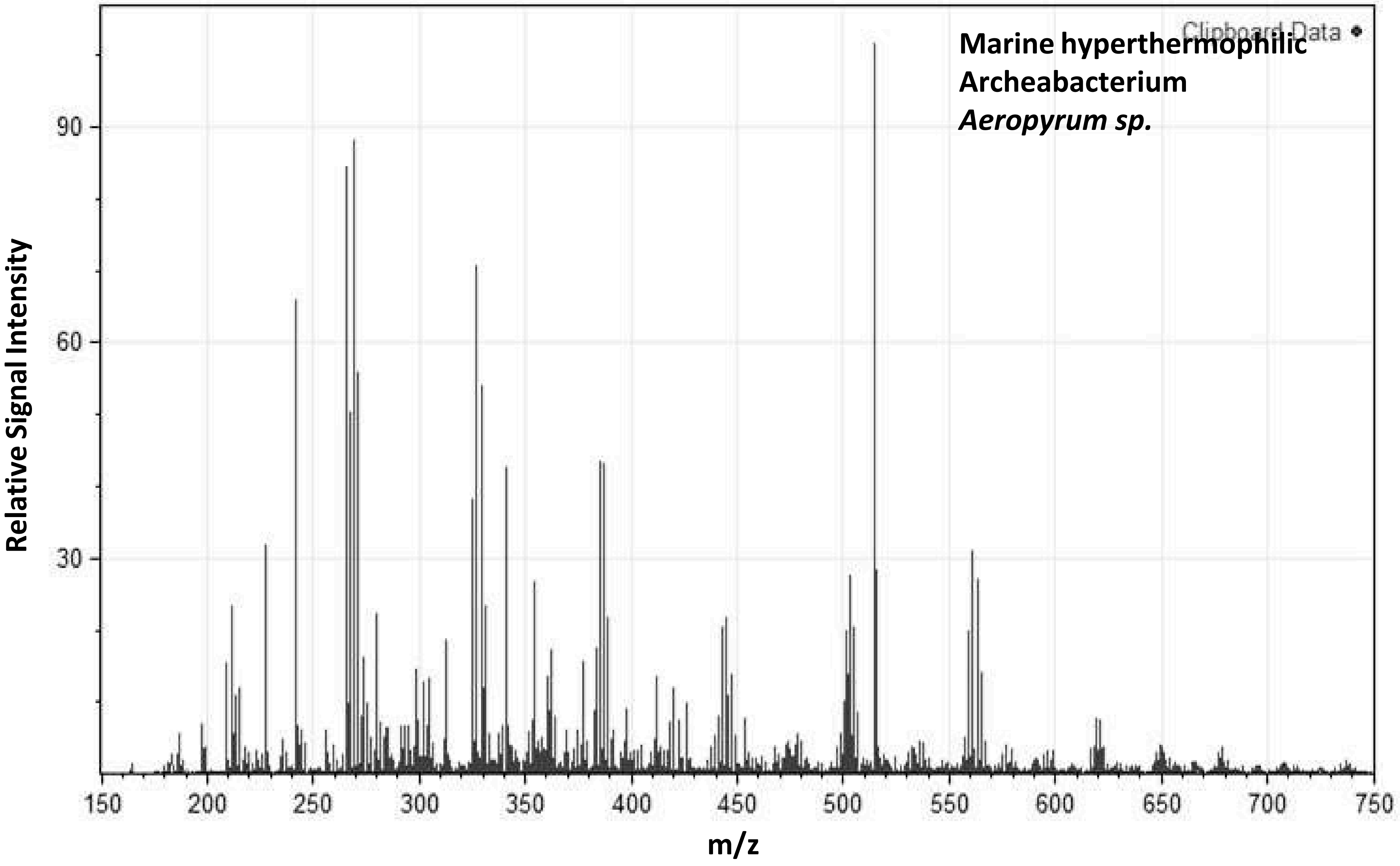

Figure S6B. Odd and even Kendrick Mass Defect plots of the organics extracted from cultured a marine hyperthermophilic Archeabacterium *Aeropyrum sp.*

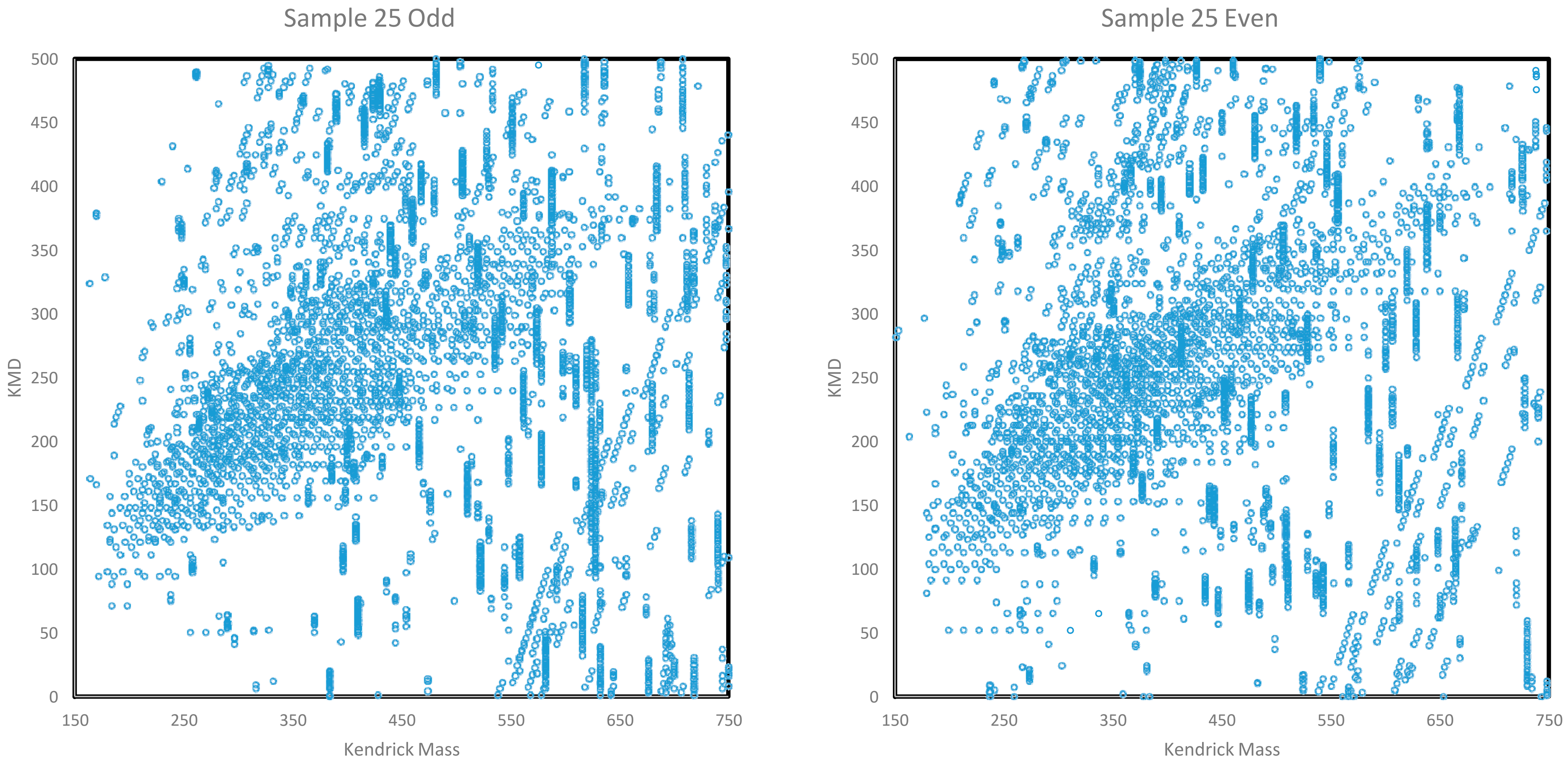

Figure S7A. Negative mode ESI FT-ICR-MS spectrum of organics extracted from cultured a marine hyperthermophilic Archeabacterium *Pyrobaculum sp.*

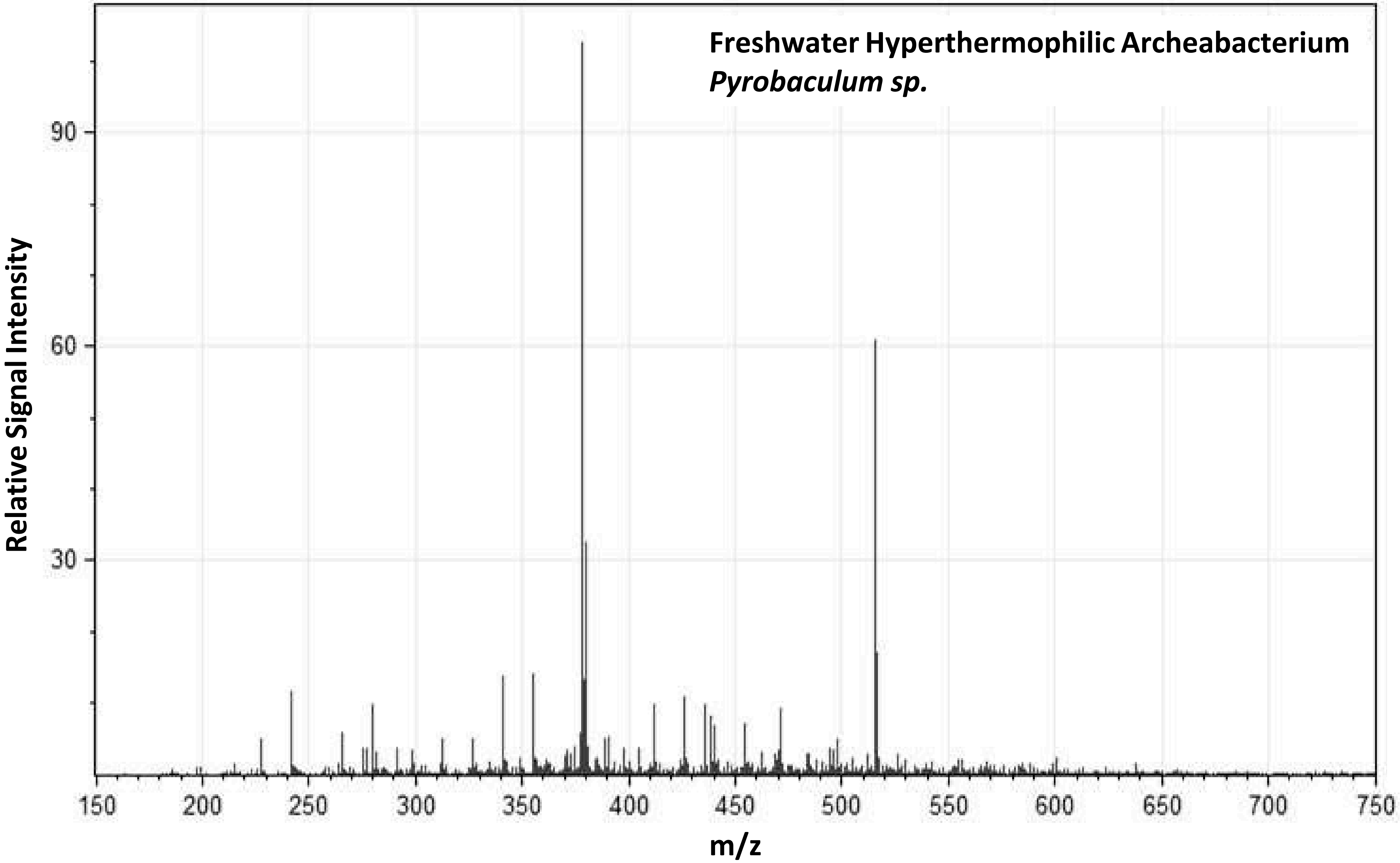

Figure S7B. Odd and even Kendrick Mass Defect plots of the organics extracted from cultured a marine hyperthermophilic Archeabacterium *Pyrobaculum sp.*

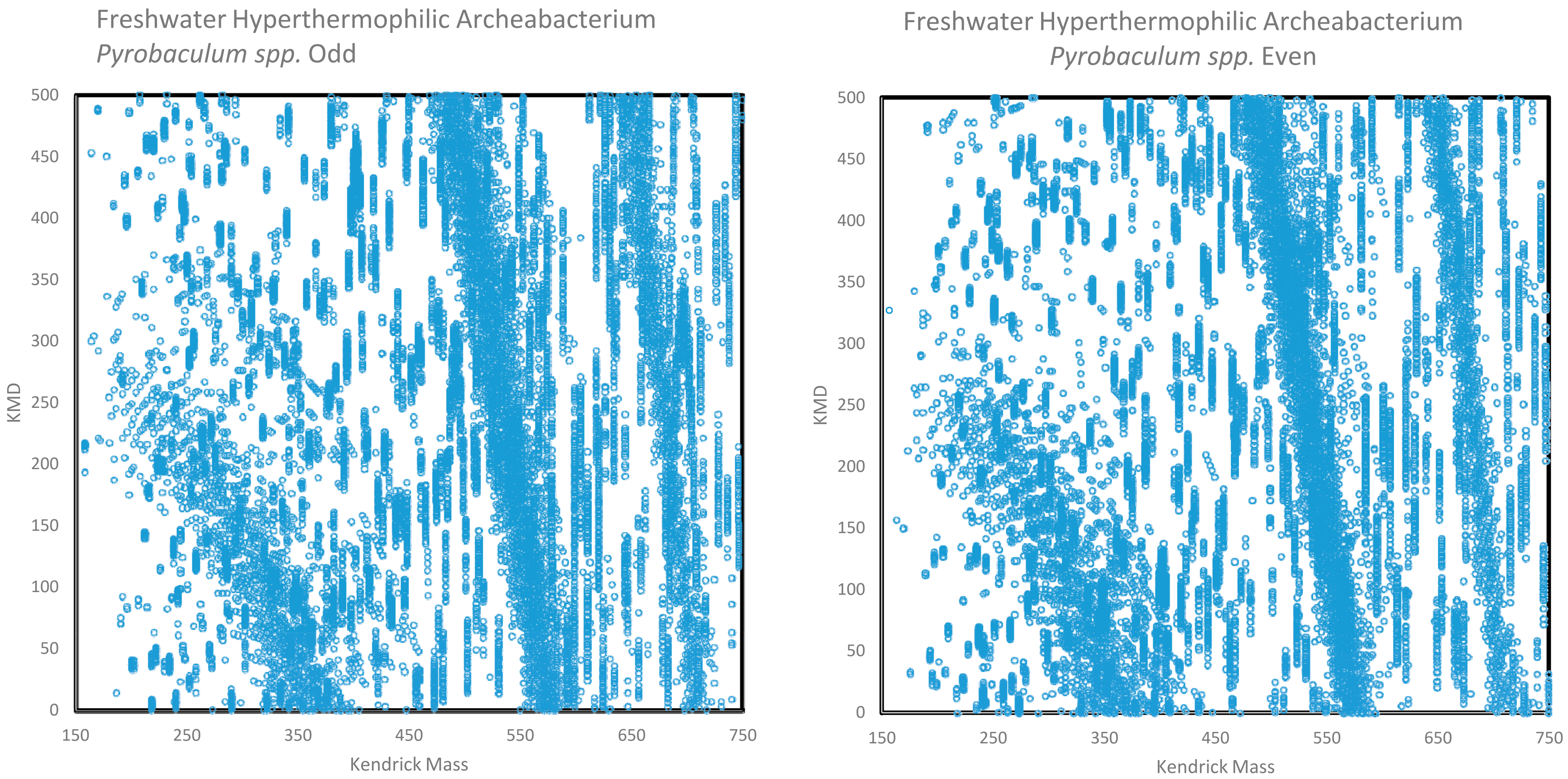

Figure S8A. Negative mode ESI FT-ICR-MS spectrum of organics extracted from cultured a marine Thermophilic Bacterium *Rhodothermus sp.*

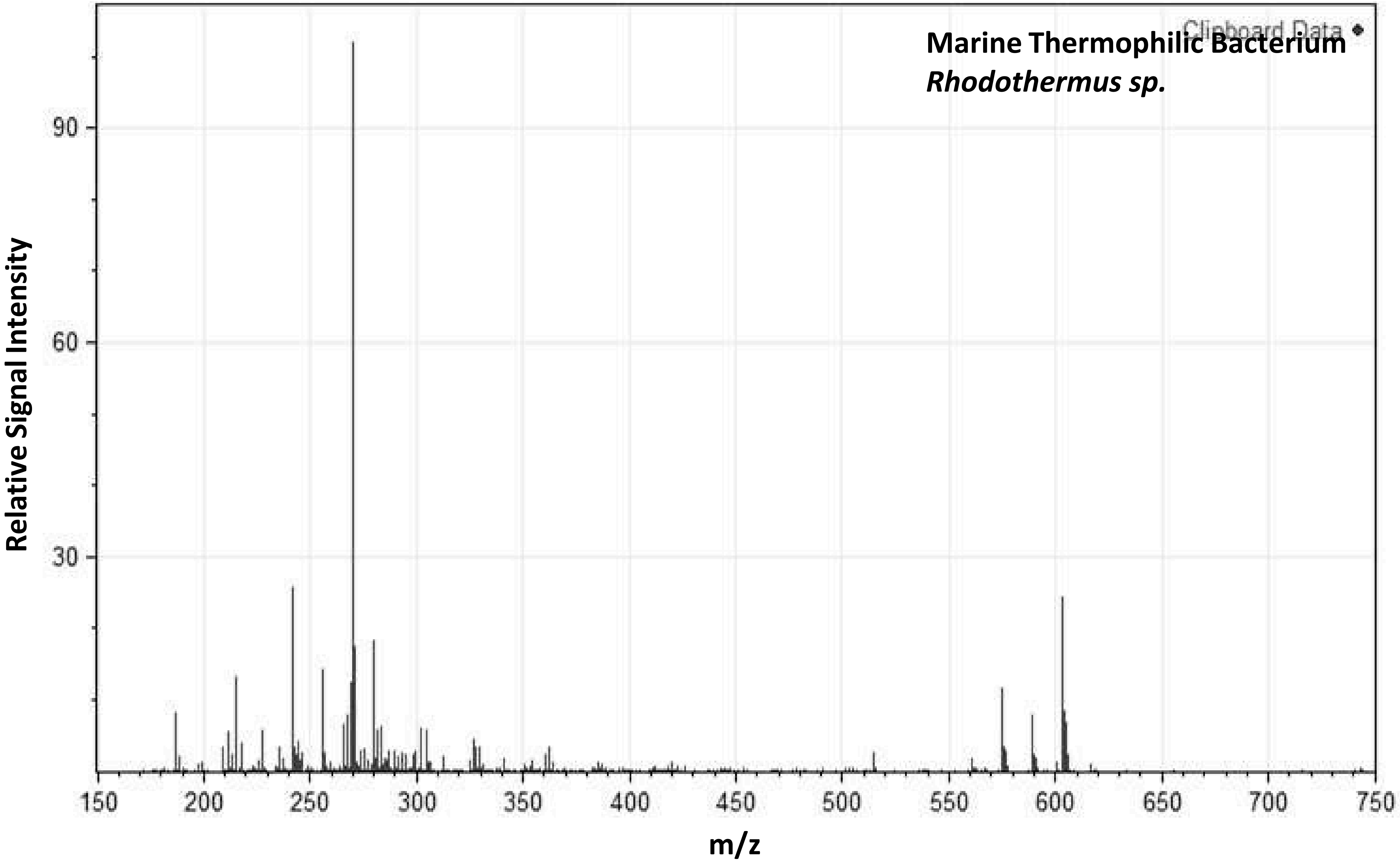

Figure S8B. Odd and even Kendrick Mass Defect plots of the organics extracted from cultured a marine thermophilic bacterium *Rhodothermus sp.*

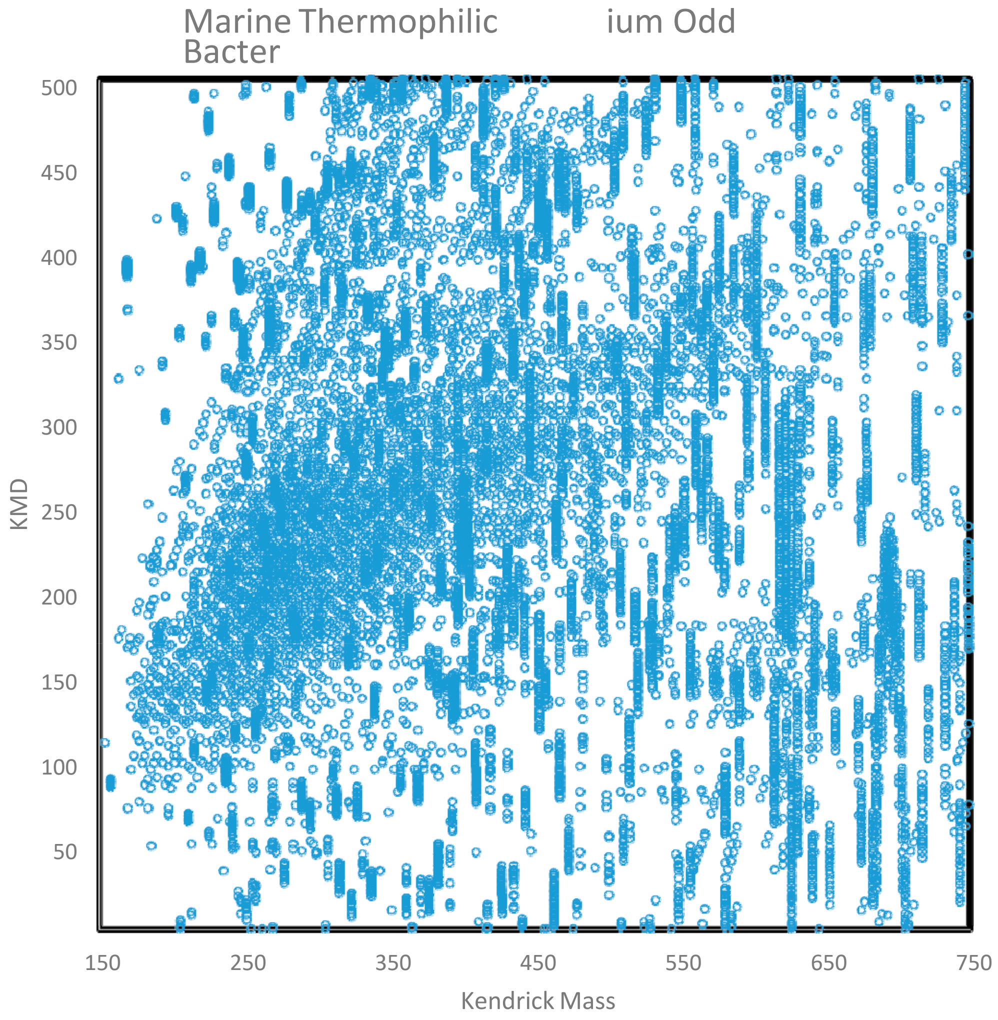

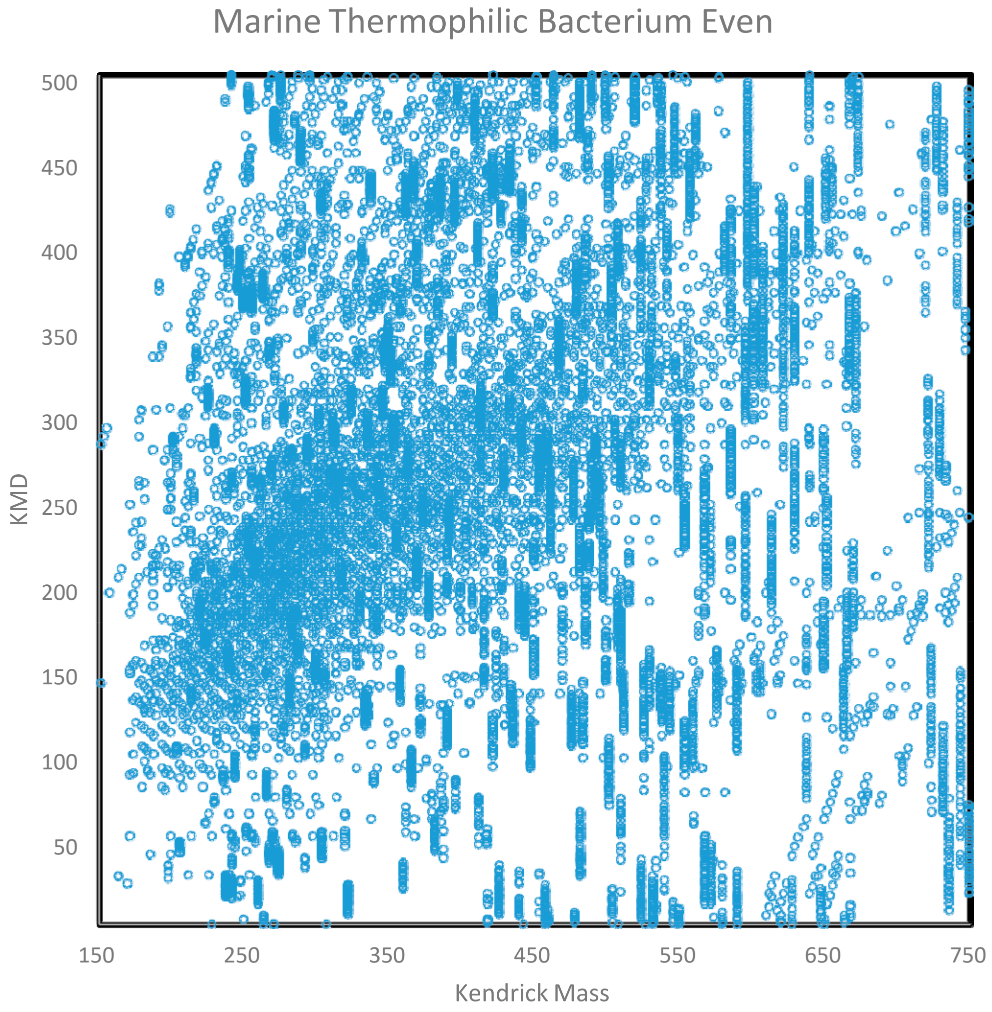

0

0

m/z

Figure S9A. Negative mode ESI FT-ICR-MS spectrum of organics extracted from Bell Creek Petroleum.

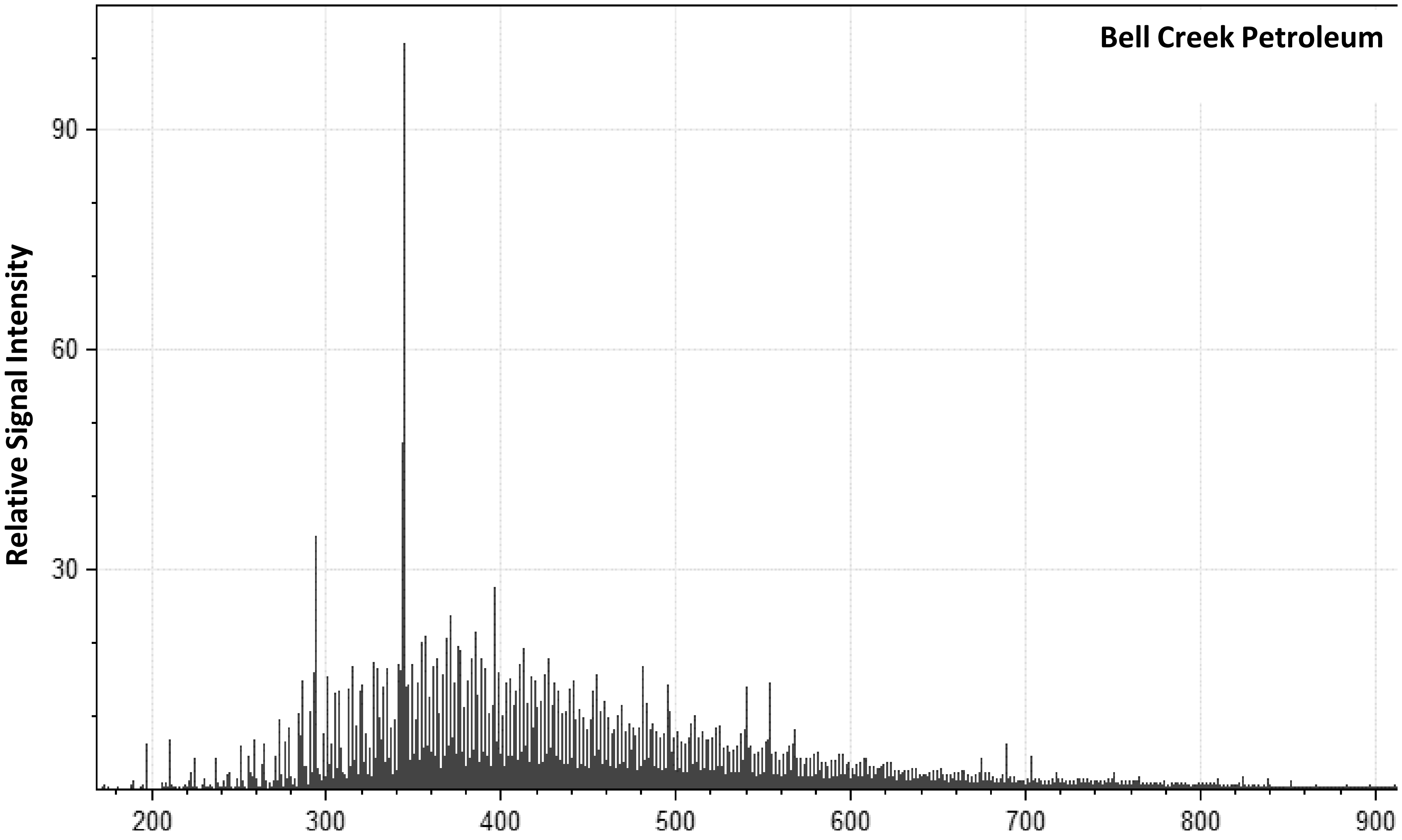

750

Figure S9B. Odd and even Kendrick Mass Defect plots of the organics extracted from Bell Creek Petroleum.

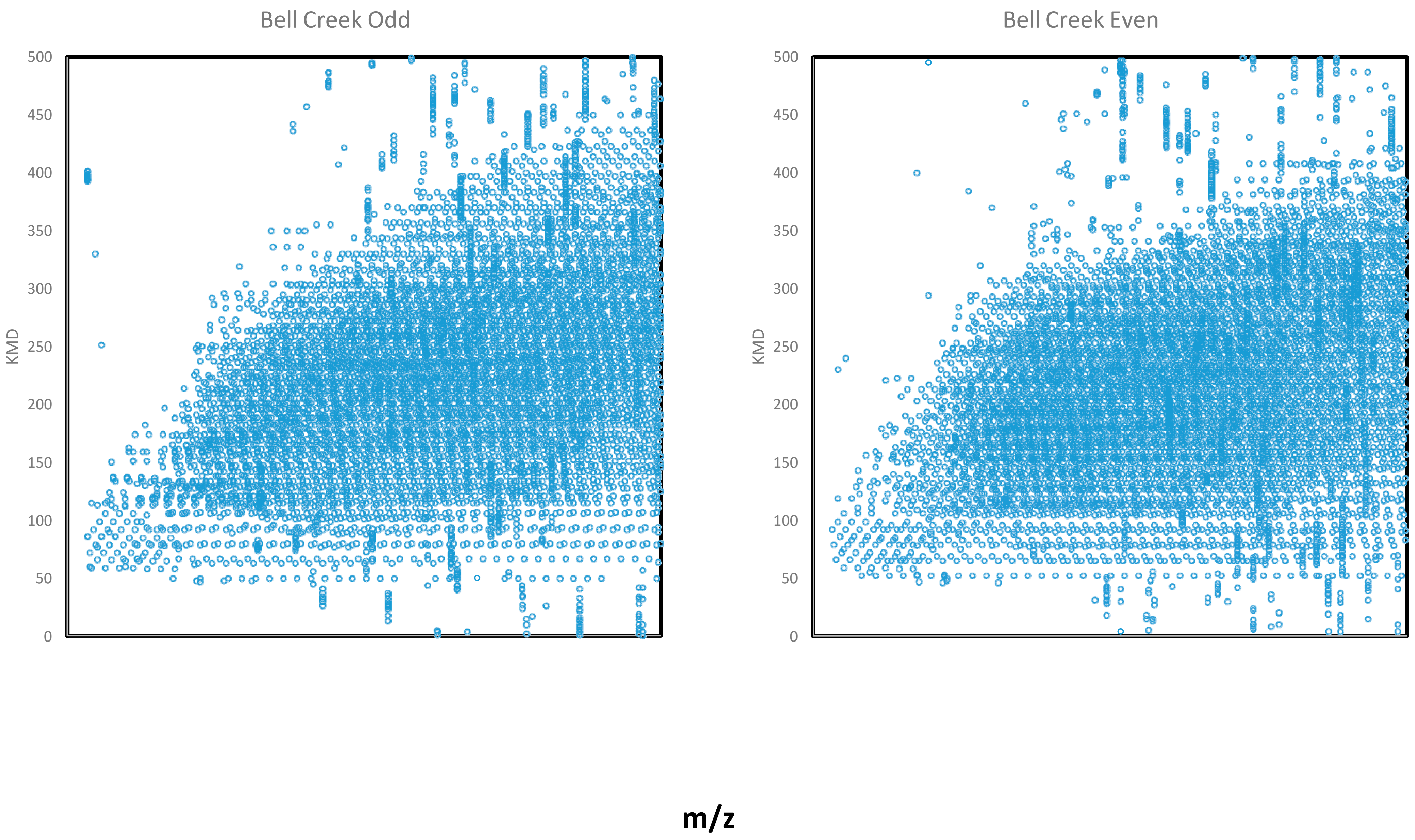

Figure S10A. Negative mode ESI FT-ICR-MS spectrum of organics extracted from Exxon 44647 Petroleum.

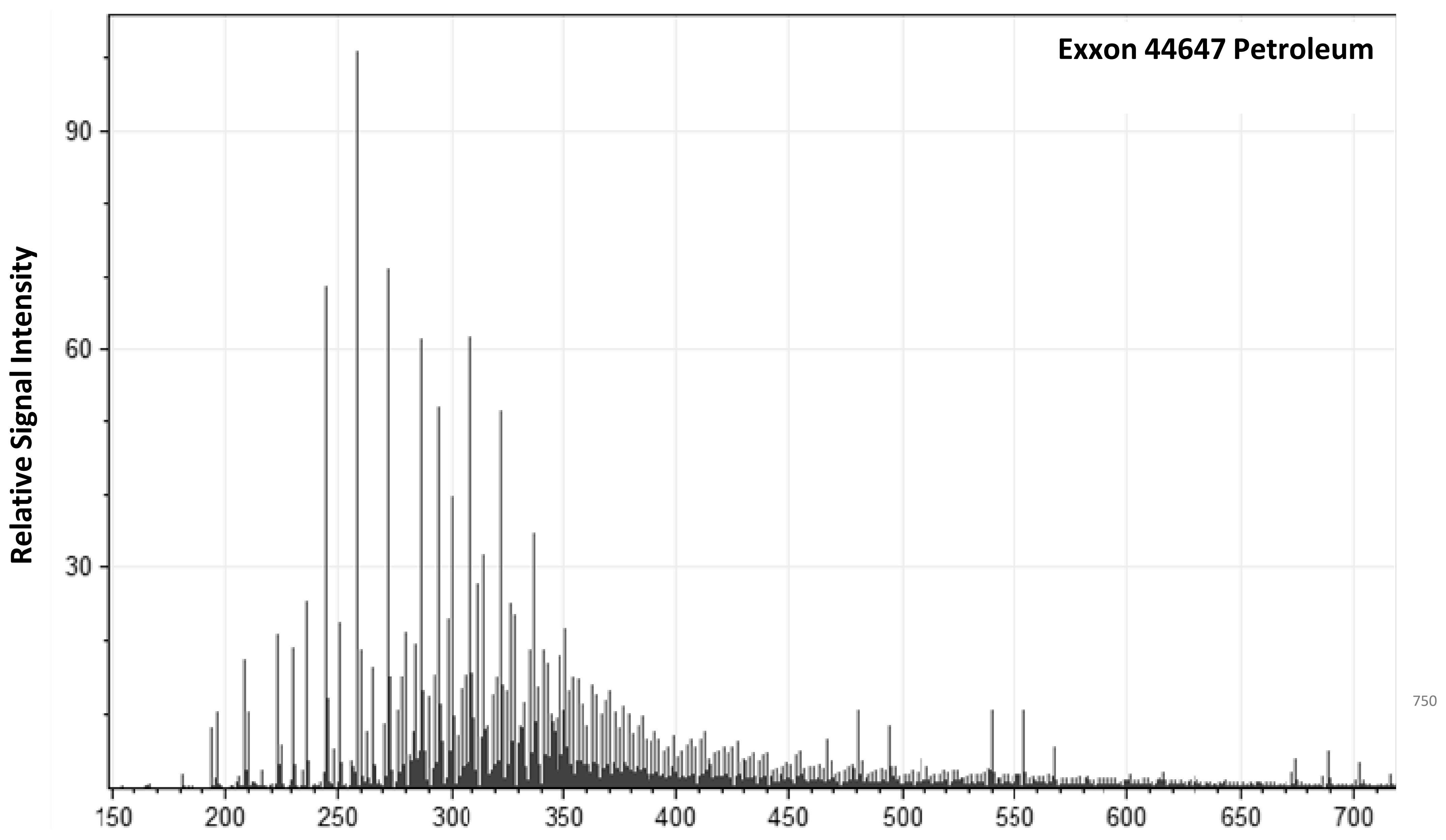

Figure S10B. Odd and even Kendrick Mass Defect plots of the organics extracted from Exxon 44647 Petroleum.

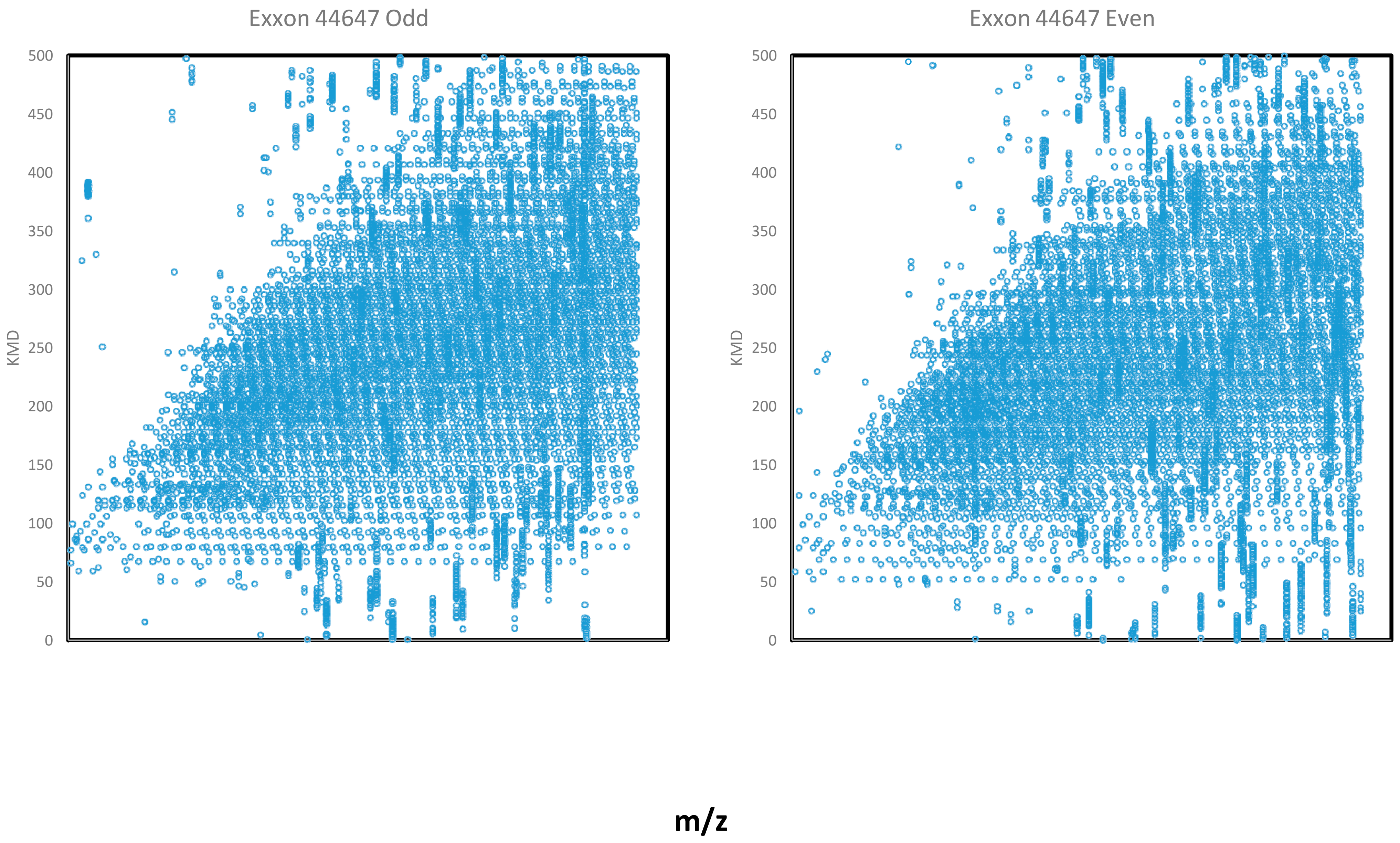

Figure S11A. Negative mode ESI FT-ICR-MS spectrum of organics extracted from the reaction of 7.4 M glucose heated at 85° C for eight days.

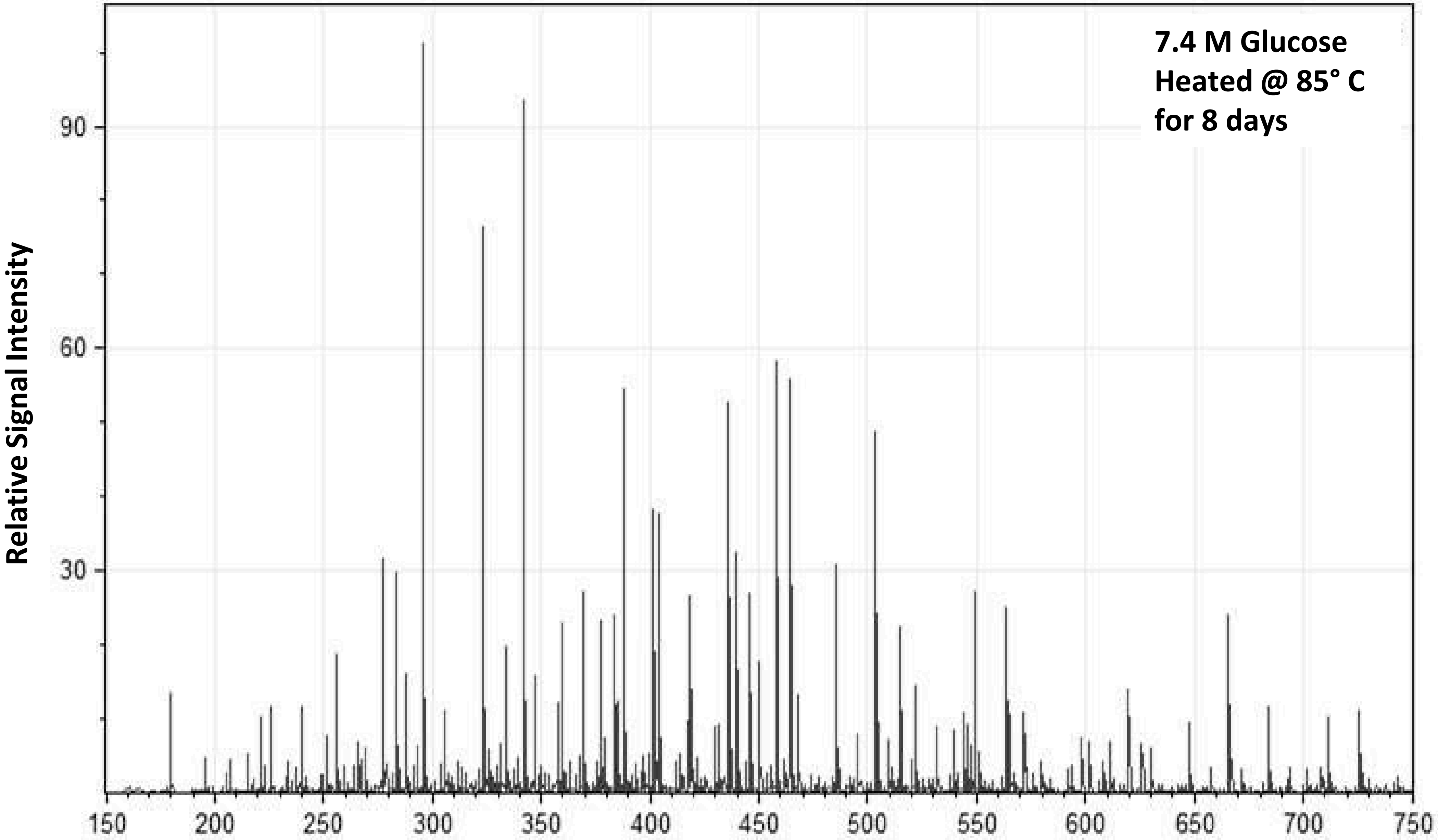

Figure S11B. Odd and even Kendrick Mass Defect plots of the organics extracted from the reaction of 7.4 M glucose heated at 85° C for eight days.

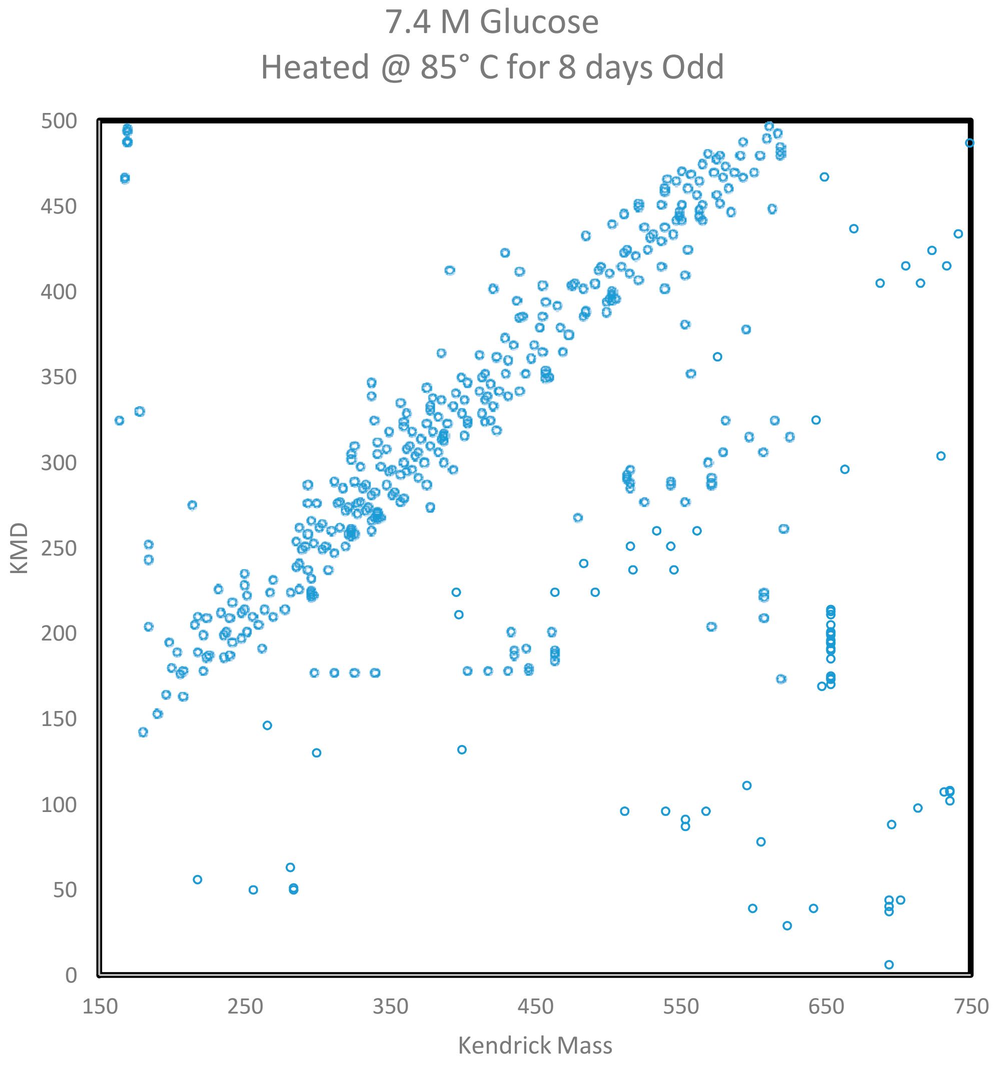

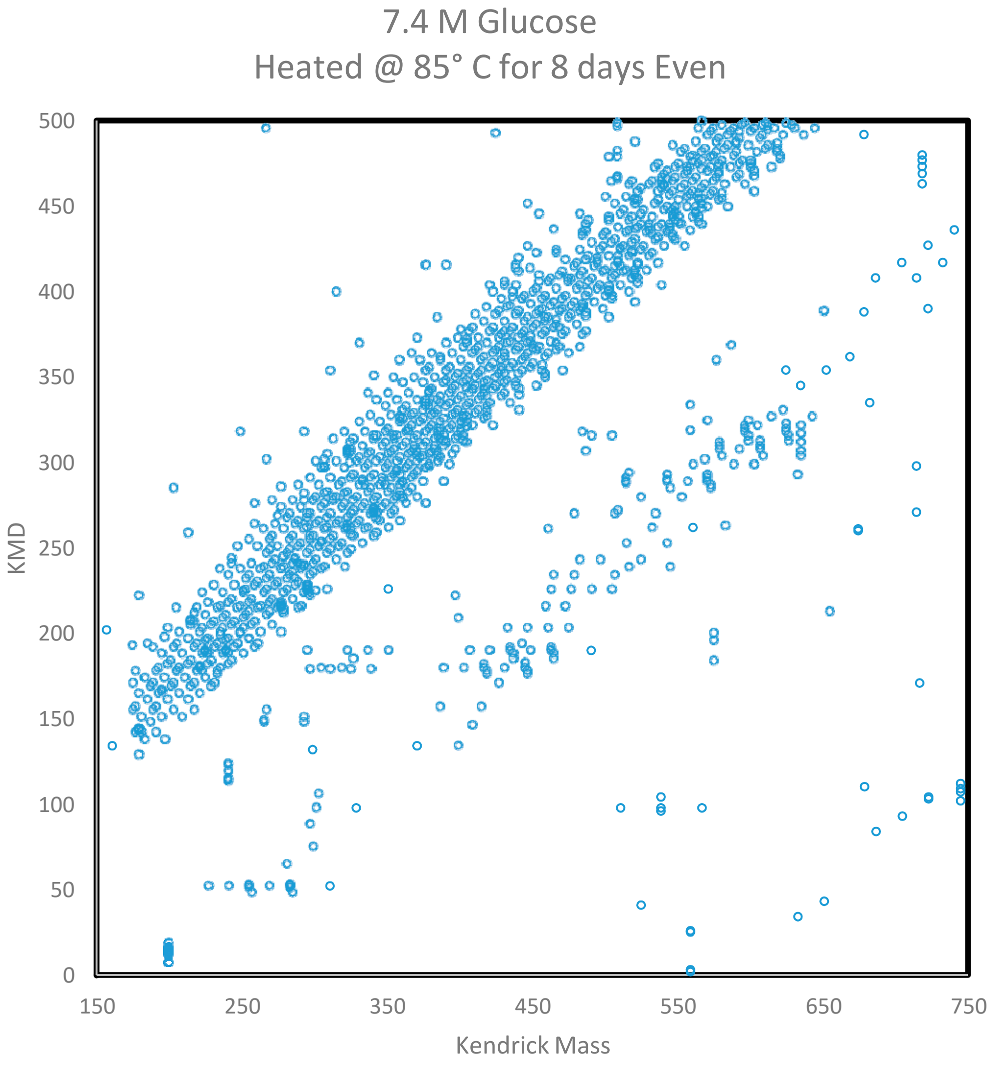

34 Glucose dry heated 150 C 2days

Figure S12A. Negative mode ESI FT-ICR-MS spectrum of organics extracted from the reaction of 3.3 M glucose and 3.3 M Glycine heated at 85° C for eight days.

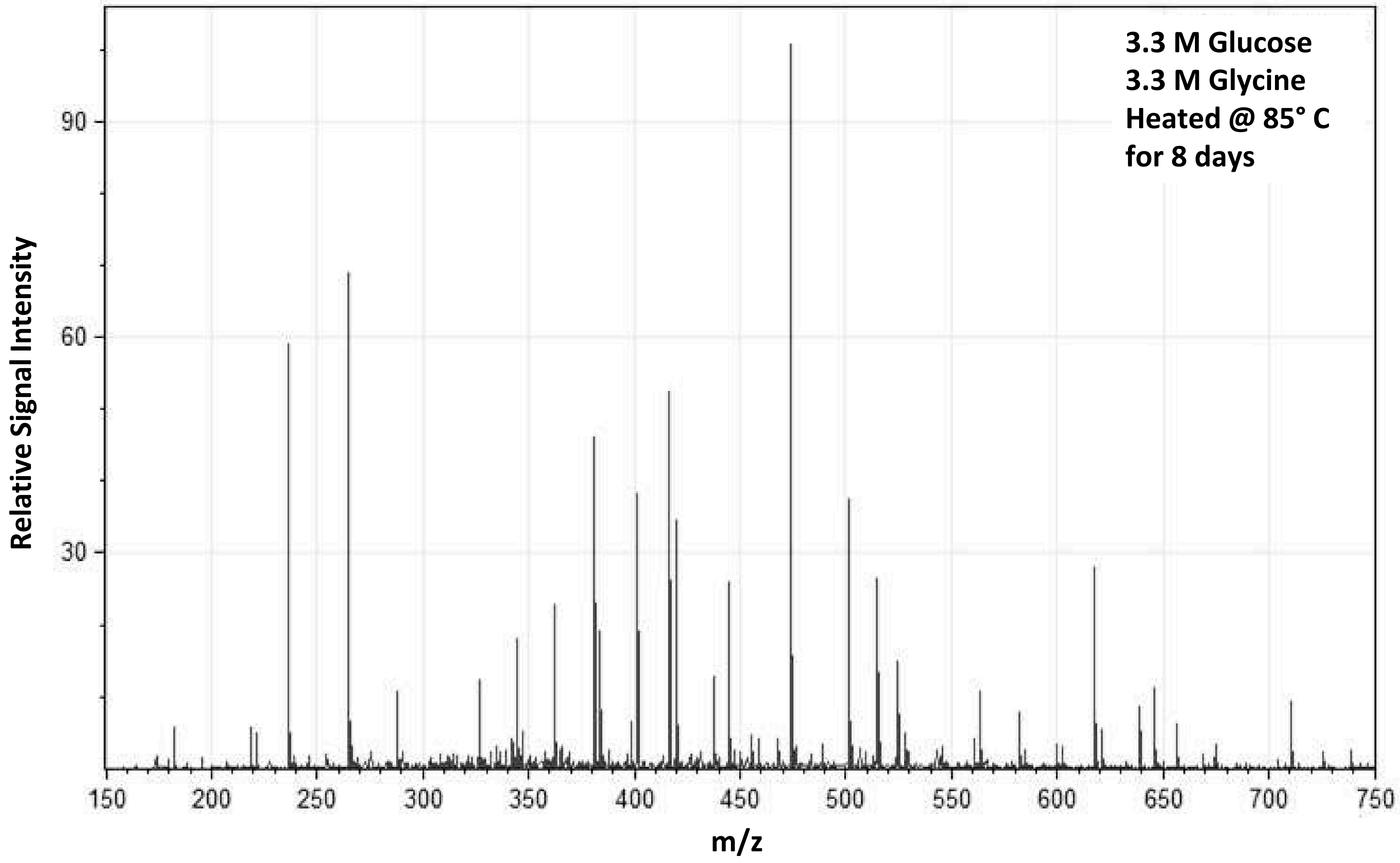

Figure S12B. Odd and even Kendrick Mass Defect plots of the organics extracted from the reaction of 3.3 M glucose and 3.3 M Glycine heated at 85° C for eight days.

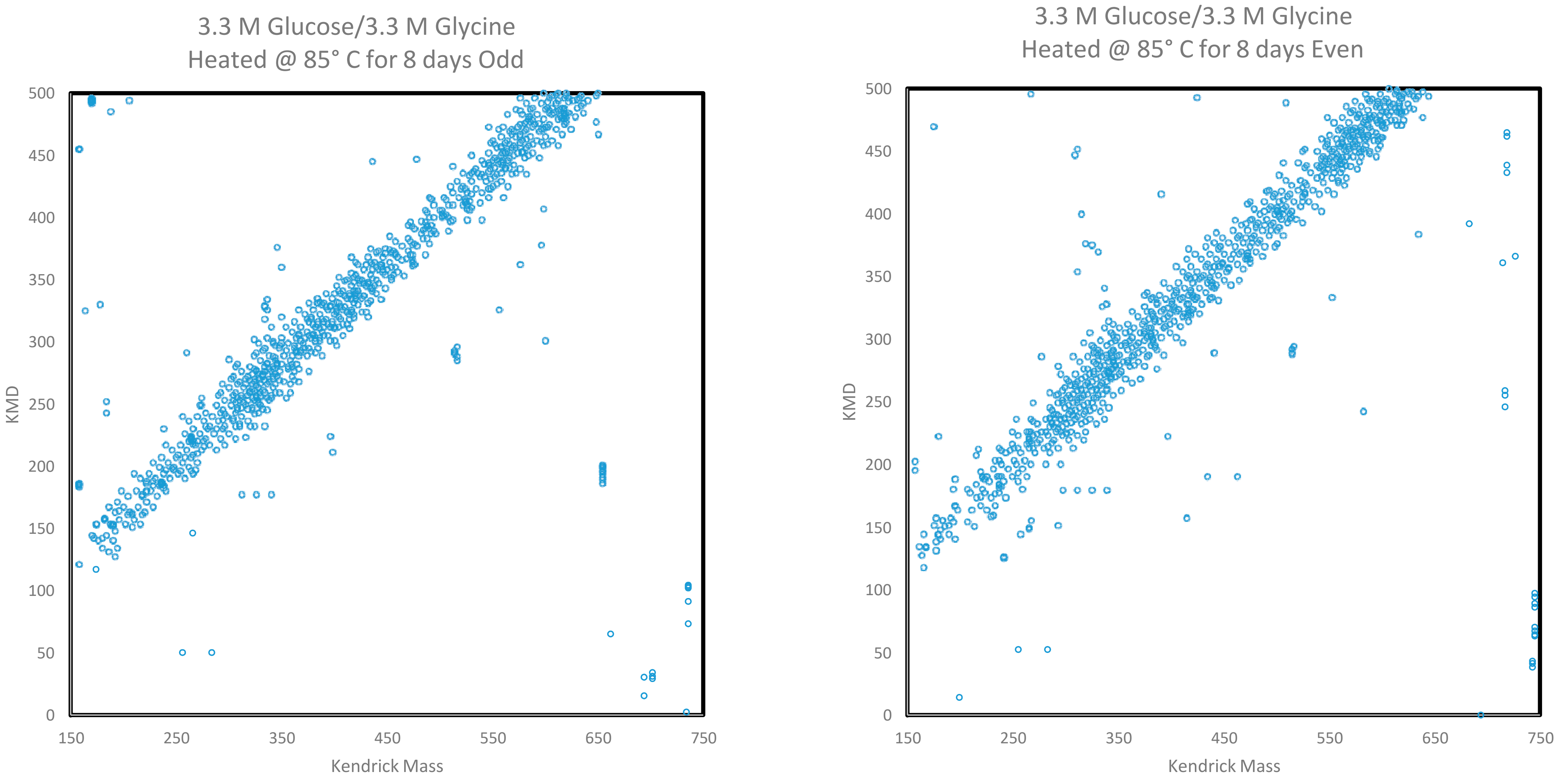

35 Glucose + Glycine wet

Figure S13A. Negative mode ESI FT-ICR-MS spectrum of organics extracted from the reaction of 200 mg glucose and 84 mg glycine dry heated at 150° C for eight days.

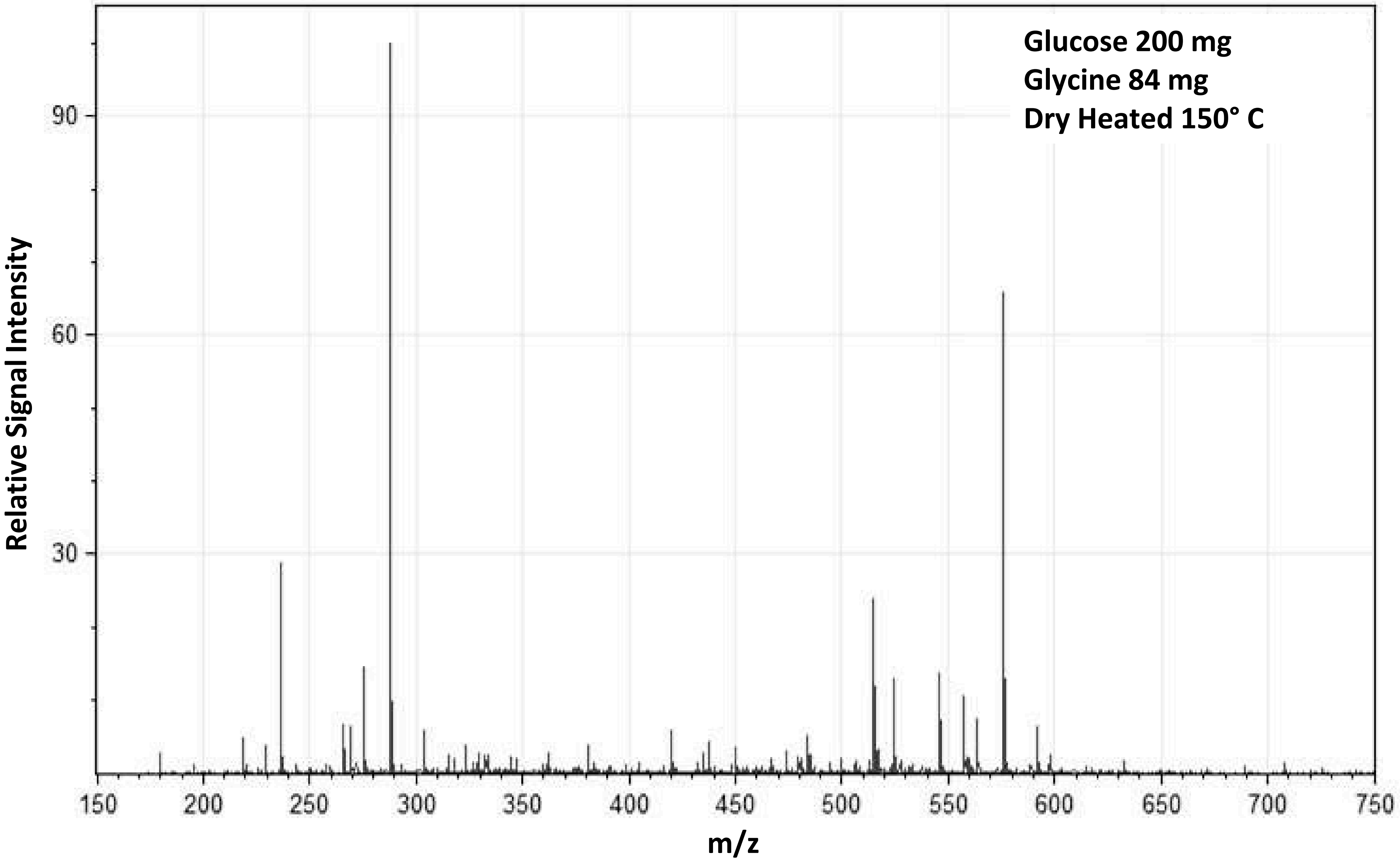

Figure S13B. Odd and even Kendrick Mass Defect plots of the organics extracted from the reaction of 200 mg glucose and 84 mg glycine dry heated at 150° C for eight days.

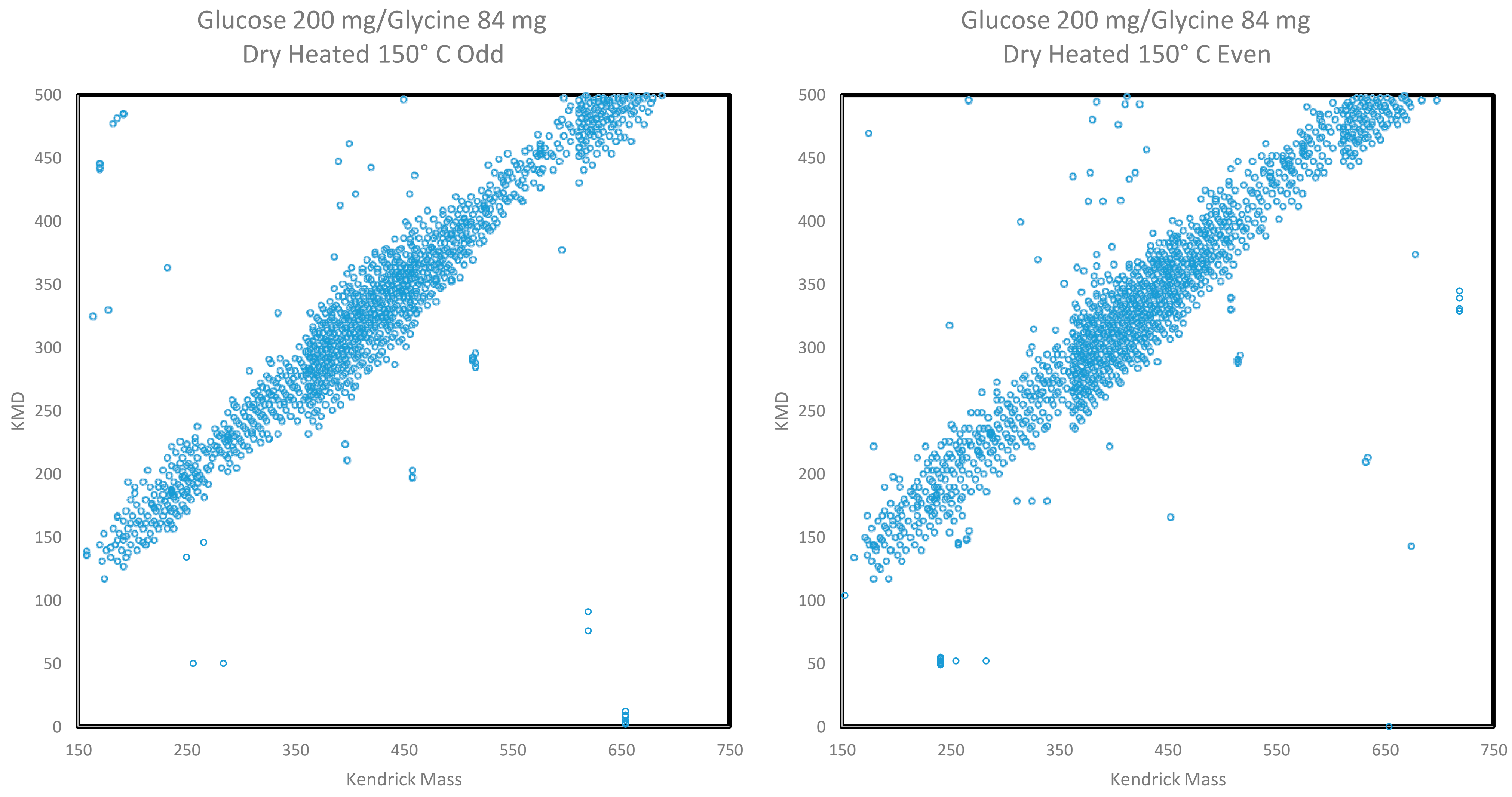

36 Glucose + Glycine Dry

Figure S14A. Negative mode ESI FT-ICR-MS spectrum of organics extracted from the reaction of 3.3 M glucose and 3.3 M NH_4OH heated at 85° C for eight days.

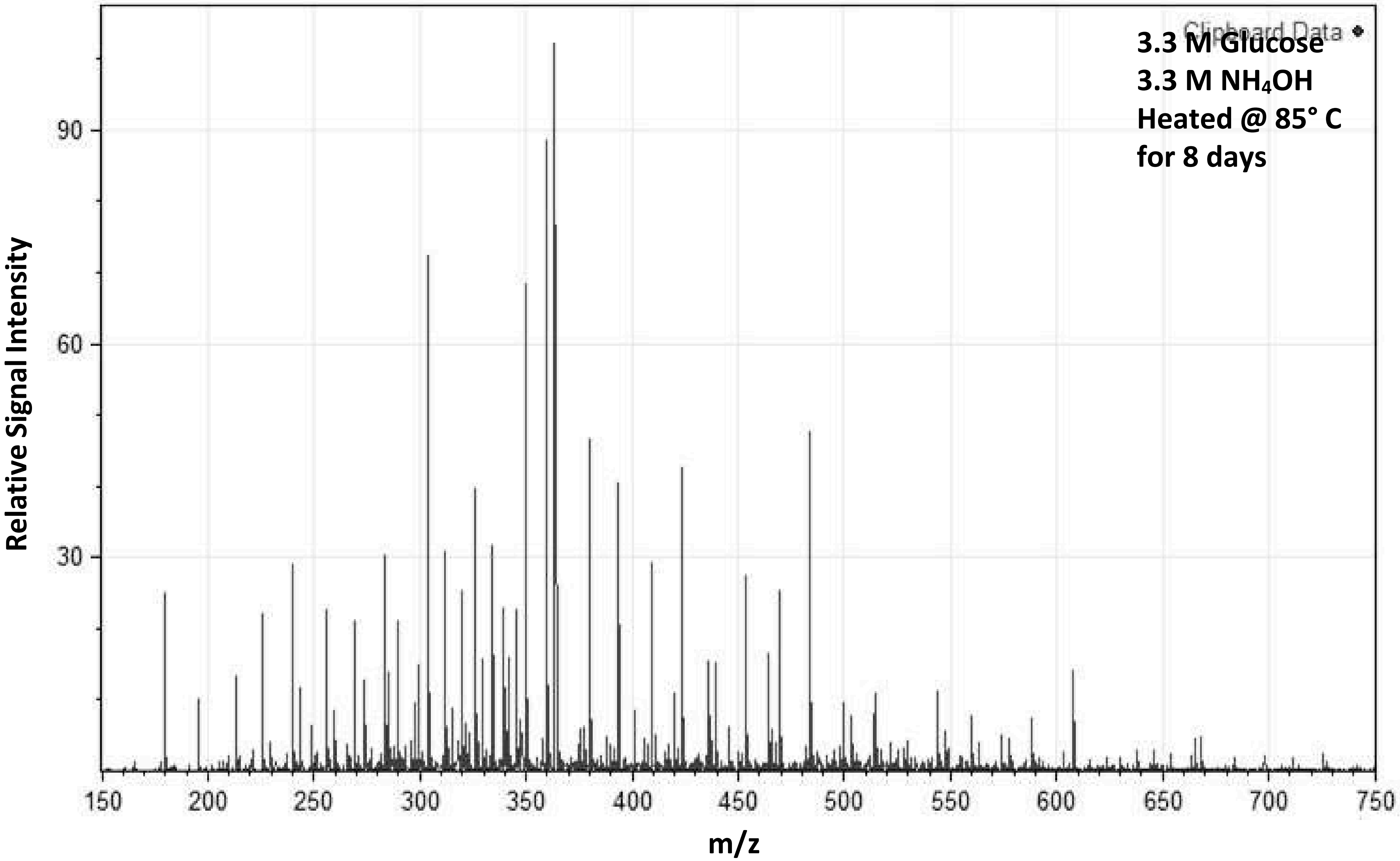

Figure S14B. Odd and even Kendrick Mass Defect plots of the organics extracted from the reaction of 3.3 M glucose and 3.3 M NH_4OH heated at 85° C for eight days.

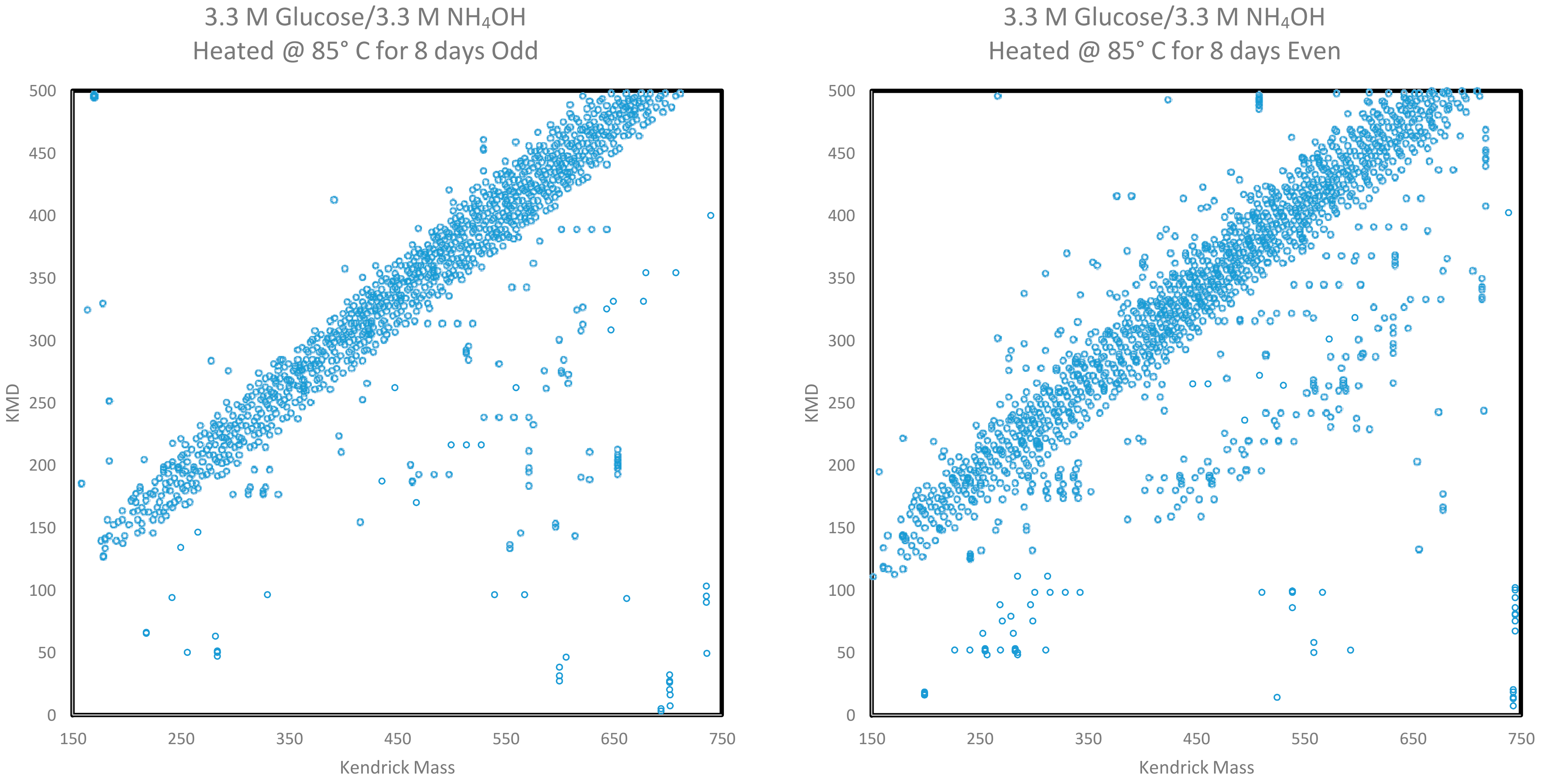

37 Glucose + NH_4OH

Figure S15A. Negative mode ESI FT-ICR-MS spectrum of organics extracted from the reaction of 3.3 M glucose heated at 150° C for eight days.

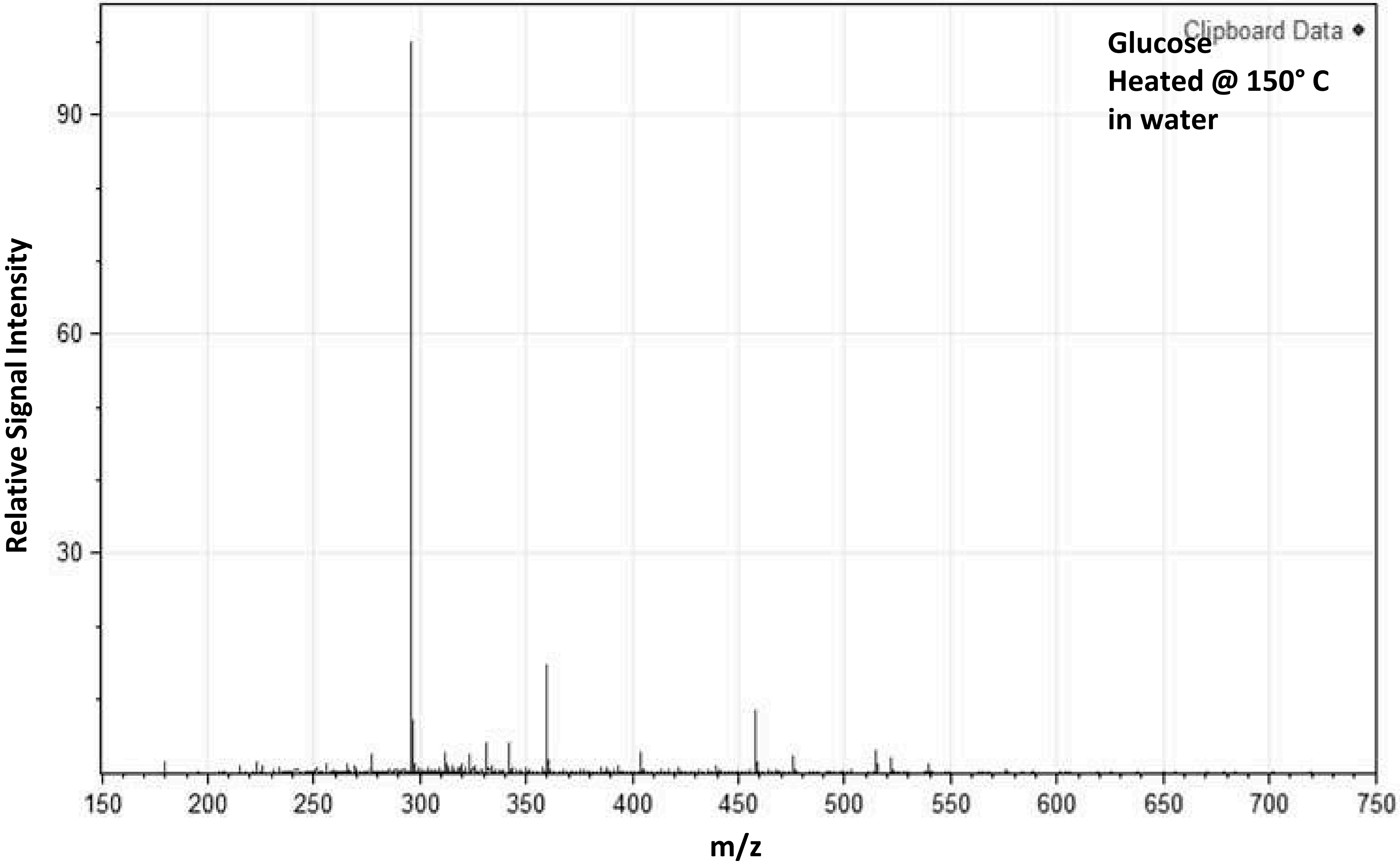

Figure S15B. Odd and even Kendrick Mass Defect plots of the organics extracted from the reaction of 3.3 M glucose heated at 150° C for eight days.

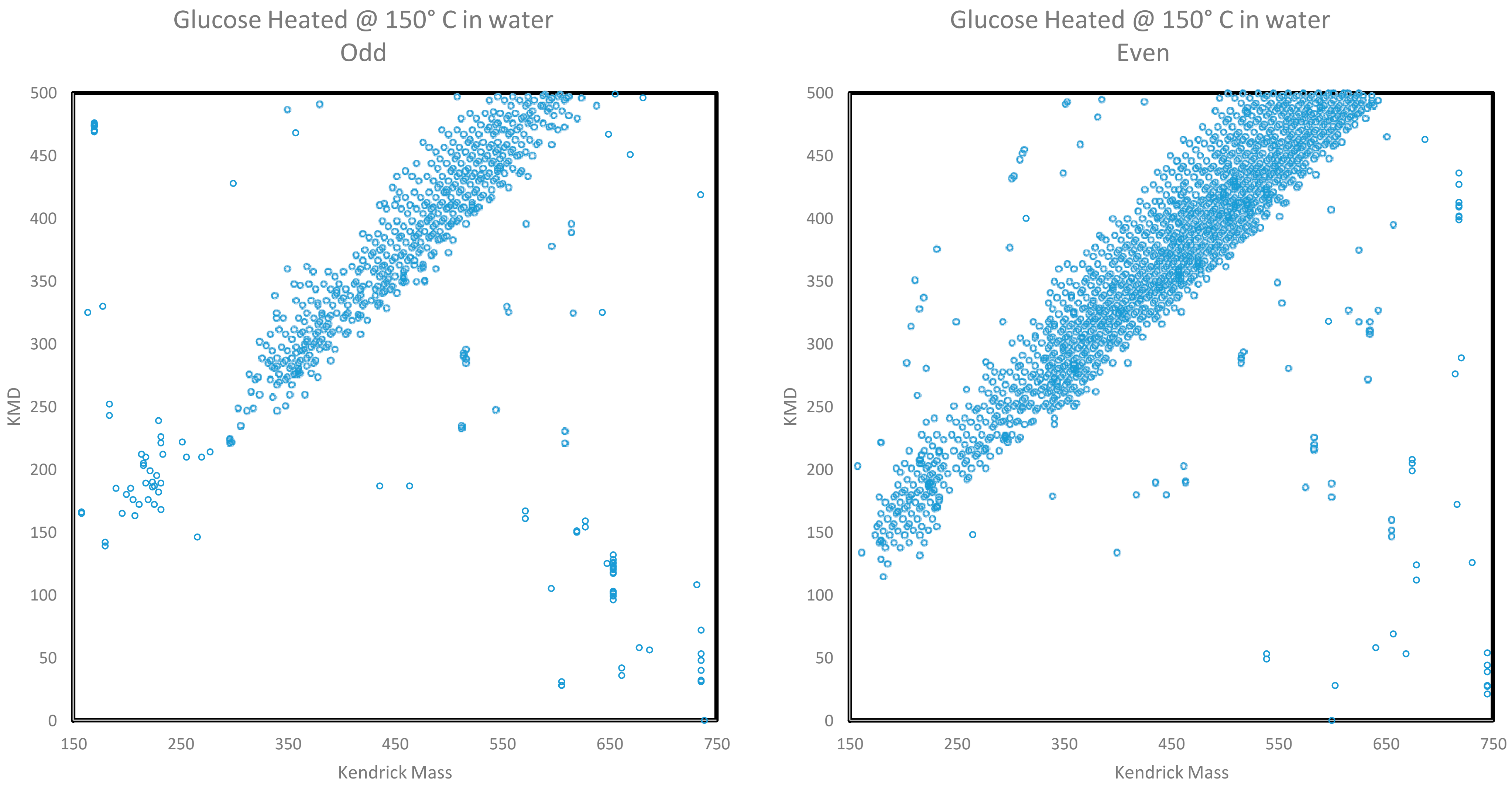

Sample 38 Glucose/Water 150° C

Figure S16A. Negative mode ESI FT-ICR-MS spectrum of organics extracted from the reaction of 1.95 M paraformaldehyde, 0.97 M glycolaldehyde and 0.195 M $Ca(OH)_2$ heated at 150° C for eight days.

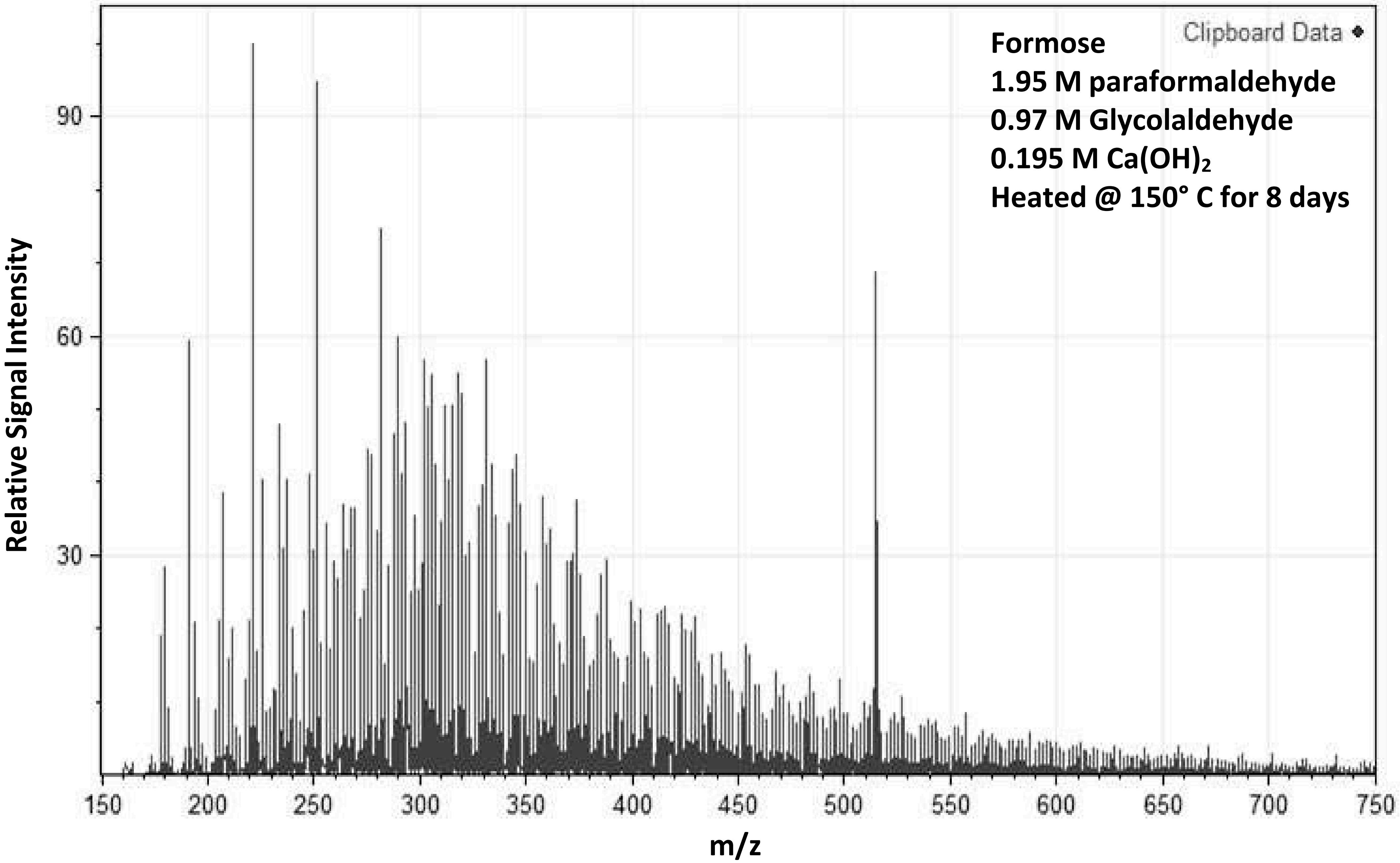

Figure S16B. Odd and even Kendrick Mass Defect plots of the organics extracted from the reaction of 1.95 M paraformaldehyde, 0.97 M glycolaldehyde and 0.195 M $Ca(OH)_2$ heated at 150° C for eight days.

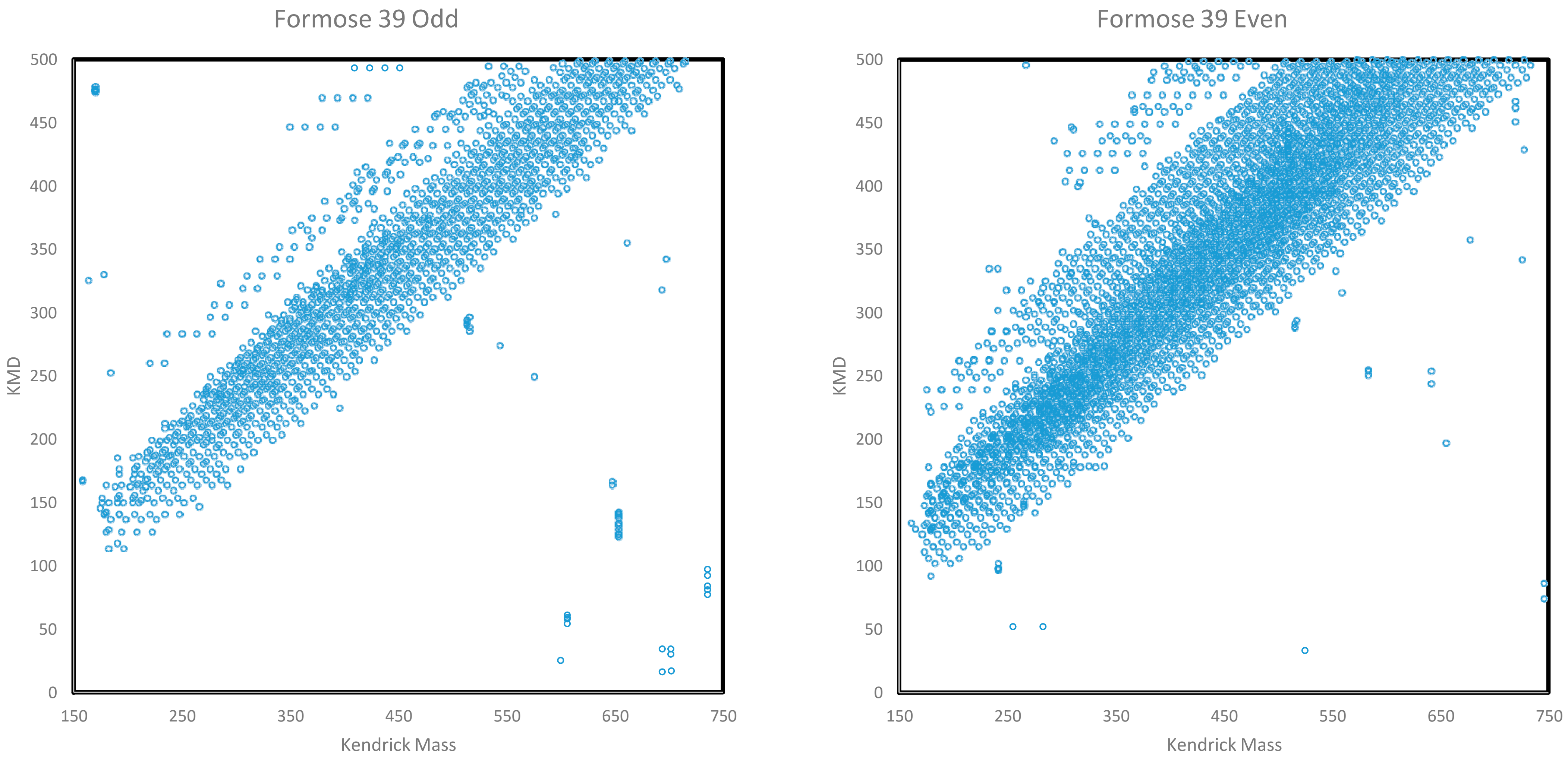

39 Formose 150 C

Figure S17A. Negative mode ESI FT-ICR-MS spectrum of organics extracted from the reaction of 1.95 M paraformaldehyde, 0.97 M glycolaldehyde, 0.195 M $Ca(OH)_2$ and 0.39 M NH_4OHheated at 150° C for eight days.

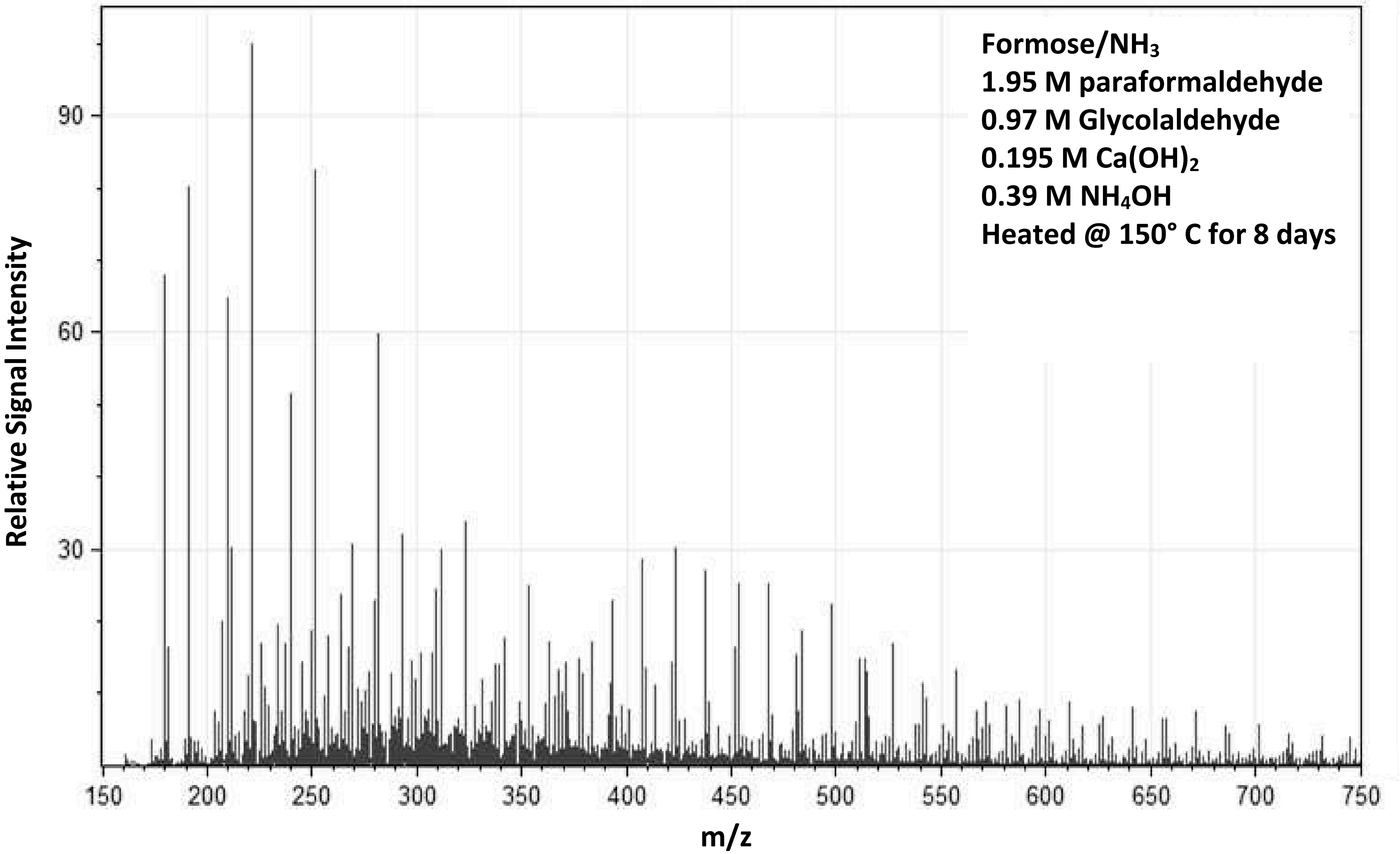

Figure S17B. Odd and even Kendrick Mass Defect plots of the organics extracted from the reaction of 1.95 M paraformaldehyde, 0.97 M glycolaldehyde, 0.195 M $Ca(OH)_2$ and 0.39 M NH_4OHheated at 150° C for eight days.

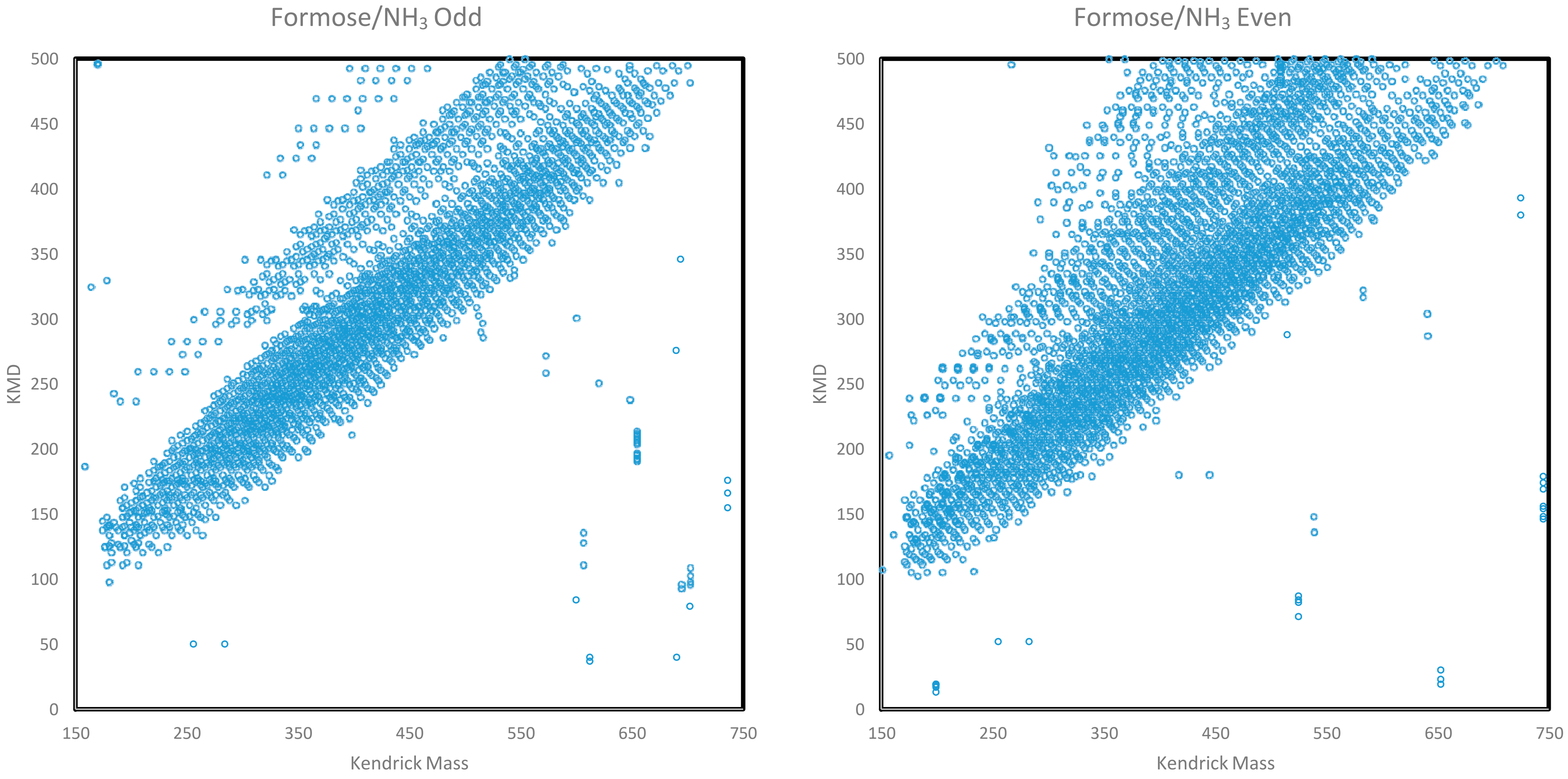

40 Formose/NH_3 150° C

Figure S18A. Negative mode ESI FT-ICR-MS spectrum of organics extracted from the reaction of 5.55 M NH_4OH, 0.55 M HCHO and 0.05 M CH_3CHO heated at 150° C for 47 hours.

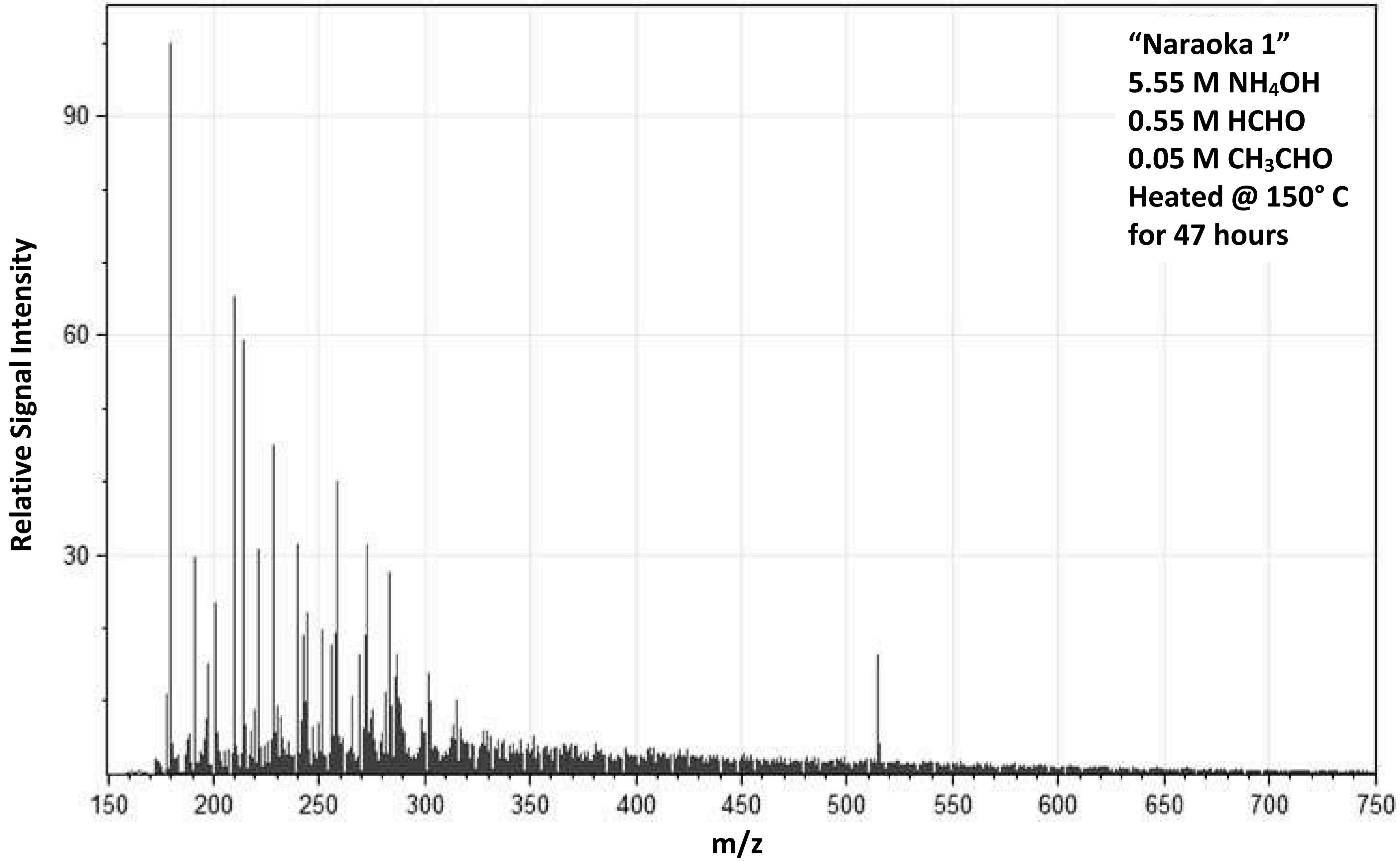

Figure S18B. Odd and even Kendrick Mass Defect plots of the organics extracted from the reaction of 5.55 M NH_4OH, 0.55 M HCHO and 0.05 M CH_3CHO heated at 150° C for 47 hours.

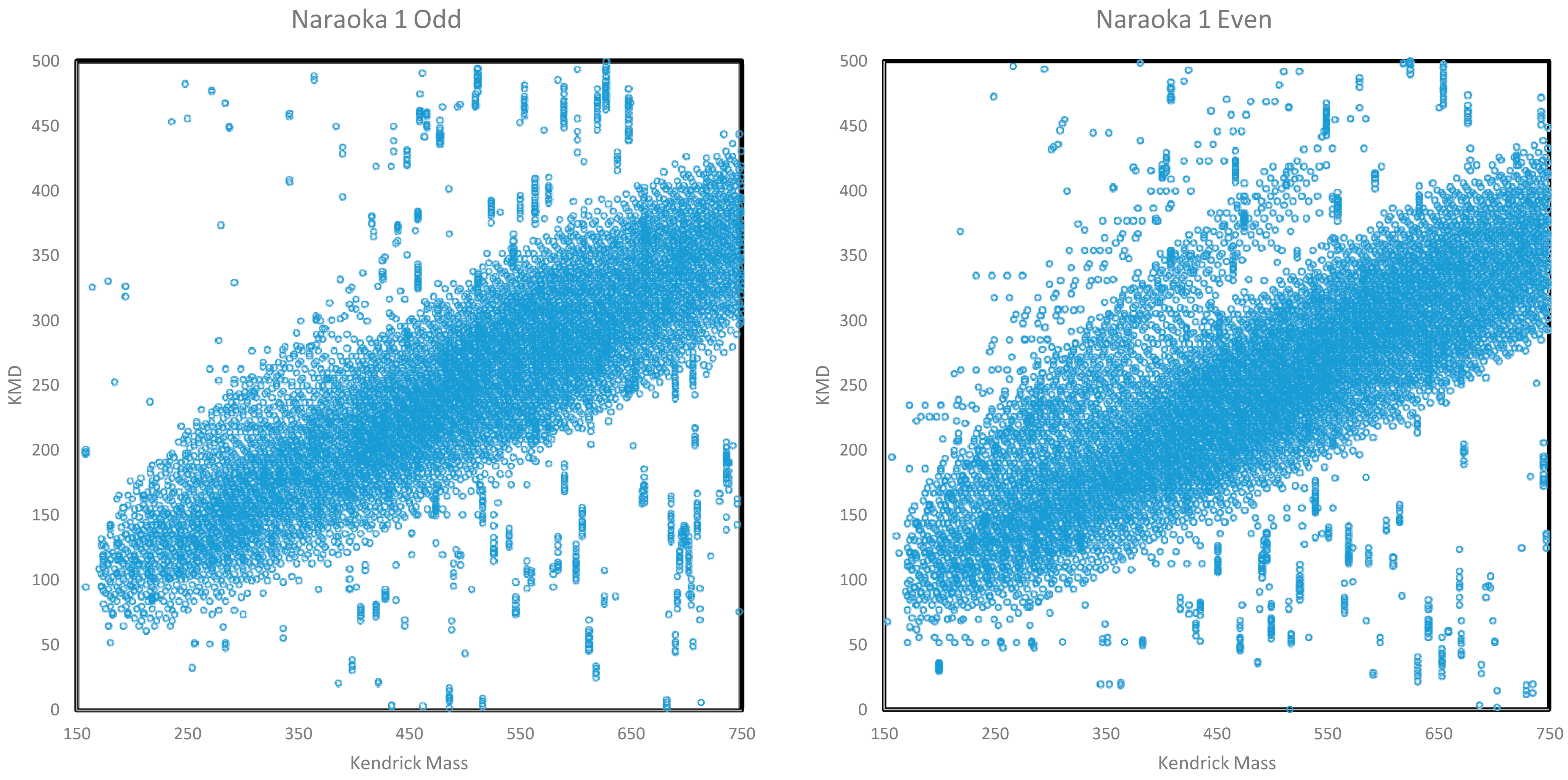

Sample 41 Naraoka 1 (NH_3+HCHO+CH_3CHO)

Figure S19A. Negative mode ESI FT-ICR-MS spectrum of organics extracted from the reaction of 5.55 M NH_4OH, 0.55 M HCHO, 0.05 M CH_3CHO and 0.05 M glycolaldehyde heated at 150° C for 47 hours.

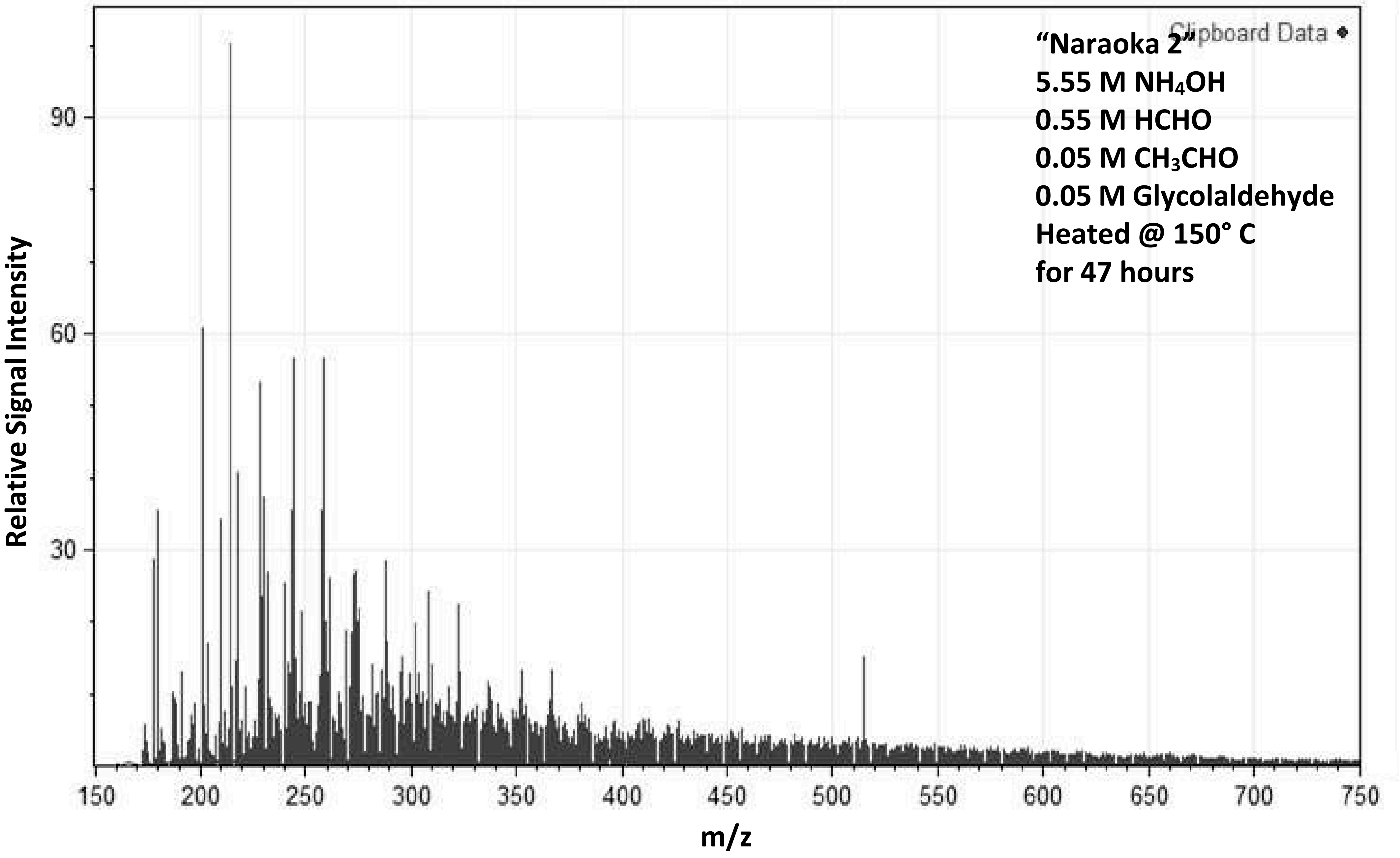

Figure S19B. Odd and even Kendrick Mass Defect plots of the organics extracted from the reaction of 5.55 M NH_4OH, 0.55 M HCHO, 0.05 M CH_3CHO and 0.05 M glycolaldehyde heated at 150° C for 47 hours.

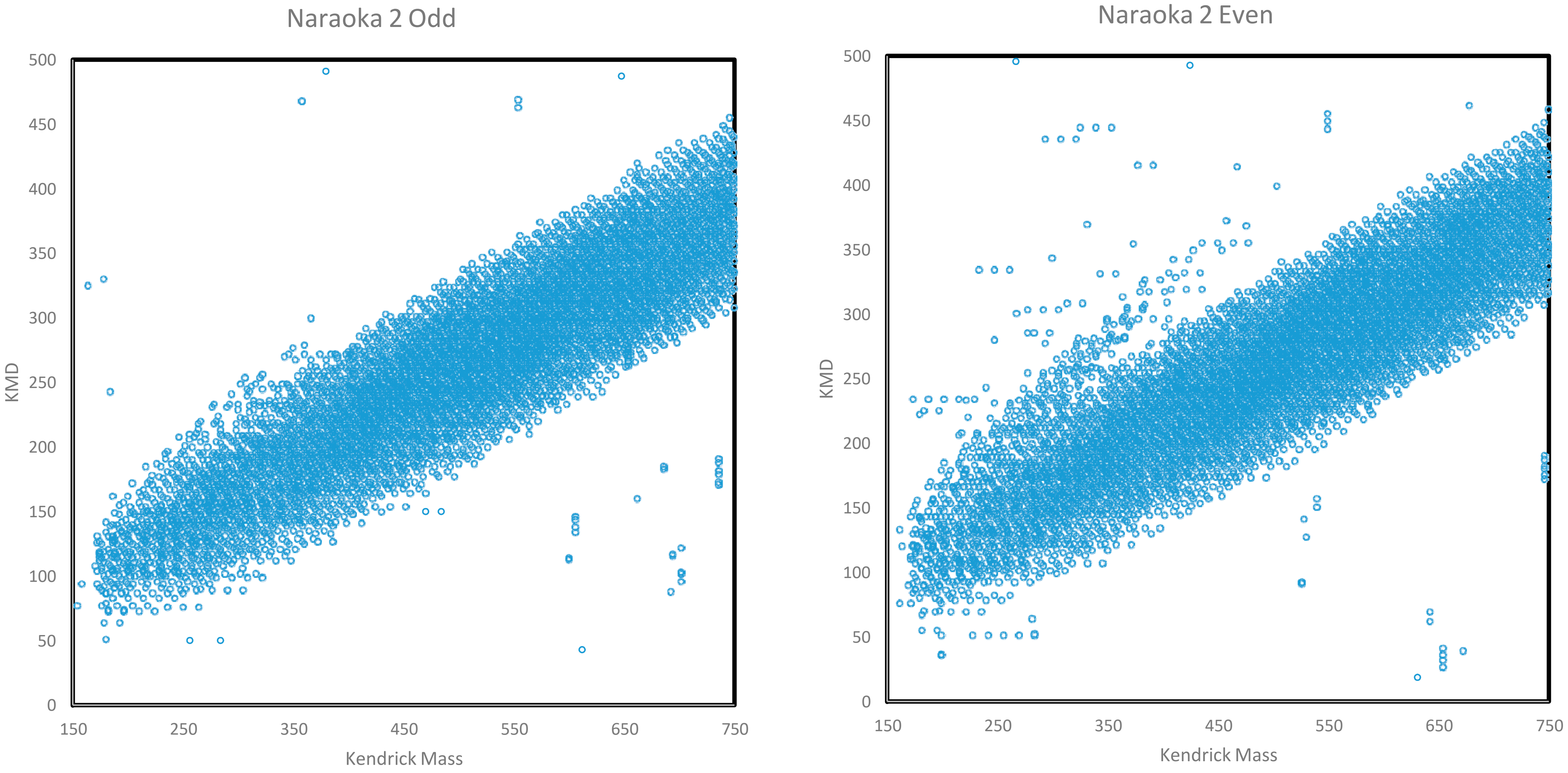

Sample 42 Naraoka 2 (NH_3+HCHO+CH_3CHO + Glycolaldehyde)

Figure S20A. Negative mode ESI FT-ICR-MS spectrum of organics extracted from commercial 70% (~13.2 M) aqueous glycolonitrile solution which had been left sealed from the manufacturer after reaction at ~25° C for ~ 20 years.

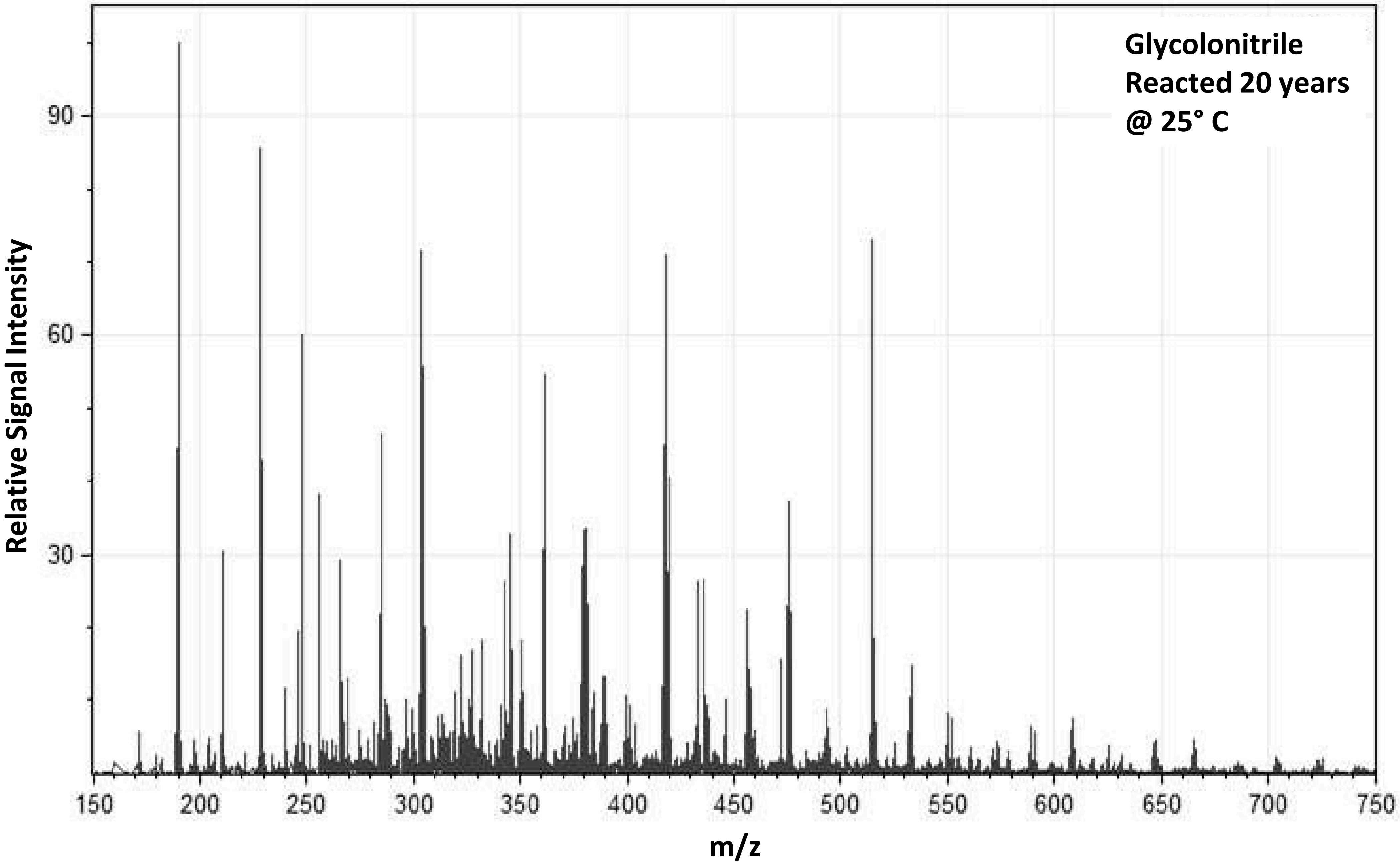

Figure S20B. Odd and even Kendrick Mass Defect plots of the organics extracted from commercial 70% (~13.2 M) aqueous glycolonitrile solution which had been left sealed from the manufacturer after reaction at ~25° C for ~ 20 years.

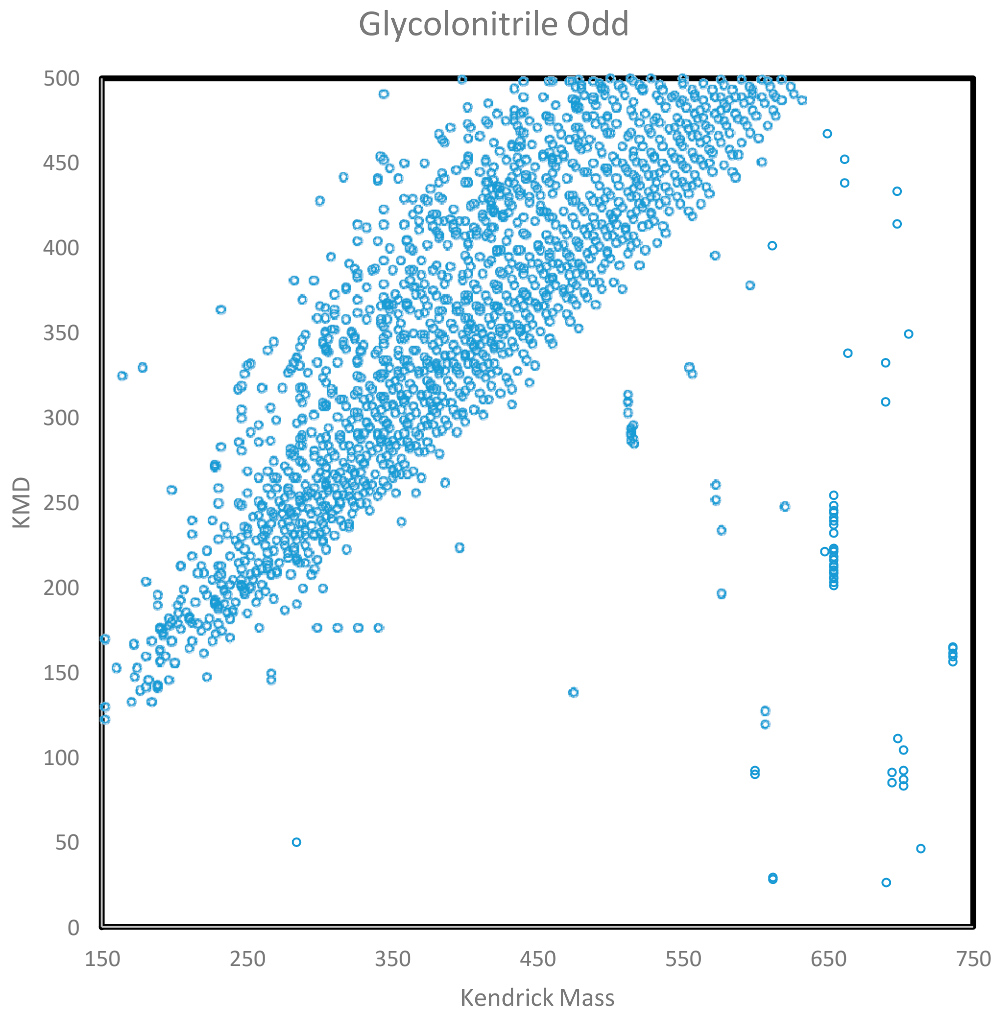

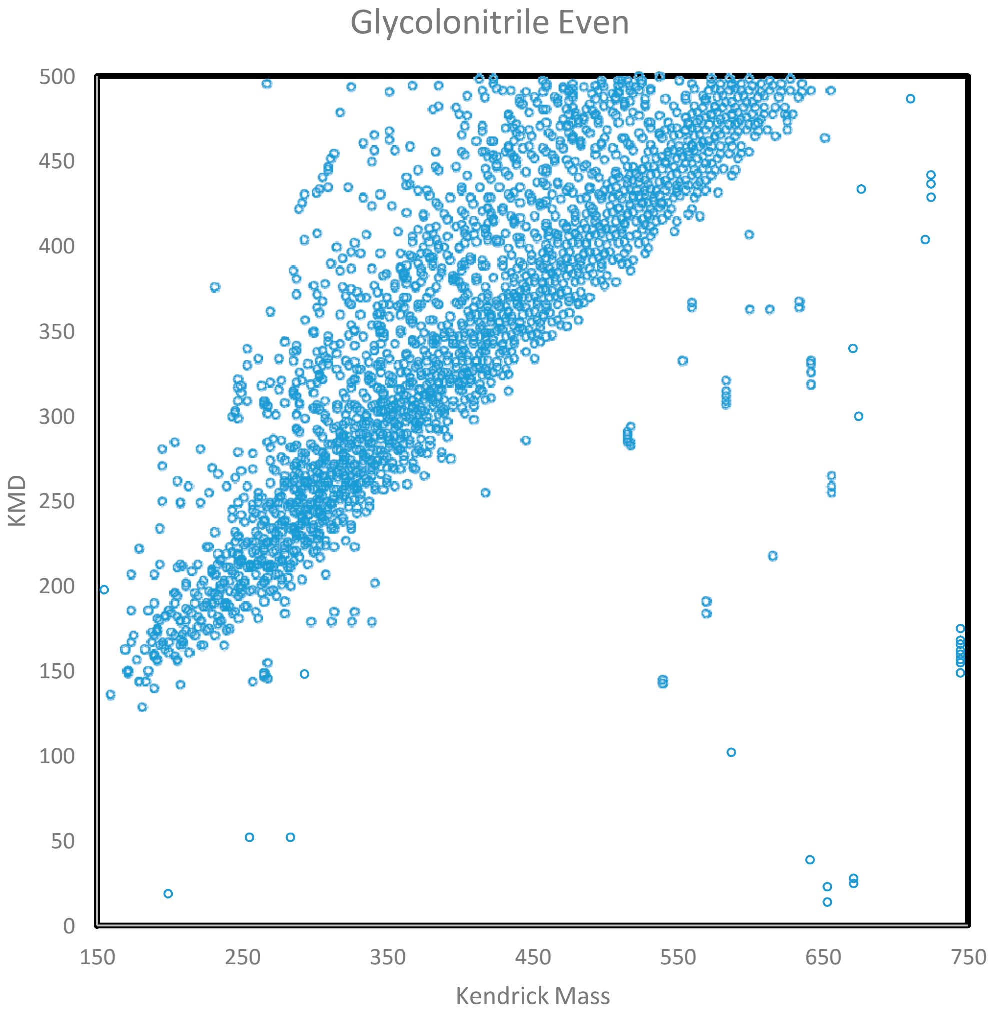

43 Glycolonitrile (20 year reaction in water at 25° C)

Figure S21A. Negative mode ESI FT-ICR-MS spectrum of organics extracted from a Miller Urey electric discharge reaction prepared from CH_4, NH_3 and H_2O (see reference 49 in the main text) and frozen at -20° C for ~25 years.

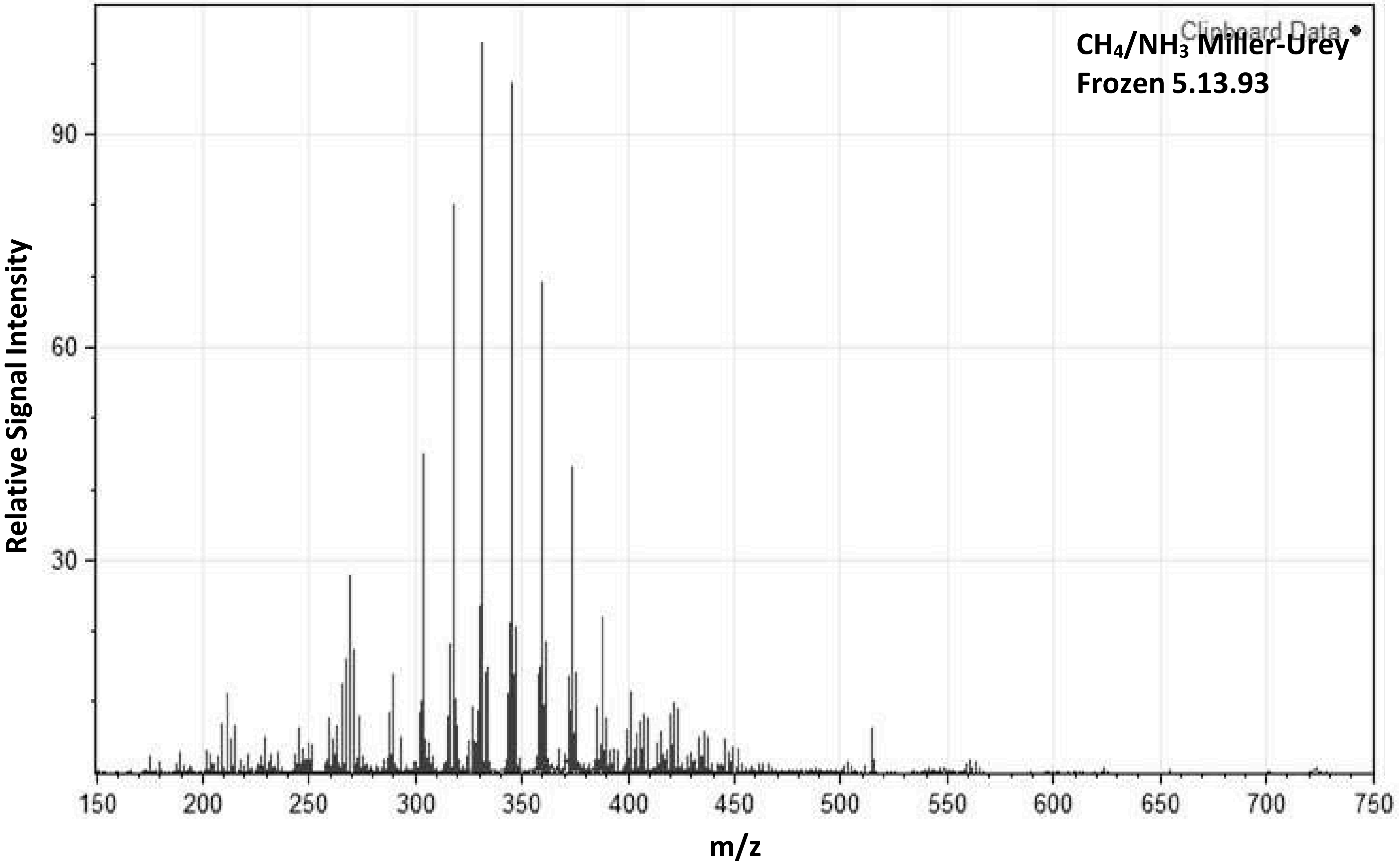

Figure S21B. Odd and even Kendrick Mass Defect plots of the organics extracted from a Miller Urey electric discharge reaction prepared from CH_4, NH_3 and H_2O (see reference 49 in the main text) and frozen at -20° C for ~25 years.

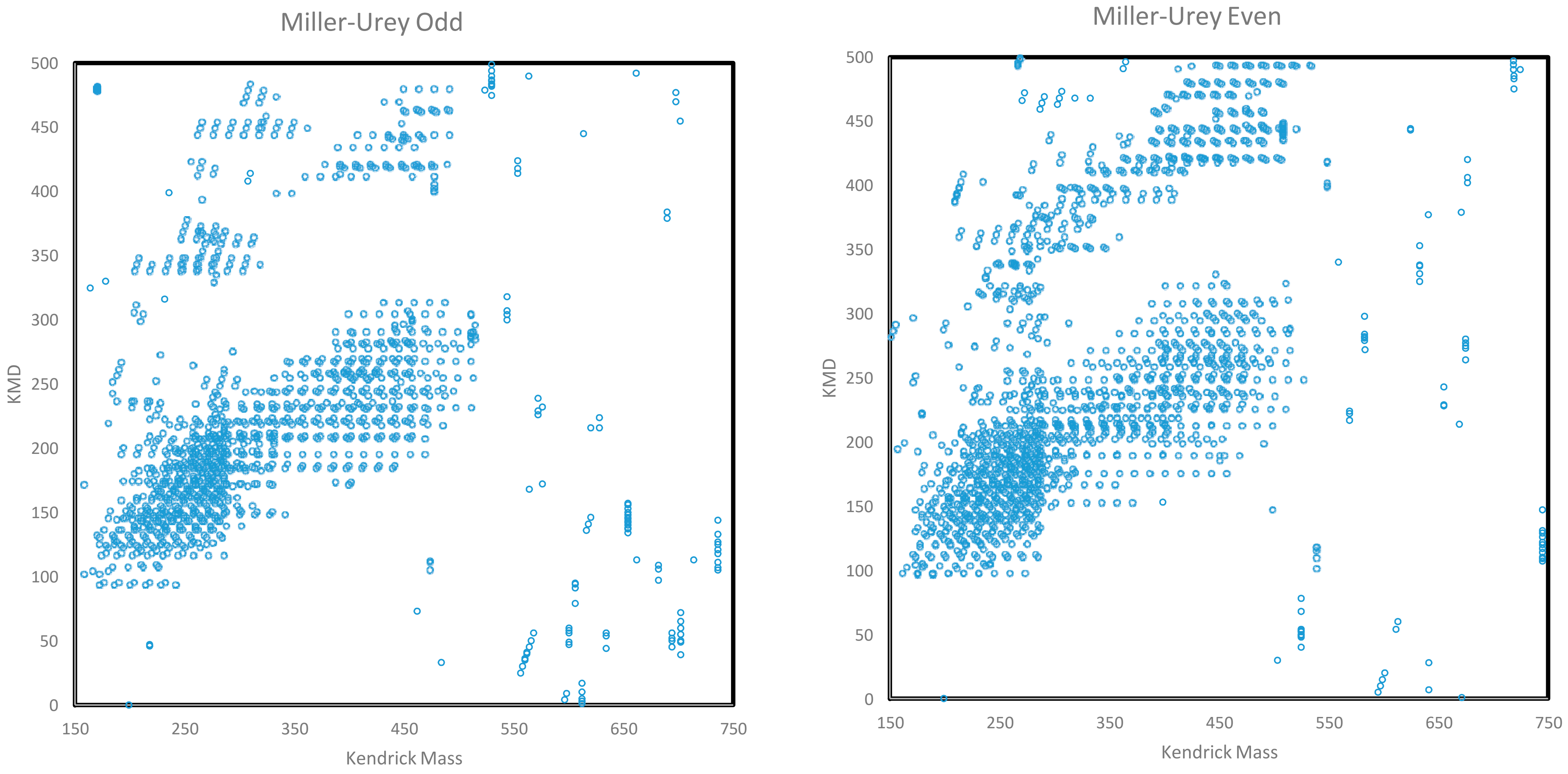

Sample 44 CH_4/NH_3 Miller-Urey, frozen 5.13.93

Figure S22A. Negative mode ESI FT-ICR-MS spectrum of organics extracted from an NH_4CN polymerization prepared from 0.15 M NH_3 and 0.1 M HCN and frozen at -20 C for ~45 years (see reference [49] in the main text).

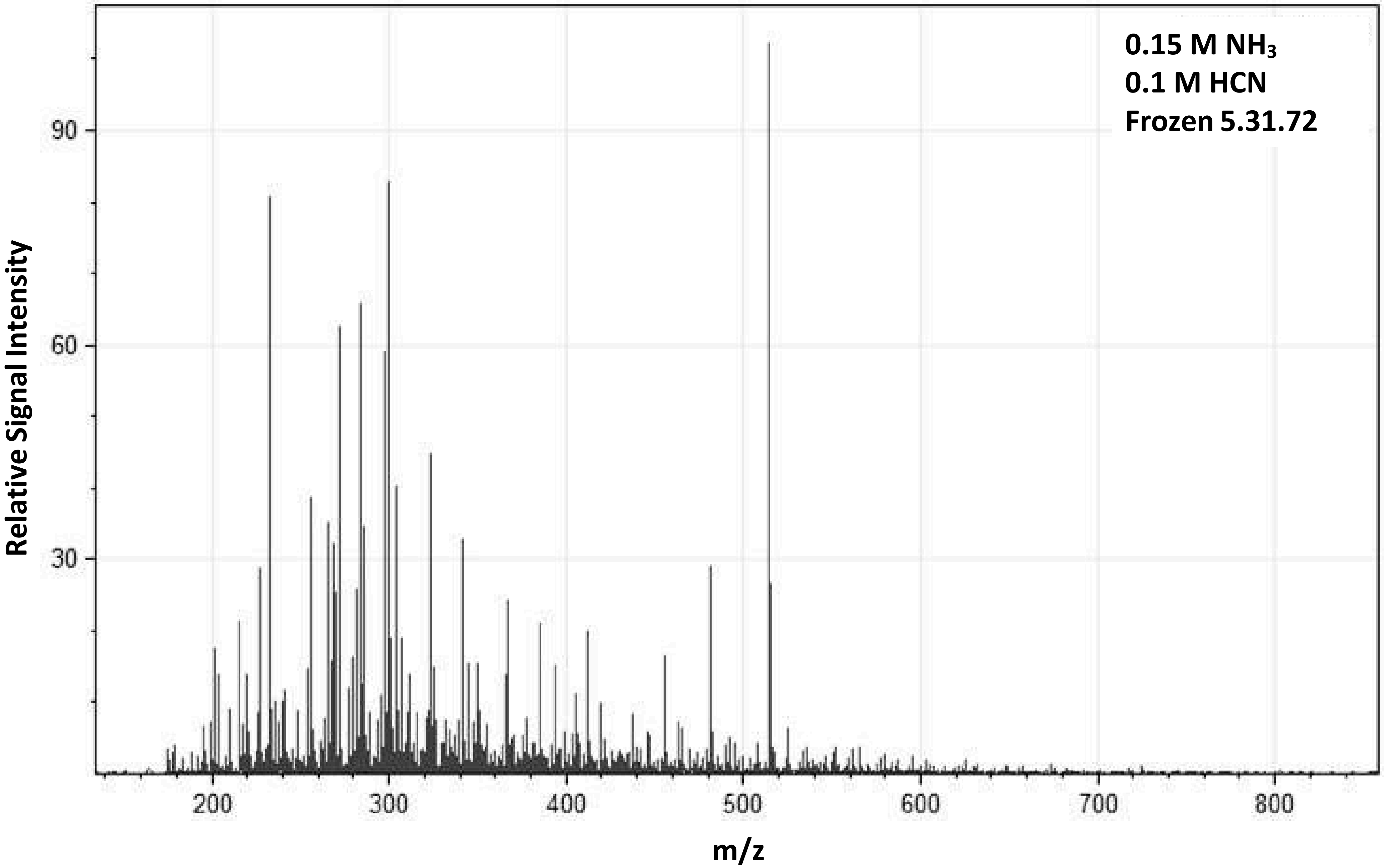

Figure S22B. Odd and even Kendrick Mass Defect plots of the organics extracted from an NH_4CN polymerization prepared from 0.15 M NH_3 and 0.1 M HCN and frozen at -20 C for ~45 years (see reference [49] in the main text).

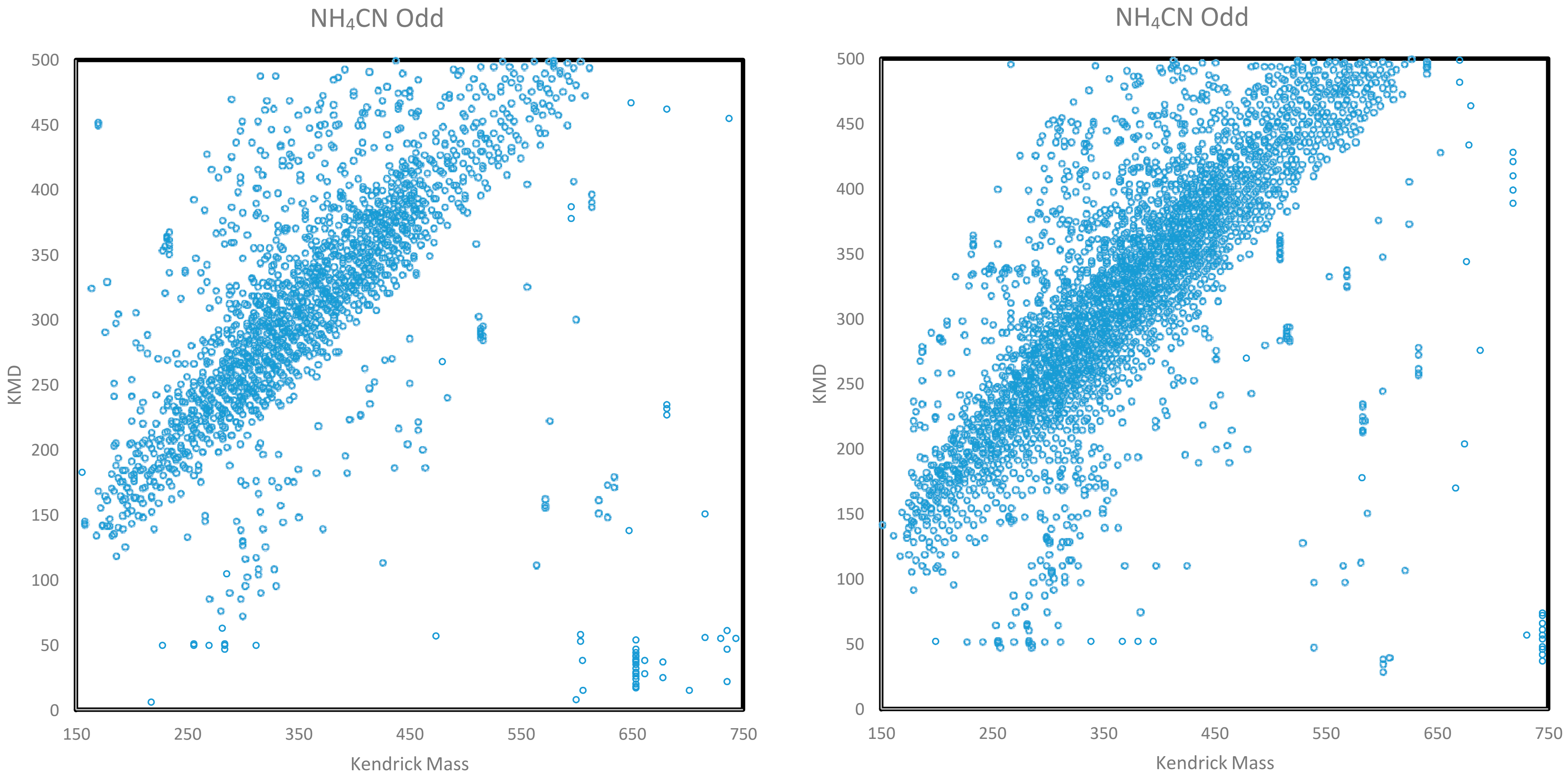

Sample 45 NH4CN (frozen 5.31.72)

Figure S23A. Negative mode ESI FT-ICR-MS spectrum of organics extracted from neat pyruvic acid heated at 150° C for 47 hours.

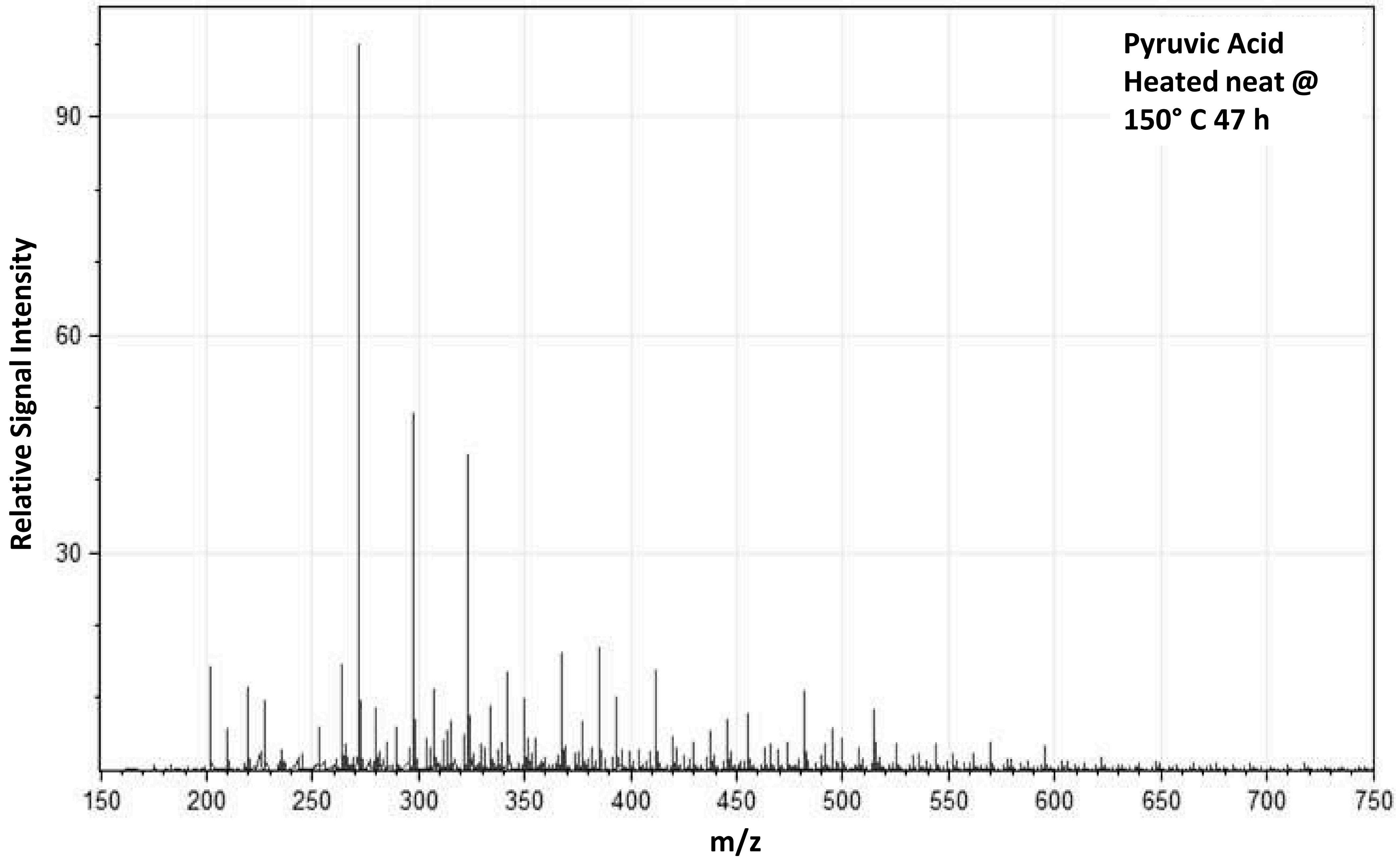

Figure S23B. Odd and even Kendrick Mass Defect plots of the organics extracted from neat pyruvic acid heated at 150° C for 47 hours.

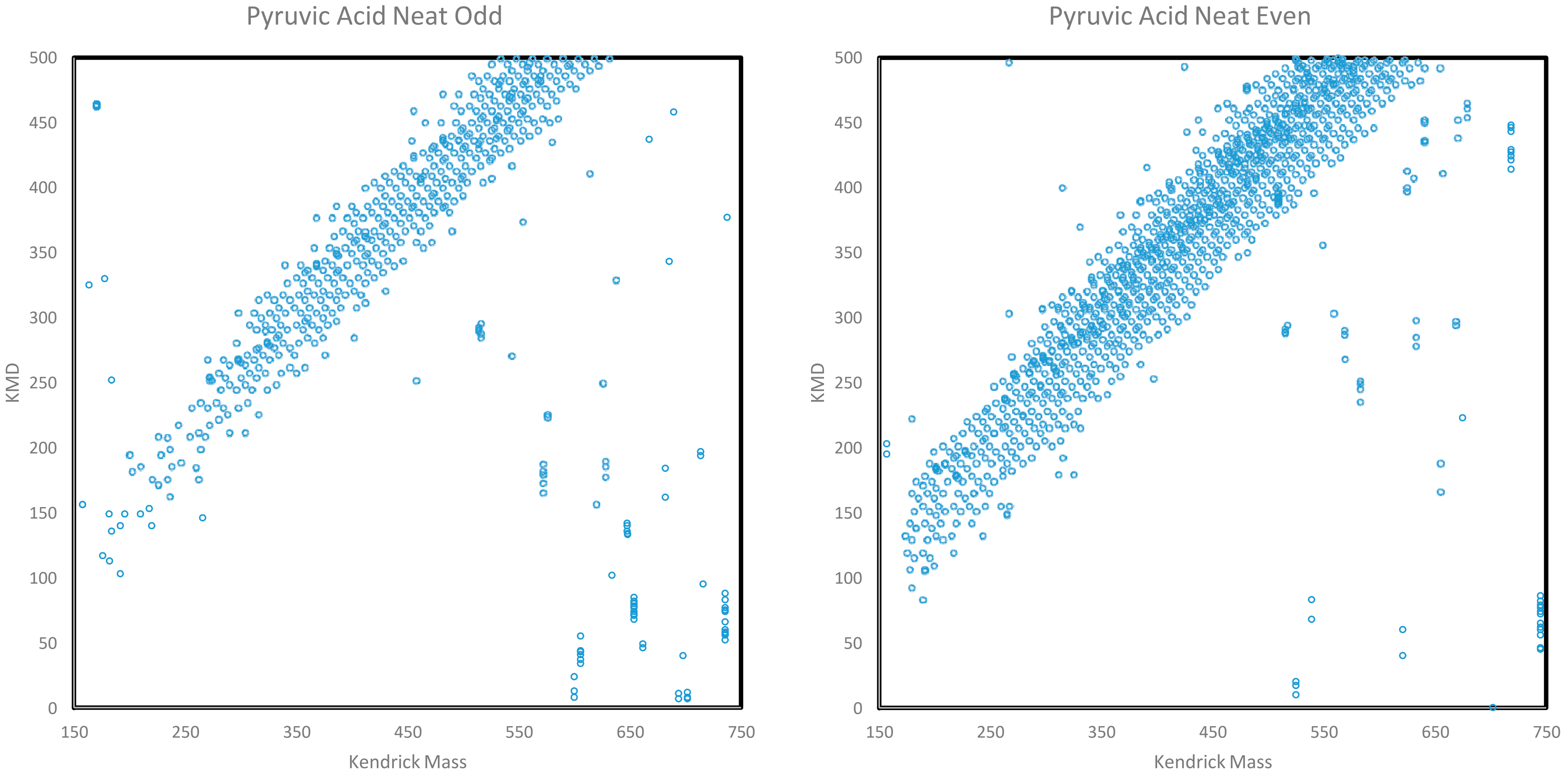

Sample 46 Neat Pyruvic Acid 150°

Figure S24A. Negative mode ESI FT-ICR-MS spectrum of organics extracted from 5.7 M aqueous pyruvic acid heated at 150° C for 47 hours.

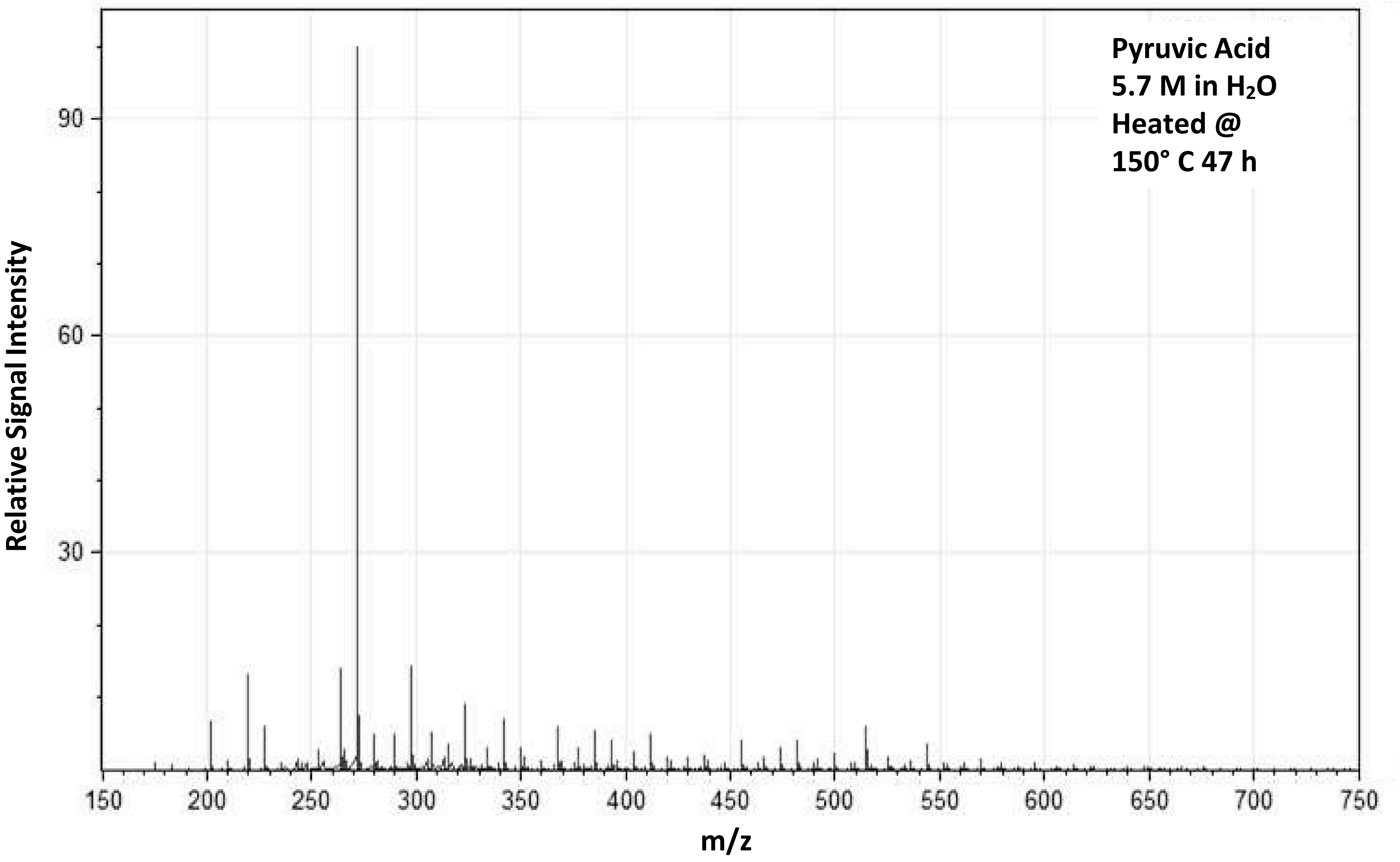

Figure S24B. Odd and even Kendrick Mass Defect plots of the organics extracted from 5.7 M aqueous pyruvic acid heated at 150° C for 47 hours.

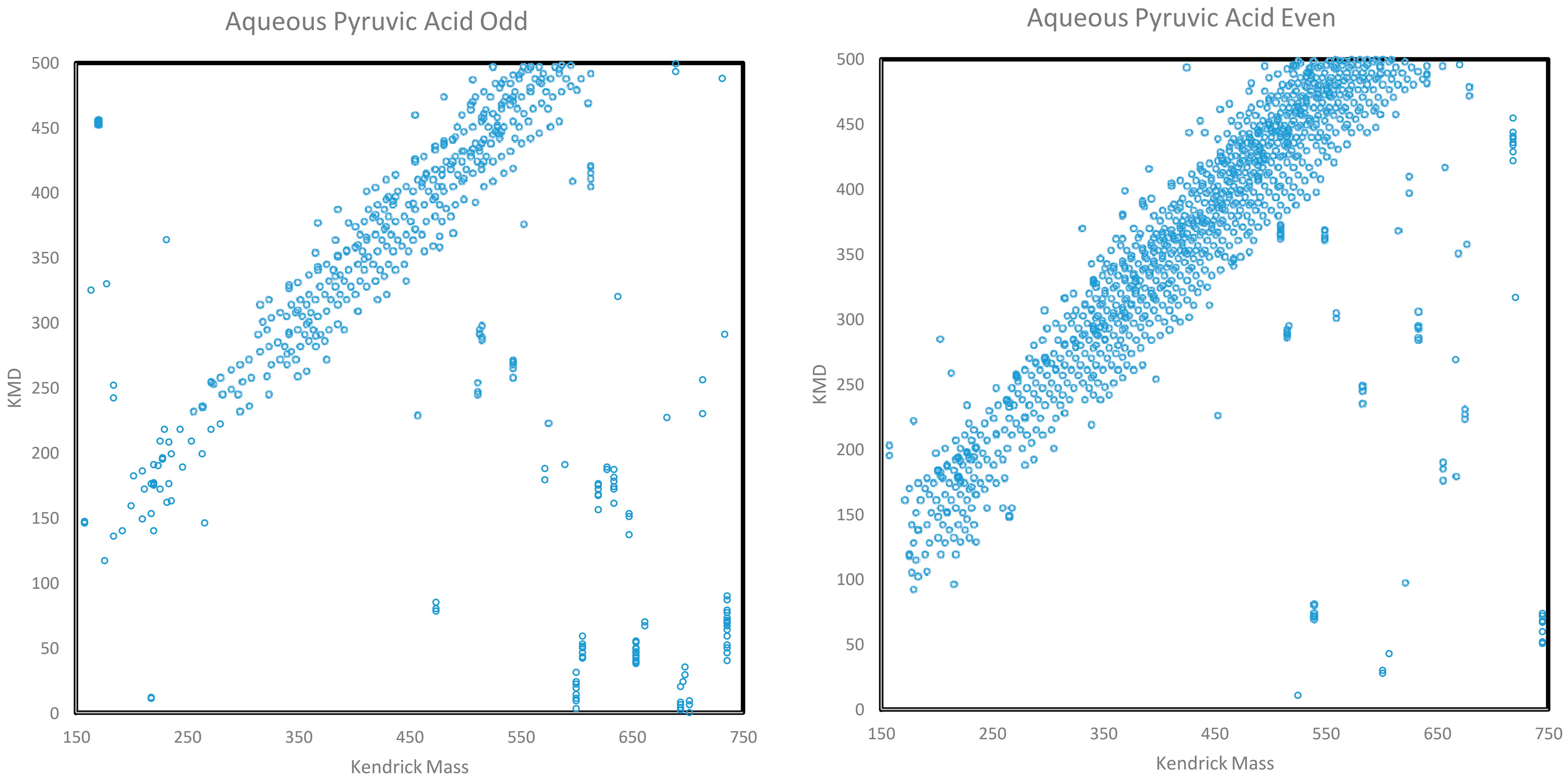

Sample 47 Aqueous pyruvic acid 150° C

Figure S25A. Negative mode ESI FT-ICR-MS spectrum of organics extracted using methanol/water of a sample of the Murchison meteorite.

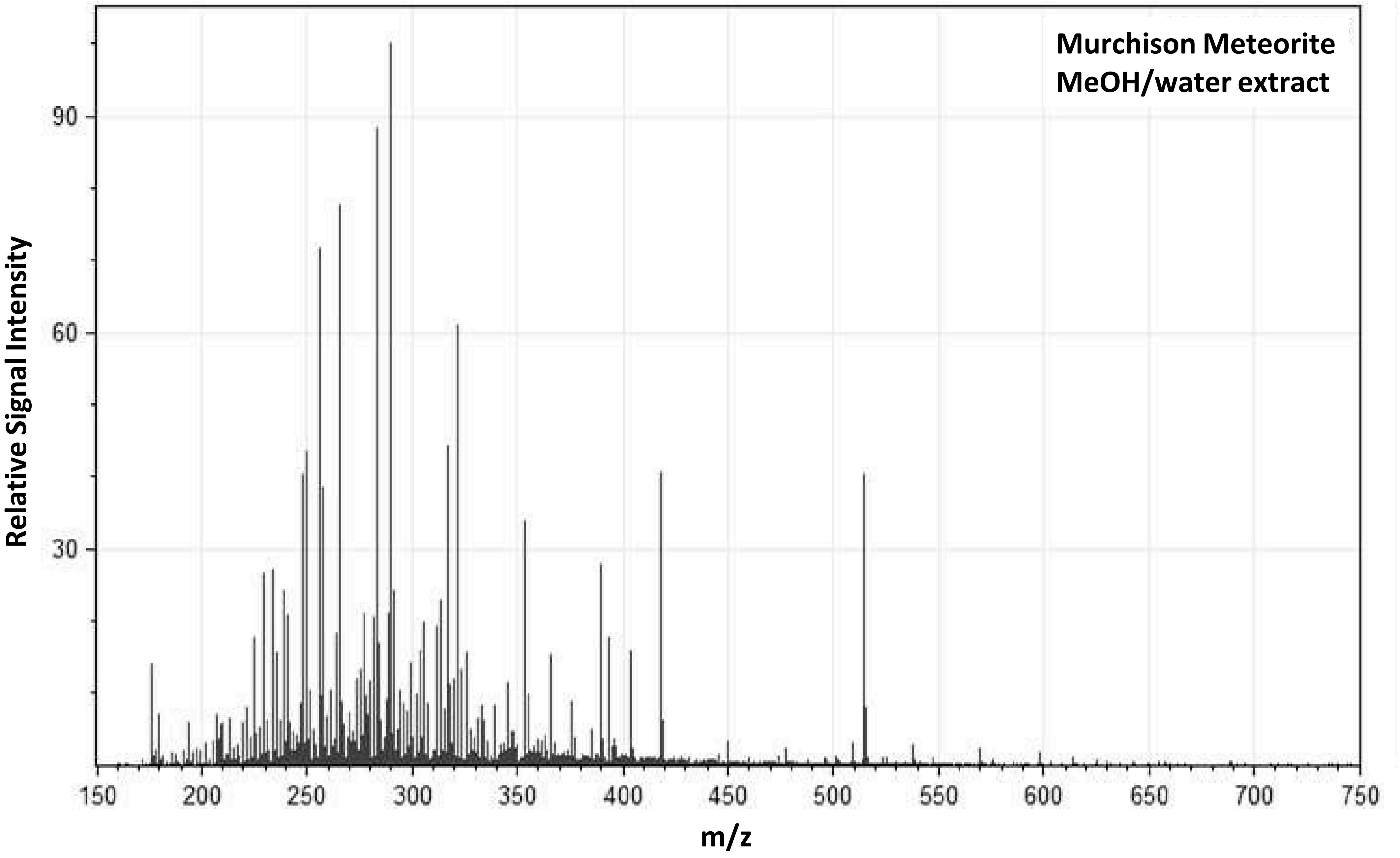

Figure S25B. Odd and even Kendrick Mass Defect plots of the organics extracted using methanol/water of a sample of the Murchison meteorite.

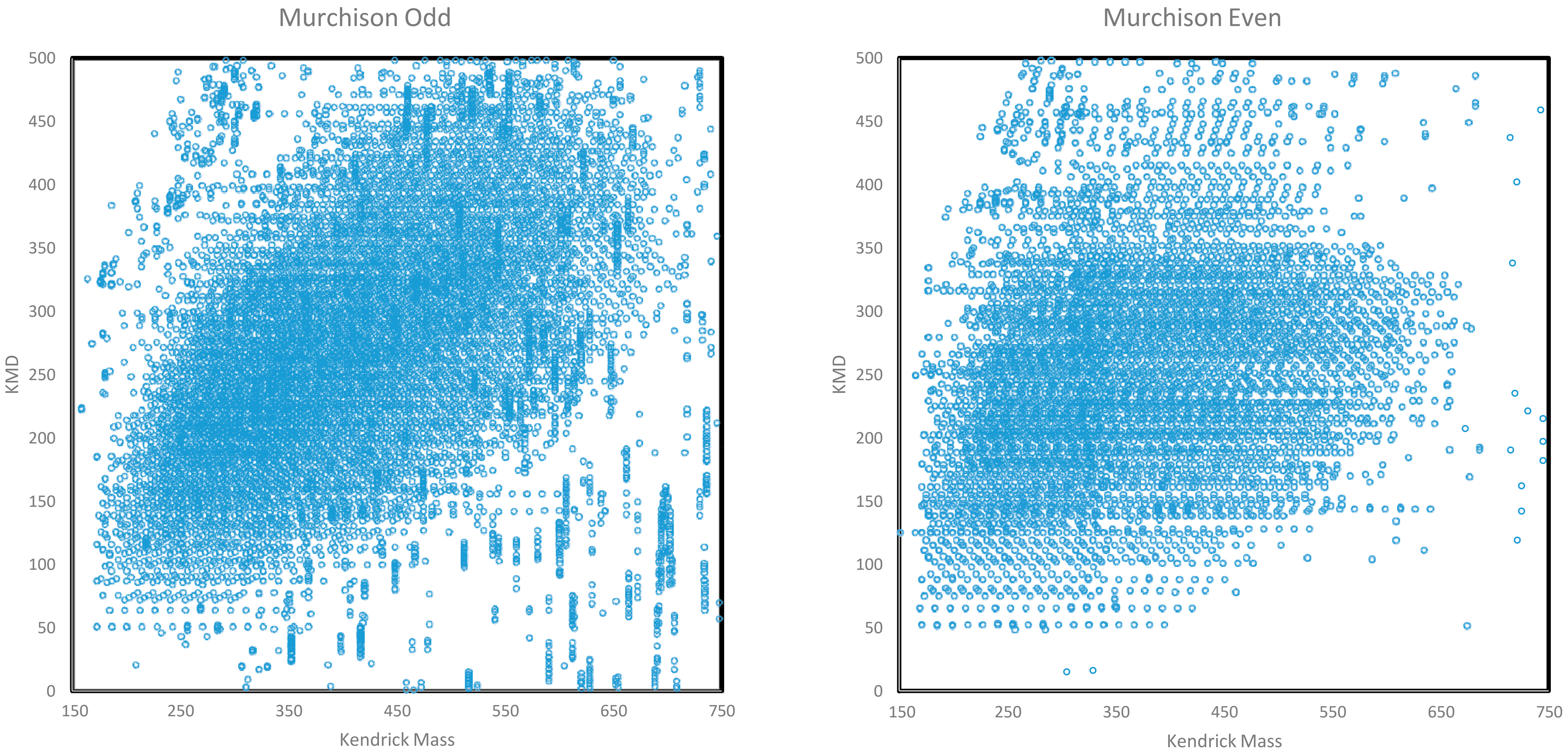

Sample 48 Murchison Meteorite

Figure S26A. Negative mode ESI FT-ICR-MS spectrum of organics extracted using methanol/water of a sample of the Allende meteorite.

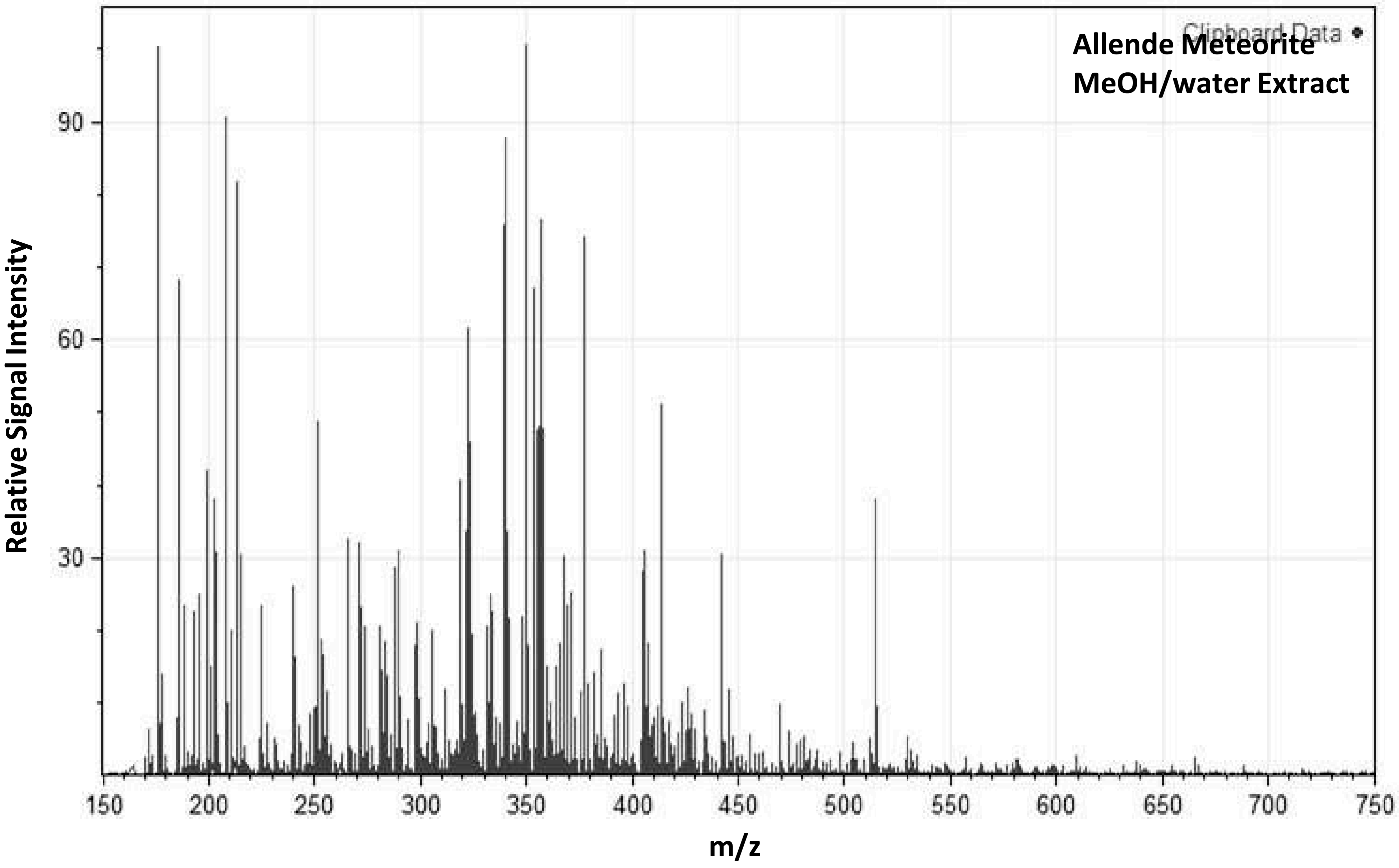

Figure S26B. Odd and even Kendrick Mass Defect plots of the organics extracted using methanol/water of a sample of the Allende meteorite.

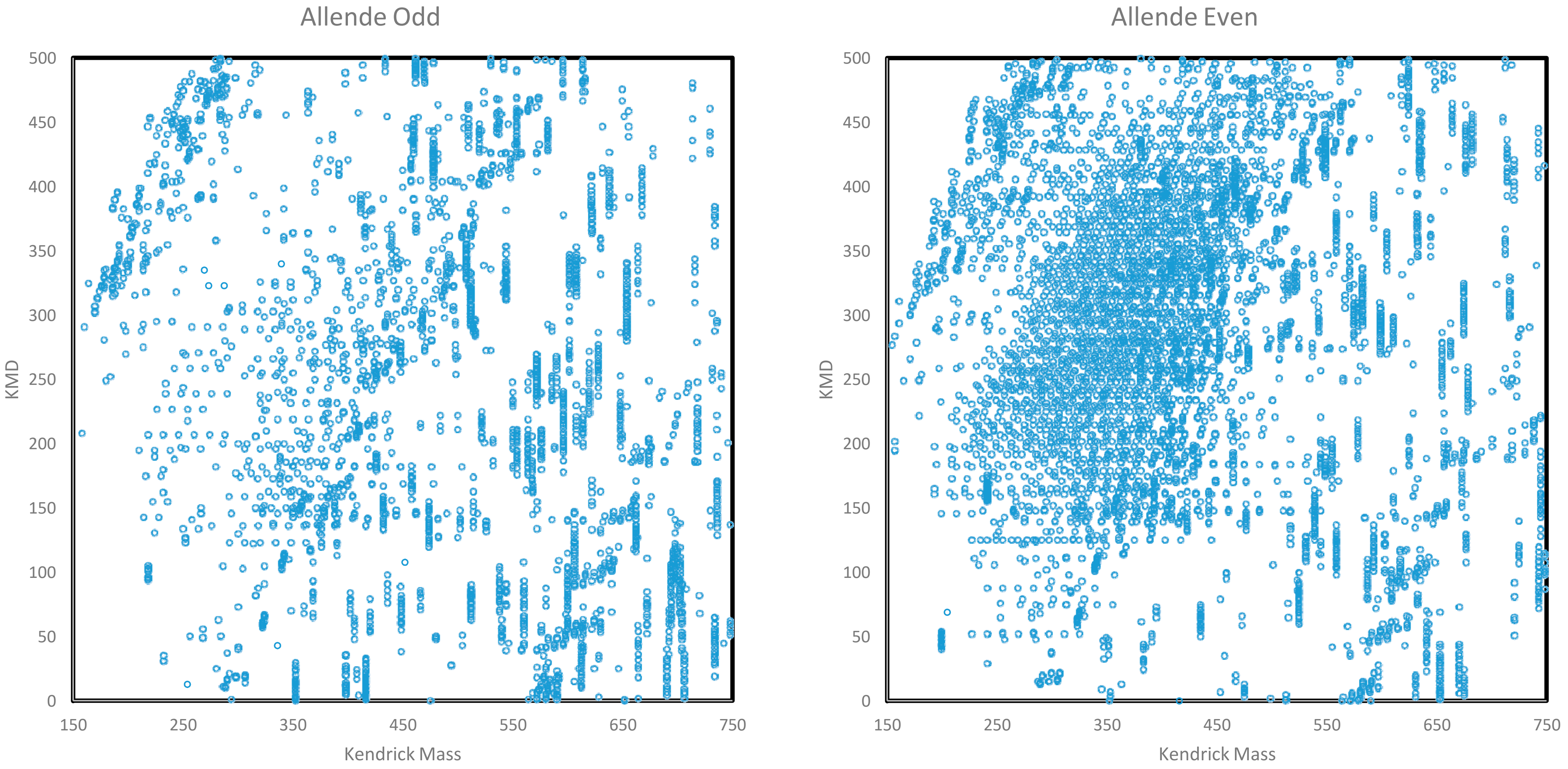

Sample 49 Allende Meteorite

Figure S27A. Negative mode ESI FT-ICR-MS spectrum of organics extracted from the reaction of 2 M paraformaldehyde, 1 M glycolaldehyde, and 0.05 M $Ca(OH)_2$ heated at 85° C for eight days.

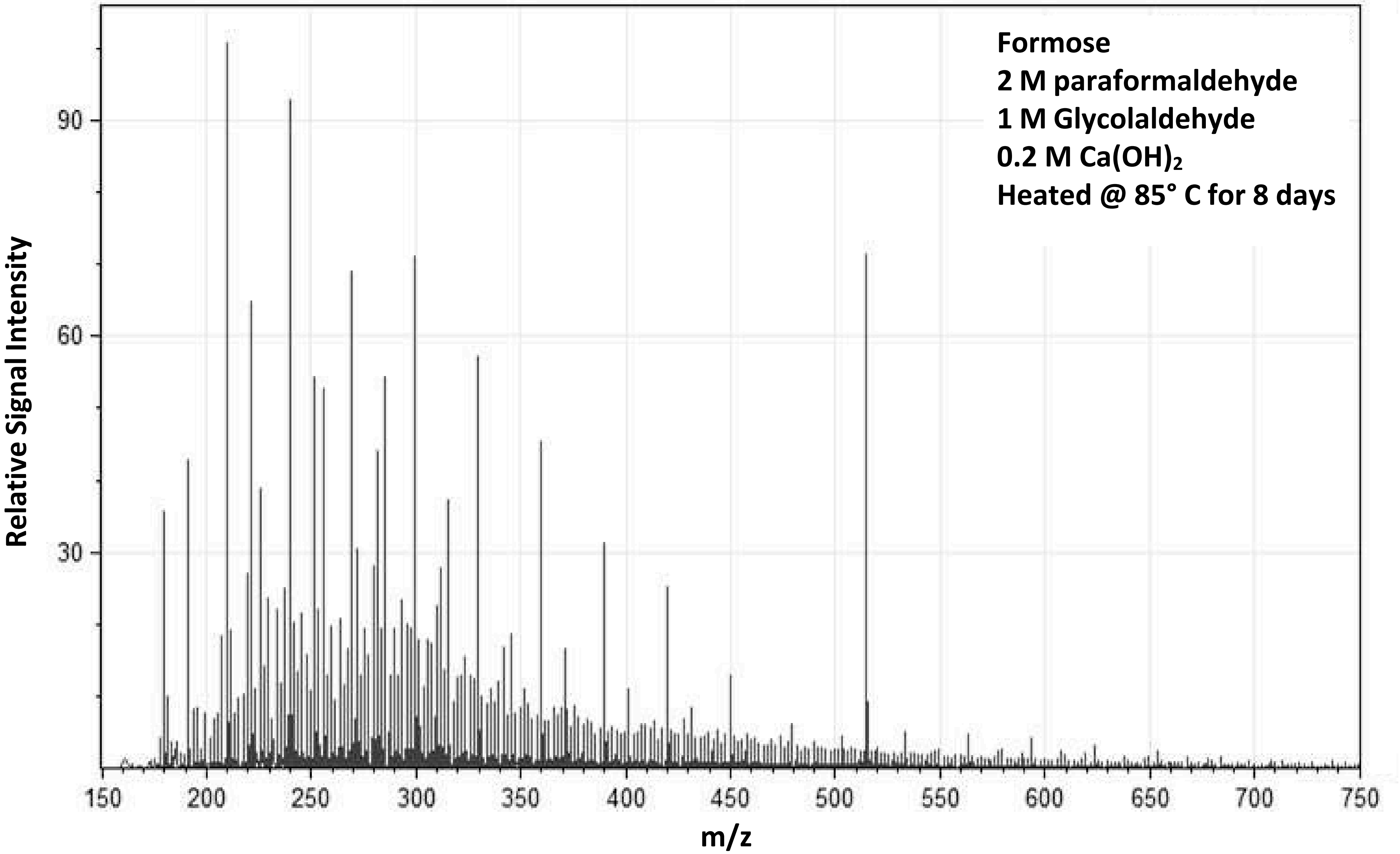

Figure S27B. Odd and even Kendrick Mass Defect plots of the organics extracted from the reaction of 2 M paraformaldehyde, 1 M glycolaldehyde, and 0.05 M $Ca(OH)_2$ heated at 85° C for eight days.

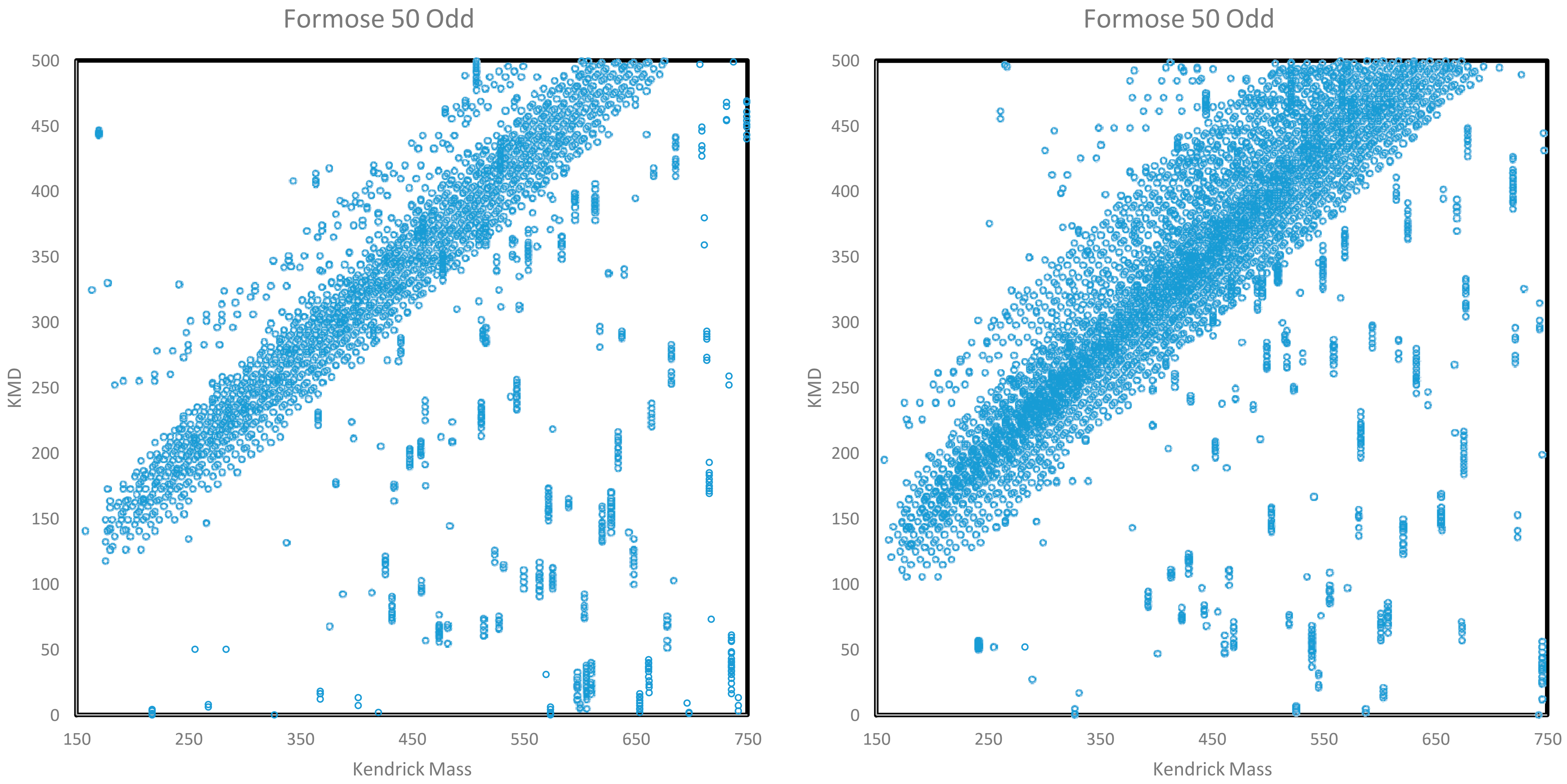

Sample 50 Formose 85° C
